# Global prevalence of different levels of anxiety and stress symptoms in healthcare students: A meta-analysis and meta-regression

**DOI:** 10.1186/s12991-025-00618-1

**Published:** 2026-01-03

**Authors:** Ying Xuan Loh, Ying Lau, Wen Wei Ang, Shean Ern Shannen Lee, Siew Tiang Lau

**Affiliations:** 1 Ng Teng Fong General Hospital, National University Health System, Singapore, Singapore; 2 The Chinese University of Hong Kong, Hong Kong Special Administrative Region 6-8/F, Esther Lee Building, Shatin, New Territories, Hong Kong, China; 3 National University of Singapore, Singapore, Singapore

**Keywords:** Anxiety symptoms, Global prevalence, Healthcare students, Meta-analysis, Stress symptoms

## Abstract

**Background:**

There are limited reviews to report the different levels of anxiety and stress symptoms among students studying nursing, pharmacy, and allied health.

**Objective:**

To calculate the global prevalence of different levels of anxiety and stress symptoms among healthcare students and examine the factors that affect the different levels of prevalence estimates.

**Methods:**

A three-step comprehensive search of 10 databases was conducted. Meta-analysis, subgroup analyses, and meta-regression were performed using the meta package in R software. The Newcastle-Ottawa Scale and Grading of Recommendations Assessments, Development and Evaluation criteria were utilised for the quality appraisal of included studies and the certainty of the evidence, respectively.

**Results:**

A total of 112 studies with 42,331 healthcare students across 43 countries were selected. The prevalences of unspecified, mild, moderate, severe, and extremely severe anxiety symptoms were 41% (95% CI: 33–50), 15% (95% CI: 12–19), 22% (95% CI: 19–26), 10% (95% CI: 8–13), 14% (95% CI: 11–17), respectively. The prevalences of unspecified, mild, moderate, severe, and extremely severe stress symptoms were 36% (95% CI: 25–47), 15% (95% CI: 12–18), 32% (95% CI: 25–40), 11% (95% CI: 8–15), 4% (95% CI: 2–5), respectively. A series of subgroup and meta-regression analyses identified geographic region, use of an instrument, type of healthcare students, sample size and study quality were significantly impacted prevalence estimates.

**Conclusion:**

Findings can contribute as evidence to raising awareness about different levels of anxiety and stress symptoms. Early screening and tailored preventive interventions can help eliminate the prevalence in healthcare students.

**Supplementary Information:**

The online version contains supplementary material available at 10.1186/s12991-025-00618-1.

## Introduction

The field of healthcare education is recognised for its demanding nature, and students enrolled in these courses often experience higher levels of anxiety and stress due to their distinct curricula when compared to their peers at other universities [1], especially nursing, pharmacy, and allied health students [2–4]. In the pre-clinical years, students experience anxiety and stress due to workload, exams, and expectations [5, 6]. To put their education into practice, students in these courses are required to participate in clinical placements; yet the requirements to fulfil them are also a source of stress and anxiety [7]. The impact of anxiety and stress is multifaceted, leading to significant impacts on individuals, and their academic and professional careers. These healthcare students face various psychological consequences as a result of anxiety and stress, including fatigue, mood alterations, and sleep disturbances [5]. Furthermore, the burden of anxiety and stress brings about negative effects on overall academic performance, communication, professionalism, and empathy towards patients [8, 9]. If anxiety or stress symptoms are not recognised and relieved early, these may eventually affect the future workforce of healthcare professionals in providing quality care [10].

The prevalence of anxiety and stress symptoms has been well-researched among medical students [11–13], however, relatively few are known among nursing, pharmacy, dental, public health, and allied health students. Since these students make up more than half of the healthcare student population [14], there is an urgent need to assess the prevalence of anxiety and stress symptoms among these healthcare students. Two systematic reviews [15, 16] were found relating to the prevalence of stress and anxiety among nursing students. However, they have some methodological limitations on a few databases and included studies and a lack of assessment on the certainty of the evidence. None of them investigated the severity of anxiety and stress symptoms and factors affecting prevalence estimates of the different anxiety and stress symptom levels.

A previous study showed that the severity of anxiety and stress symptoms impacts outcomes differently [17]. By providing prevalence estimates on different levels of anxiety and stress symptoms, this valuable information will help healthcare educators improve awareness of the severity and place greater emphasis on different levels of screening, provide preventive intervention, and assist in the establishment of policies or services [15, 18]. The findings of this study provided healthcare educators with an understanding of the severity of these conditions among healthcare students and helped direct future tailored interventions to optimise learning and prepare students to manage their anxiety and stress symptoms effectively [15]. Hence, the objectives of this systematic review were to (1) calculate the global prevalence of different levels of anxiety and stress symptoms among healthcare students and (2) examine the factors that affect the different levels of prevalence estimates.

## Methods

The Preferred Reporting Items for Systematic Reviews and Meta-Analysis Protocols (PRISMA) criteria were followed when conducting this research [19] (Table S1). The study’s protocol was registered under PROSPERO (CRD42023399499).

### Eligibility criteria

To ensure relevant studies are identified, study selection is performed by strict eligibility criteria (Table S2) based on the population, condition and context framework [20] to ensure relevant studies are identified. The inclusion criteria for this review are as follows: (1) studies that were either experimental or observational, (2) included nursing, pharmacy, or allied health students, (3) showed a high prevalence of different severity of anxiety and stress symptoms; the severity was classified into unspecified (did not provide a specific level of anxiety and stress symptoms), mild, moderate, severe, and extremely severe severity is according to the corresponding scales, and (4) used reliable and validated measurement scales with cut-off points. Given that many studies investigated the prevalence of mental health in medical students, we excluded medical students from this review. No limits were placed on the publication year, and the language was limited to English. Published and unpublished studies were included.

### Search strategy

The search strategy and process were guided by a three-phase process as recommended by the Joanna Briggs Institute (JBI) Manual for Evidence Synthesis [21]. The first reviewer (YXL) worked with an experienced medical librarian to build the index and key phrases (Table S3). Eight databases including the Excerpta Medica Database (EMBASE), the Cumulative Index to Nursing and Allied Health Literature (CINAHL), the ProQuest Social Science Database, PsycINFO, PubMed, Scopus, and Web of Science—were searched for studies. Google Scholar and ProQuest Theses and Dissertations were used to look for grey literature. Databases including electronic and gray literature were searched until December 2022. Boolean operators were used to combine important phrases, and appropriate truncations were made. Ultimately, an exhaustive manual search was conducted across pertinent journals and citations to find all relevant papers.

### Study selection and process

EndNote 20 software was used to import and handle the search results [22]. Following the removal of duplicates, the study titles and abstracts were evaluated by two separate reviewers (YXL and SESL). If the two reviewers could not agree, a third reviewer (STL) was consulted. Finally, the complete manuscripts of the residual papers were acquired and evaluated for pertinence. The PRISMA flow diagram showed the outcomes of the selection procedure.

### Data extraction

The data extraction form was modified based on the JBI Manual for Evidence Synthesis for Systematic Reviews of Prevalence and Incidence Data [20]. Data extracted included the last name of the first author, year of publication, geographical regions, study design, nature of the population, portion of gender, mean age, sample size, response rate, measurement tools used to assess anxiety or stress symptoms, and cut-off scores used. The global prevalence of anxiety or stress symptoms with five different severities was included, namely unspecific, mild, moderate, severe, and extremely severe. The period of data collection was before or after COVID-19. Pilot testing of the form was done on five studies to identify any possible missing data, and adaptations were made accordingly. Two independent reviewers (YXL and SESL) carried out the data extraction.

### Quality assessment

The Risk of Bias Tool (ROB) version 2 was used for experimental studies [23]. The Newcastle Ottawa Scale (NOS) was used to rate cross-sectional, cohort, or case-control studies [24]. A study is considered high quality if it is rated more than 6 stars, while a low-quality study would have 6 stars or less. Two independent reviewers (YXL and SESL) carried out the quality assessment. If an agreement could not be reached, a third author (YL) was consulted. The Grading of Recommendation, Assessment, Development, and Evaluation (GRADE) criteria were used to rate the overall certainty of evidence [25, 26]. The criteria look at the risk of bias, inconsistency, indirectness, imprecision, and publication bias. The GRADE was rated from very low to high. Publication bias was analysed using a funnel plot and Egger’s regression test [27, 28]. Evidence of publication bias was found if the asymmetry of the funnel plot and *p*-value of the Egger test were < 0.05.

To guarantee consistency between the two reviewers for the selection of articles, individual quality, and overall quality of evidence ratings, inter-rater reliability was determined using Cohen’s kappa statistic (*κ*). According to McHugh (2012), values ranging from 0.01 to 0.20 were considered to indicate none to minor agreement, 0.21–0.40 to be fair, 0.41–0.60 to be moderate, 0.61–0.80 to be considerable, and 0.81–1.00.81.00 to be virtually perfect agreement [29].

### Data synthesis

Statistical analyses for this review were conducted using the meta package in R software (Version 4.3.1) [30, 31]. Meta-analysis was performed where the prevalence of anxiety and stress symptoms retrieved from included studies were pooled and reported with 95% confidence intervals (CI). The prevalence (P) of stress and anxiety symptoms was calculated using the following equation:

$$\begin{aligned}&\:\mathrm{Prevalence}\:{(P)}\:\\&\quad=\:\frac{\text{number of }\text{healthcare students}\text{ anxiety }\mathrm{stress}\,\text{}\mathrm{symptoms}\text{}\mathrm{(n)}}{\mathrm{t}\mathrm{o}\mathrm{t}\mathrm{a}\mathrm{l}\:\mathrm{n}\mathrm{u}\mathrm{m}\mathrm{b}\mathrm{e}\mathrm{r}\:\mathrm{o}\mathrm{f}\:\mathrm{h}\mathrm{e}\mathrm{a}\mathrm{l}\mathrm{t}\mathrm{h}\mathrm{c}\mathrm{a}\mathrm{r}\mathrm{e}\:\mathrm{s}\mathrm{t}\mathrm{u}\mathrm{d}\mathrm{e}\mathrm{n}\mathrm{t}\mathrm{s}\:\mathrm{s}\mathrm{a}\mathrm{m}\mathrm{p}\mathrm{l}\mathrm{e}\mathrm{d}\:\left(\mathrm{N}\right)},\end{aligned}$$

where the number of healthcare students with anxiety or stress symptoms (n) was divided by the total number of healthcare samples (N) [32]. The prevalence estimates of anxiety and stress symptoms were calculated using a random effect model guided by the Hartung-Knapp-Sidik-Jonkman (HKSJ) method [33]. The Freeman-Tukey double arcsine transformation was used [34]. The random effects model was chosen as it involves an assumption of estimated effects from various studies [35]. Heterogeneity between pooled studies was reported using Cochran’s Q and *I*^*2*^ statistics. The interpretation of *I*^*2*^ values is as follows: 75% to 100% suggest considerable heterogeneity; 50% to 90% suggest substantial heterogeneity; 30% to 60% suggest moderate heterogeneity; 0% to 40% might not be important [35].

### Additional data analysis

For categorical and continuous covariates, meta-regression and subgroup analyses were used to investigate the sources of heterogeneity [36]. Subgroups were categorised according to types of students (nursing vs. non-nursing), type of measurement tools (DASS vs. non-DASS), and geographical regions (Africa/Middle East, America, Asia Pacific, Europe, or Oceania), and data collection period (pre- vs. post-COVID pandemic). A *p*-value of < 0.1 indicates the subgroup analysis has a statistically significant difference between subgroups [37]. Meta-regression was performed to examine the effects of covariates on study effect size. The estimate (*β*) indicates the strength and direction of the relationship between our outcome (global prevalence and severity of anxiety or stress symptoms) and the continuous covariates (NOS scores, sample size, and publication year). We adopted *p*-values of 0.05 to indicate a significant effect of meta-regression analysis [38].

## Results

### Search results

**Fig. 1:**
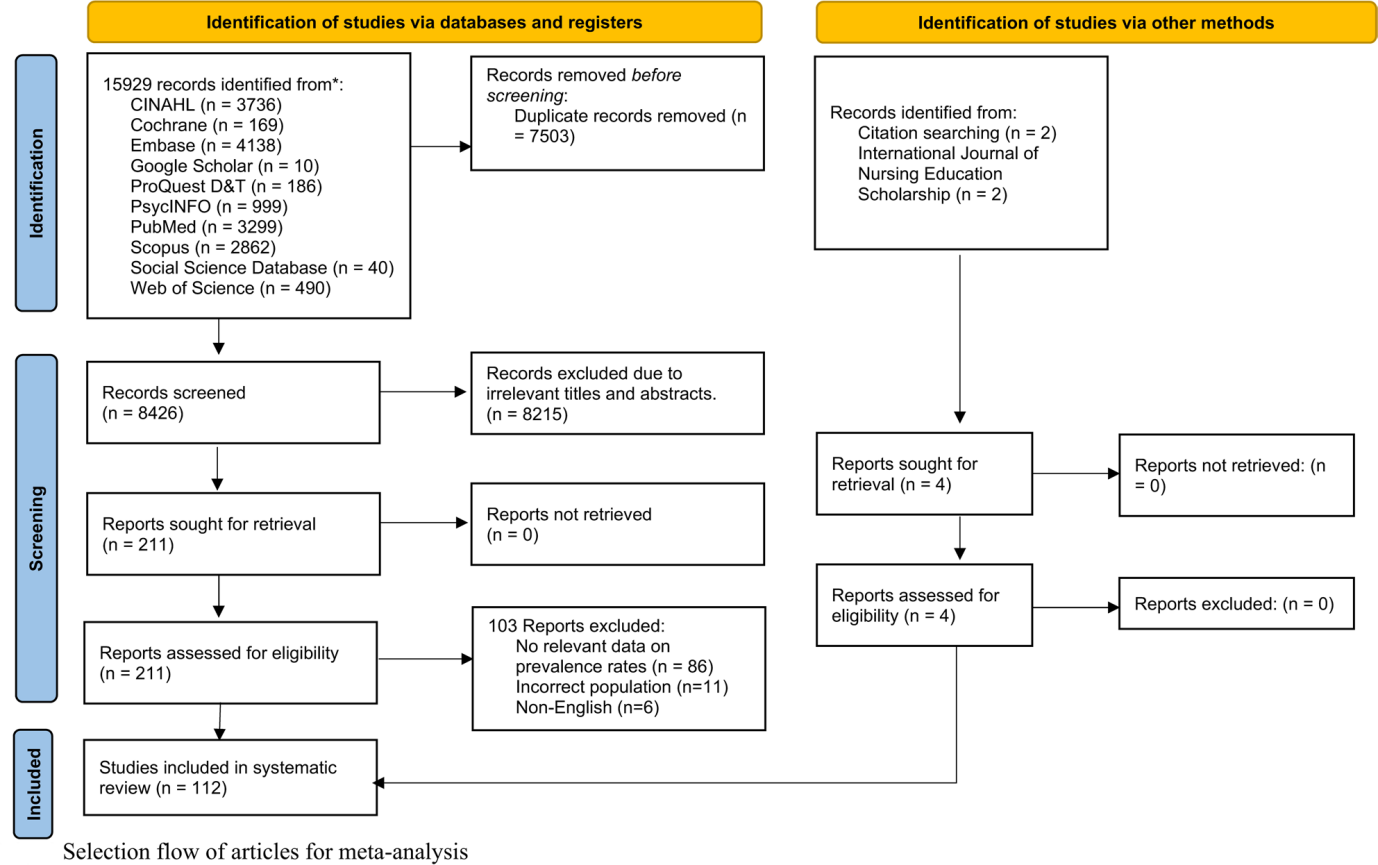
Selection flow of articles for meta-analysis

Ten databases were searched, and the results showed 15,929 records, as shown in the PRISMA flowchart (Fig. 1). An additional four studies were found by searching for citations and target journals. Using EndNote 20 software, duplicates were eliminated, and 8,215 studies’ titles and abstracts were screened following eligibility requirements. The remaining 211 articles’ full texts were examined. One hundred and three reports (Table S4) were excluded because they lacked prevalence information, had the wrong population, or were non-English. Finally, a total of 112 studies [39–150] were included.

### Study characteristics

Table 1 displays the characteristics of 112 studies totalling 42,331 healthcare students from 20 healthcare courses in 43 countries. Sample sizes varied between 29 [141] and 6,348 [96], and the mean age varied between 18.8 [49] and 38 [59] years old. Asia accounted for 64 studies (57.14%), followed by America (number of studies, *k* = 25, 22.32%), Europe (*k* = 13, 11.61%), Africa/Middle East (*k* = 6, 5.36%), and Oceania (*k* = 4, 3.57%). Anxiety and stress symptoms were measured on difficult scales, and one third of them (33.03%) used scale was the 21-item Depression Anxiety Stress Scale (DASS-21) [151]. Two subscales of DASS measure both anxiety and stress symptoms in individuals [151].

**Table 1:** Characteristics of included studies

Authors (Year)	Country	Study design	Type of students	Gender (%)	Age (Mean/median/range)	Sample size (*n*)	Response rate (%)	Outcome	Scale	Cut-off	Before/after COVID
Abas. (2017)	Iraq	Cross-sectional	Nursing	M: 16 F: 84	NR	100	100	Anxiety	BAI	NR	Before
Abbas et al. (2021)	Pakistan	Cross-sectional	Physiotherapy	M: 43.1 F: 56.9	Mean: 23.33	188	100	Anxiety	GAD-7	GAD-7 ≥ 5	Before
Ajmal et al. (2018)	Saudi Arabia	Cross-sectional	Dental	M: NR F: NR	NR	122	100	Anxiety	HAD	HAD ≥ 8	Before
Alaaddin et al. (2021)	Saudi Arabia	Cross-sectional	Pharmacy	M: 32.5 F: 67.5	NR	400	100	Anxiety Stresss	DASS-21 (A) DASS-21 (S)	NR	Before
Alahmary et al. (2019)	Saudi Arabia	Cross-sectional	Dental	M: 8.6 F: 91.4	Range: 20–35	105	NR	Anxiety	HADS (A)	HADS (A) ≥ 11	Before
Albani et al. (2022)	Greece	Cross-sectional	Nursing	M: 13.5 F: 86.5	Mean: 22.8	200	NR	Anxiety	HADS (A)	HADS (A) ≥ 8	After
Alici et al. (2020)	Turkey	Cross-sectional	Nursing	M: 32.1 F: 67.9	Mean: 20.12	234	78	Anxiety	BAI	BAI ≥ 10	After
Alsairafi et al. (2021)	Kuwait	Cross-sectional	Healthcare students	M: 10.4 F: 89.6	NR	298	NR	Anxiety	GAD-7	GAD-7 ≥ 5	After
Alsolais et al. (2021)	Saudi Arabia	Cross-sectional	Nursing	M: 44.3 F: 55.7	Mean: 21.77	492	46.5	Anxiety Stress	DASS-21 (A) DASS-21 (S)	NR	After
Aluh et al. (2020)	Nigeria	Cross-sectional	Pharmacy	M: 49.3 F: 50.7	Mean: 22.57	408	13.32	Anxiety Stress	DASS-21 (A) DASS-21 (S)	DASS-21 (A) ≥ 8 DASS-21 (S) ≥ 15	Before
Amr et al. (2011)	Egypt	Cross-sectional	Nursing	M: NR F: NR	Mean: 18.8 Range: 17–22	373	92.8	Anxiety Stress	HAD PSS	HAD ≥ 12 NR	Before
Asturias et al. (2021)	Australia	Cross-sectional	Nursing	M: 18.1 F: 81.9	NR	377	62	Stress	PSS	PSS ≥ 14	Before
Azodo et al. (2013)	Nigeria	Cross-sectional	Dental	M: NR F: NR	NR	54	88	Stress	ISMA Stress Questionnaire	ISMA Stress Questionnaire ≥ 5	Before
Banstola et al. (2021)	Nepal	Cross-sectional	Nursing	M: 0 F: 100	Mean: 19.55 Range: 16–22	144	95	Anxiety	BAI	BAI ≥ 11	After
Barroso-Corroto et al. (2021)	Spain	Cross-sectional	Nursing, Midwifery	M: 13.3 F: 86.7	Mean: 21.27 Range: 18–37	248	NR	Anxiety	HADS (A)	NR	After
Baruah et al. (2022)	India	Cross-sectional	Nursing	M: NR F: NR	Mean: 20.8 Range: 17–24	214	100	Anxiety Stress	DASS-21 (A) DASS-21 (S)	DASS-21 (A) ≥ 8 DASS-21 (S) ≥ 15	After
Basudan et al. (2017)	Saudi Arabia	Cross-sectional	Dental	M: 54.2 F: 45.7	NR	277	95.8	Anxiety Stress	DASS-21 (A) DASS-21 (S)	NR	Before
Belingheri et al. (2021)	Italy	Cross-sectional	Nursing	M: 29.7 F: 70.3	Mean: 22	202	78	Stress	GHQ-12	GHQ-12 ≥ 5	Before
Bhatt et al. (2022)	India	Cross-sectional	Dental	M: 21.3 F: 78.8	Median: 21	320	NR	Anxiety Stress	DASS-21 (A) DASS-21 (S)	DASS-21 (A) ≥ 8 DASS-21 (S) ≥ 15	Before
Braz-José et al. (2022)	Portugal	Cross-sectional	Dental	M: 21.8 F: 78.2	NR	1115	38	Anxiety Stress	DASS-21 (A) DASS-21 (S)	DASS-21 (A) ≥ 7 DASS-21 (S) ≥ 11	After
Brown et al. (2016)	Jamaica	Cross-sectional	Nursing	M: NR F: NR	Mean: 38 Range: 24–56	57	73	Stress	PSS-14	PSS-14 ≥ 24	Before
Campos et al. (2021)	Brazil	Cross-sectional	Pharmacy	M: 24.2 F: 75.8	Mean: 21.7	66	22.4	Anxiety Stress	DASS-21 (A) DASS-21 (S)	DASS-21 (A) ≥ 8 DASS-21 (S) ≥ 15	After
Chernomas et al. (2013)	Canada	Cross-sectional	Nursing	M: 11 F: 89	NR	437	49.5	Anxiety Stress	DASS-42 (A) DASS-42 (S)	NR	Before
Cheung et al. (2016)	Hong Kong	Cross-sectional	Nursing	M: 27.5 F: 72.5	Range: 18–30	661	52.6	Anxiety Stress	DASS-21 (A) DASS-21 (S)	DASS-21 (A) ≥ 10 DASS-21 (S) ≥ 19	Before
										*(Continued on next page)*	
Chouhan et al. (2021)	India	Cross-sectional	Nursing	M: NR F: NR	Mean: 21.16 Range: 18–27	223	57.62	Anxiety Stress	DASS-42 (A) DASS-42 (S)	DASS-42 (A) ≥ 8 DASS-42 (S) ≥ 15	After
Chutipattana et al. (2022)	Thailand	Cross-sectional	Public Health	M: 11.7 F: 88.3	Mean: 19.85	463	92.6	Anxiety Stress	DASS-21 (A) DASS-21 (S)	DASS-21 (A) ≥ 8 DASS-21 (S) ≥ 15	After
Cici et al. (2020)	Turkey	Cross-sectional	Nursing	M: NR F: NR	Mean: 20.8	322	69.8	Anxiety	BAI STAI-I	BAI ≥ 16 STAI-I ≥ 40	After
Coelho et al. (2021)	Brazil	Cross-sectional	Nursing	M: 14.07 F: 85.93	Mean: 21.44	192	100	Anxiety	BAI	BAI ≥ 11	Before
Daya. (2021)	South Africa	Cross-sectional	Nursing	M: 33.9 F: 66.1	Range: 18–39	127	84.7	Anxiety Stress	DASS-42 (A) DASS-42 (S)	DASS-42 (A) ≥ 8 DASS-42 (S) ≥ 15	After
Devi et al. (2021)	Indonesia	Cross-sectional	Nursing	M: 28.3 F: 71.7	Mean: 23.15 Range: 20–33	336	94.1	Anxiety Stress	DASS-42 (A) DASS-42 (S)	DASS-42 (A) ≥ 8 DASS-42 (S) ≥ 15	Before
Diaz-Godiño et al. (2019)	Peru	Cross-sectional	Nursing	M: 86.2 F: 13.8	NR	1193	100	Anxiety Stress	DASS-21 (A) DASS-21 (S)	NR	Before
Díaz-Jiménez et al. (2020)	Spain	Cross-sectional	Social work	M: 9.9 F: 90.1	Mean: 23.22 Range: 18–27	365	20	Anxiety	DASS-21 (A)	NR	After
Dubovi et al. (2022)	Israel	Cross-sectional	Nursing	M: 12 F: 88	Mean: 22.8	135	NR	Anxiety	STAI-S	STAI-S ≥ 40	After
Fabbris et al. (2017)	Brazil	Cross-sectional	Nursing	M: 9.5 F: 90.5	Mean: 21	169	100	Anxiety	BAI	BAI ≥ 11	Before
Faraj. (2022)	Iraq	Cross-sectional	Nursing	M: 50 F: 50	Range: 18–26	60	100	Stress	PSS-10	PSS-10 ≥ 20	After
Fauzi et al. (2021)	Malaysia	Cross-sectional	Medical Laboratory Sciences, Medical Imaging, Nursing	M: 9.9 F: 90.1	Mean: 21.86 Range: 19–28	449	93.9	Anxiety Stress	DASS-21 (A) DASS-21 (S)	DASS-21 (A) ≥ 8 DASS-21 (S) ≥ 15	Before
Fernandez et al. (2021)	Brazil	Cross-sectional	Dental	M: 29.4 F: 70.6	Mean: 23.27	1050	NR	Anxiety	GAD-7	GAD-7 ≥ 5	After
Fischbein et al. (2019)	United States	Cross-sectional	Pharmacy	M: 40.7 F: 59.3	Mean: 26.7	159	NR	Anxiety	GAD-7	GAD-7 ≥ 10	Before
Frajerman et al. (2022)	France	Cross-sectional	Dental, Nursing	M: 26.5 F: 72.5	NR	328	10.3	Anxiety	HADS (A)	HADS (A) ≥ 11	After
Gao et al. (2021)	China	Cross-sectional	Nursing	M: 11.8 F: 88.2	Mean: 19.95	1532	86.1	Anxiety Stress	DASS-21 (A) DASS-21 (S)	DASS-21 (A) ≥ 8 DASS-21 (S) ≥ 15	After
Gautam et al. (2020)	India	Cross-sectional	Physiotherapy	M: 35.24 F: 64.76	Range: 17–25	105	NR	Anxiety Stress	DASS-21 (A) DASS-21 (S)	NR	Before
George et al. (2018)	India	Cross-sectional	Dental	M: 27 F: 73	NR	100	100	Stress	PSS-10	PSS-10 ≥ 14	Before
George et al. (2022)	Malaysia	Cross-sectional	Dental	M: 68.7 F: 31.3	Mean: 22.38	351	100	Anxiety Stress	DASS-21 (A) DASS-21 (S)	DASS-21 (A) ≥ 8 DASS-21 (S) ≥ 15	Before
Gitay et al. (2019)	Pakistan	Cross-sectional	Health Sciences	M: 38.3 F: 61.7	NR	300	NR	Anxiety	GAD-7	NR	Before
Hasanpour et al. (2021)	Iran	Cross-sectional	Nursing	M: 27 F: 73	Mean: 23.07 Range: 18–43	174	100	Anxiety	GAD-7	GAD-7 ≥ 10	After
Helen et al. (2013)	India	Cross-sectional	Nursing	M: 23 F: 77	NR	200	NR	Stress	Stress Rating Scale	NR	Before
Hodselmans et al. (2018)	Sweden and Netherlands	Cross-sectional	Physiotherapy	M: 32.8 F: 57.8	NR	116	NR	Stress	SSI	NR	Before
Jardon et al. (2022)	United States	Cross-sectional	Nursing	M: 10.9 F: 89.1	NR	182	30.1	Anxiety Stress	GAD-7 COVID Stress Scales	GAD-7 ≥ 10 COVID Stress Scale ≥ 8	After
										*(Continued on next page)*	
Javeth. (2018)	India	Cross-sectional	Nursing	M: 3 F: 97	NR	150	100	Stress	MESSA	MESSA ≥ 36	Before
Jiménez-Ortiz et al. (2019)	Mexico	Cross-sectional	Dental	M: 35.62 F: 64.38	NR	73	NR	Stress	PSS-14	PSS-14 ≥ 22	Before
Juneja et al. (2021)	India	Cross-sectional	Dental	M: 35.2 F: 64.8	NR	250	81	Stress	DES CAS PSS	NR	After
Kalkan Uğurlu et al. (2020)	Turkey	Cross-sectional	Nursing	M: 20.7 F: 79.3	Mean: 20.6 Median: 21 Range: 18–33	411	93.83	Anxiety Stress	DASS-42 (A) DASS-42 (S)	DASS-42 (A) ≥ 8 DASS-42 (S) ≥ 15	After
Keskin. (2021)	Turkey	Cross-sectional	Dental	M: 39.8 F: 60.2	Range: 20–25	259	100	Anxiety Stress	DASS-42 (A) DASS-42 (S)	DASS-42 (A) ≥ 8 DASS-42 (S) ≥ 15	After
Khorassani et al. (2021)	New York	Cross-sectional	Pharmacy	M: 27 F: 73	NR	198	NR	Anxiety	SAS	SAS ≥ 36	Before
Killinger et al. (2017)	North America	Cross-sectional	Veterinary Medicine	M: 11.2 F: 88.4	Mean: 25.62 Range: 25.62	1245	89.9	Stress	VMSI	VMSI ≥ 4	Before
Krifa et al. (2022)	Tunisia	Cross-sectional	Podiatry, Emergency Care, Operating Instrumentation, Paediatric Care, Research Masters	M: 6 F: 94	NR	366	88.8	Anxiety Stress	DASS-21 (A) DASS-21 (S)	DASS-21 (A) ≥ 8 DASS-21 (S) ≥ 15	After
Larijani et al. (2010)	Iran	Cross-sectional	Nursing, Midwifery	M: 27.2 F: 72.8	NR	250	100	Anxiety	STAI	STAI ≥ 20	Before
Li et al. (2021)	China	Cross-sectional	Nursing	M: 9.63 F: 90.37	NR	6348	97.66	Anxiety	GAD-7	GAD-7 ≥ 5	After
Lingawi et al. (2020)	Saudi Arabia	Cross-sectional	Dental	M: 40.3 F: 59.7	Range: 18–26	258	86	Anxiety	GAD-7	GAD-7 ≥ 5	After
Ma et al. (2022)	China	Cross-sectional	Nursing	M: 7.1 F: 92.9	Mean: 19.82	197	98.5	Stress	PSS-10	PSS-10 ≥ 14	After
Marcen-Roman et al. (2021)	Spain	Cross-sectional	Nursing, Physiotherapy, Occupational Therapy	M: 18.3 F: 81.7	Mean: 21.02	252	NR	Anxiety Stress	GADS PSS-10-C	GADS ≥ 4 PSS-10-C ≥ 25	After
Mardea et al. (2020)	Indonesia	Cross-sectional	Pharmacy	M: NR F: NR	NR	487	98	Stress	PSS-10	PSS-10 ≥ 14	Before
Masha’al et al. (2022)	Jordan	Cross-sectional	Nursing	M: 25.9 F: 74.1	Mean: 20.08	282	70.5	Anxiety	GAD-7	GAD-7 ≥ 5	After
Masilamani et al. (2019)	Malaysia	Cross-sectional	Nursing	M: 8.3 F: 91.7	Mean: 20.2	96	77.4	Stress	GHQ-12	NR	Before
Meckamalil et al. (2022)	Canada	Cross-sectional	Chiropractic	M: 39.8 F: 60.2	Mean: 25	510	67	Anxiety Stress	DASS-21 (A) DASS-21 (S)	DASS-21 (A) ≥ 8 DASS-21 (S) ≥ 15	Before
Melo et al. (2021)	Brazil	Cross-sectional	Nursing	M: 13.4 F: 86.6	Mean: 19.8 Median: 19 Range: 18–39	82	91.1	Anxiety	HADS (A)	HADS (A) ≥ 9	Before
Mohebbi et al. (2019)	Iran	Cross-sectional	Nursing	M: 34.9 F: 65.1	Range: 19–29	130	73.9	Anxiety	GHQ-28	NR	Before
Moreira et al. (2013)	Brazil	Cross-sectional	Nursing	M: NR F: NR	Mean: 23.65 Range: 21–33	88	NR	Stress	PSS-10	PSS-10 ≥ 18.7	Before
Nahar et al. (2019)	United States	Cross-sectional	Veterinary Medicine	M: 11.7 F: 88.3	Mean: 25.3	264	77.2	Anxiety	PHQ-4	NR	Before
										*(Continued on next page)*	
Nahm et al. (2021)	Korea	Cross-sectional	Veterinary Medicine	M: 52 F: 48	NR	1063	32.5	Anxiety Stress	DASS-21 (A) DASS-21 (S)	NR	Before
Nebhinani et al. (2020)	India	Cross-sectional	Nursing	M: NR F: NR	Mean: 20.19	221	96.9	Stress	SNSI	SNSI ≥ 22	Before
Pandey et al. (2015)	Nepal	Cross-sectional	Nursing	M: NR F: NR	Mean: 20.44 Range: 16–27	190	100	Stress	SAAS	SAAS ≥ 16	Before
Pate et al. (2021)	United States	Cross-sectional	Pharmacy	M: 42 F: 58	NR	119	46	Anxiety	CTAS-2	CTAS-2 ≥ 44	Before
Patten et al. (2021)	United States	Cross-sectional	Didactic Program in Dietetics	M: 8.2 F: 91.8	NR	611	13	Anxiety Stress	DASS-21 (A) DASS-21 (S)	NR	Before
Pryjmachuk et al. (2006)	England	Cross-sectional	Midwifery	M: 0 F: 100	NR	102	85	Stress	GHQ-12	GHQ-12 ≥ 4	Before
Racic et al. (2017)	Bosnia and Herzegovina	Cross-sectional	Dental, Nursing, Speech Therapy	M: 30.8 F: 69.2	Mean: 21.5	279	70.5	Stress	PSS-14	PSS-14 ≥ 29	Before
Radeef et al. (2020)	Malaysia	Cross-sectional	Pharmacy	M: 14.3 F: 85.7	NR	223	NR	Anxiety Stress	DASS-21 (A) DASS-21 (S)	NR	Before
Rashmi et al. (2022)	India	Cross-sectional	Nursing	M: 22.9 F: 77.1	NR	175	NR	Stress	PSS-10	PSS-10 ≥ 14	After
Ratanasiripong et al. (2012)	Thailand	Cross-sectional	Nursing	M: 16 F: 84	Mean: 22.8 Range: 20–31	110	100	Anxiety	STAI	NR	Before
Rathnayake et al. (2016)	Sri Lanka	Cross-sectional	Nursing	M: 30.4 F: 69.6	Mean: 24.1 Range: 21–27	92	86.4	Anxiety Stress	DASS-21 (A) DASS-21 (S)	NR	Before
Reghuram et al. (2014)	India	Cross-sectional	Nursing	M: NR F: NR	NR	1000	100	Anxiety	SAS	NR	Before
Rezaei et al. (2020)	Iran	Cross-sectional	Midwifery	M: 0 F:100	NR	70	64.8	Stress	PSS-14	PSS-14 ≥ 15	Before
Rodriguez-Roca et al. (2021)	Spain	Cross-sectional	Nursing, Physiotherapy, Occupational Therapy	M: 18.3 F: 81.7	Mean: 21.02	252	NR	Anxiety Stress	GADS PSS-10-C	GADS ≥ 4 PSS-10-C ≥ 25	After
Roldan-merino et al. (2021)	Spain	Cross-sectional	Nursing	M: 12.8 F: 87.2	Mean: 24.7	203	NR	Anxiety	GAD-7	GAD-7 ≥ 10	After
Rosenthal, et al. (2021)	United States	Cross-sectional	Nursing	M: 8 F: 92	NR	222	30	Anxiety Stress	DASS-21 (A) DASS-21 (S)	NR	After
Sabih et al. (2013)	Pakistan	Cross-sectional	Physiotherapy	M: 32.5 F: 67.5	NR	231	92.4	Stress	SLSI	NR	Before
Sahu et al. (2019)	India	Cross-sectional	Nursing	M: 5.9 F: 94.1	Mean: 25.3	102	92.7	Stress	PSS-10	PSS-10 ≥ 14	Before
Sakai et al. (2021)	Japan	Cross-sectional	Nursing	M: NR F: NR	Range: 20–22	104	100	Anxiety	HADS (A)	HADS (A) ≥ 8	After
Saldanha et al. (2021)	India	Cross-sectional	Nursing	M: 7.8 F: 92.2	Range: 17–22	205	100	Stress	PSS	PSS ≥ 14	After
Samreen et al. (2020)	Saudi Arabia	Cross-sectional	Pharmacy	M: 100 F: 0	Range: 18–30	170	NR	Anxiety	GAD-7	GAD-7 ≥ 5	Before
Samson. (2019)	Nepal	Cross-sectional	Nursing	M: NR F: NR	Mean: 20.29	680	99.7	Anxiety Stress	DASS-21 (A) DASS-21 (S)	DASS-21 (A) ≥ 8 DASS-21 (S) ≥ 15	Before
Savitsky et al. (2020)	Israel	Cross-sectional	Nursing	M: NR F: NR	NR	215	88	Anxiety	GAD-7	GAD-7 ≥ 10	After
Shdaifat et al. (2020)	Saudi Arabia	Cross-sectional	Nursing	M: 35.2 F: 64.8	NR	54	NR	Anxiety Stress	DASS-42 (A) DASS-42 (S)	DASS-42 (A) ≥ 8 DASS-42 (S) ≥ 15	Before
										*(Continued on next page)*	
Sogut et al. (2020)	Turkey	Cross-sectional	Midwifery	M: 0 F: 100	Mean: 20.79 Range: 18–38	972	9.7	Anxiety	BAI	BAI ≥ 22	After
Stanton et al. (2021)	Australia	Cross-sectional	Nursing	M: 5.6 F: 94.4	NR	500	NR	Anxiety Stress	DASS-21 (A) DASS-21 (S)	DASS-21 (A) ≥ 8 DASS-21 (S) ≥ 15	Before
Stormon et al. (2019)	Australia	Cross-sectional	Dental	M: 42.5 F: 57.5	NR	179	83.6	Anxiety Stress	DASS-21 (A) DASS-21 (S)	DASS-21 (A) ≥ 8 DASS-21 (S) ≥ 15	Before
Sun et al. (2020)	China	Cross-sectional	Nursing	M: 15.2 F: 84.8	NR	474	NR	Anxiety	SAS	SAS ≥ 50	After
Syed et al. (2018)	Pakistan	Cross-sectional	Physiotherapy	M: 24.7 F: 75.3	Mean: 19.3	267	100	Anxiety Stress	DASS-42 (A) DASS-42 (S)	NR	Before
Thomas-Davis et al. (2020)	United States	Cross-sectional	Occupational therapy, Physiotherapy	M: 31 F: 69	Mean: 22.6 Range: 22–26	29	100	Stress	DASS-21 (S)	NR	Before
Thomas. (2022)	United States	Cross-sectional	Nursing	M: 11.3 F: 88.7	Mean: 21.9	267	21.4	Stress	SSI-R	NR	After
Tran et al. (2022)	Vietnam	Cross-sectional	Nursing	M: 6.9 F: 93.1	Mean: 20.5	1851	47.5	Anxiety	GAD-7	GAD-7 ≥ 8	After
Tuffah et al. (2021)	Iraq	Cross-sectional	Nursing	M: 31.2 F: 68.8	Mean: 21.55	237	13.1	Stress	ASIS	ASIS ≥ 69	After
Wilson et al. (2008)	United States	Cross-sectional	Social work	M: 11 F: 89	Mean: 32 Range: 21–61	162	NR	Anxiety	Anxiety scale	Anxiety scale ≥ 2	Before
Worku et al. (2019)	Ethiopia	Cross-sectional	Nursing, Midwifery, Public Health Officer, Pharmacy, Medical Laboratory, Anaesthesia	M: 57.6 F: 42.4	Mean: 20.9 Range: 18–30	384	100	Stress	PSS-14	PSS-14 ≥ 15	Before
Wynter et al. (2021)	Australia	Cross-sectional	Nursing, Midwifery	M: 6.1 F: 92.8	Mean: 23.56	638	22	Anxiety Stress	DASS-21 (A) DASS-21 (S)	DASS-21 (A) ≥ 4 DASS-21 (S) ≥ 4	After
Zakeri et al. (2021)	United States	Cross-sectional	Pharmacy	M: 32.8 F: 67.2	NR	238	63	Anxiety	CCAPS-62 (A)	CCAPS-62 (A) ≥ 1.22	After
Zeng et al. (2019)	China	Cross-sectional	Nursing	M: 2.6 F: 97.4	Mean: 20.2 Range: 17–24	544	89.9	Anxiety Stress	DASS-21 (A) DASS-21 (S)	NR	Before
Zhu et al. (2021)	China	Cross-sectional	Nursing	M: 13.2 F: 86.8	Mean: 20.72	342	100	Anxiety	GAD-7	GAD-7 ≥ 5	After
Zukhra et al. (2020)	Indonesia	Cross-sectional	Nursing	M: 9.3 F: 90.7	NR	247	100	Anxiety	ZSAS	NR	After
Nelson et al. (2018)	United States	Cohort	Nursing	Baseline: M: 13.5 F: 86.5 Follow-up: M: 12.5 F: 87.5	Mean: 28.8 (baseline) 30.5 (follow-up)	Baseline: 37 Follow-up: 33	79 (baseline)70 (follow-up	Anxiety Stress	GAD-7 PSS-10	GAD-7 ≥ 10 PSS-10 ≥ 14	Before
Newbury-Birch et al. (2002)	United Kingdom	Cohort	Dental	M: 34 F: 66	NR	Baseline: 47 Follow-up: 53	NR	Anxiety Stress	HADS (A) GHQ	HADS (A) ≥ 8 GHQ ≥ 5	Before
Shangraw et al. (2021)	Arizona	Cohort	Pharmacy	M: 38 F: 62	NR	Baseline: 304 Follow-up: 292	NR	Anxiety	GAD-7	GAD-7 ≥ 10	Before

NR = Not Reported, GAD-7 = Generalized Anxiety Disorder Scale, DASS-42 = Depression Anxiety Stress Scale 42-items, DASS-21 = Depression Anxiety Stress Scale 21-items, BAI = Beck’s Anxiety Inventory, GHQ-28 = Goldberg Health Questionnaire, STAI = State-Trait Anxiety Inventory, HADS = Hospital Anxiety and Depression Scale, STAIA = State-Trait Anxiety Inventory for Adults, PHQ-4 = Patient Health Questionnaire, ZSAS = Zung Self-Rating Anxiety Scale, SAS = Social Anxiety Scale, PHQ-9 = Patient Health Questionnaire, GADS = Goldberg abbreviated Anxiety and Depression Scale, PSS-4 = Perceived Stress Scale 4-items, PSS-10 = Perceived Stress Scale 10-items, ASNS = Assessment of Stress in Nursing Students, GHQ-12 = General Health Questionnaire, VMSI = Veterinary Medical Stressors Inventory, K10 = Kessler Psychological Distress Scale, SLSI = Student Life Stress Inventory, SSI-R = Gadzella’s Revised Student Life Stress Inventory, ASIS = Academic Stress Inventory Scale, SNSI = Student Nurse Stress Index, DES = Dental Environment Stress, CAS = COVID-19-associated Stress, CCAPS-62 = Counselling Center Assessment of Psychological Symptoms, SAAS = Scale for Assessing Academic Stress, CTAS-2 = Cognitive Test Anxiety Scale, MESSA = Modified Stress Scale for Adolescents

### Quality assessment

There were only observational studies that could be discovered, comprising 109 cross-sectional and three cohort studies. Table 2 presents the quality assessments of individual studies using the NOS rating. There were 51 (45.5%) high-quality (NOS > 6) and 61 (54.5%) low-quality (NOS ≤ 6) studies. For cross-sectional studies, 78 studies did not justify the sample size calculation, and 82 studies failed to take non-respondents into account. For cohort studies, all used written self-report and did not demonstrate an outcome of interest at the start of the study. Inter-rater reliability was tested using the results of quality appraisal by two independent reviewers (YXL and SESL). The Kappa statistic (*κ*) of 0.754 showed substantial agreement on quality assessment between reviewers.

**Table 2.**
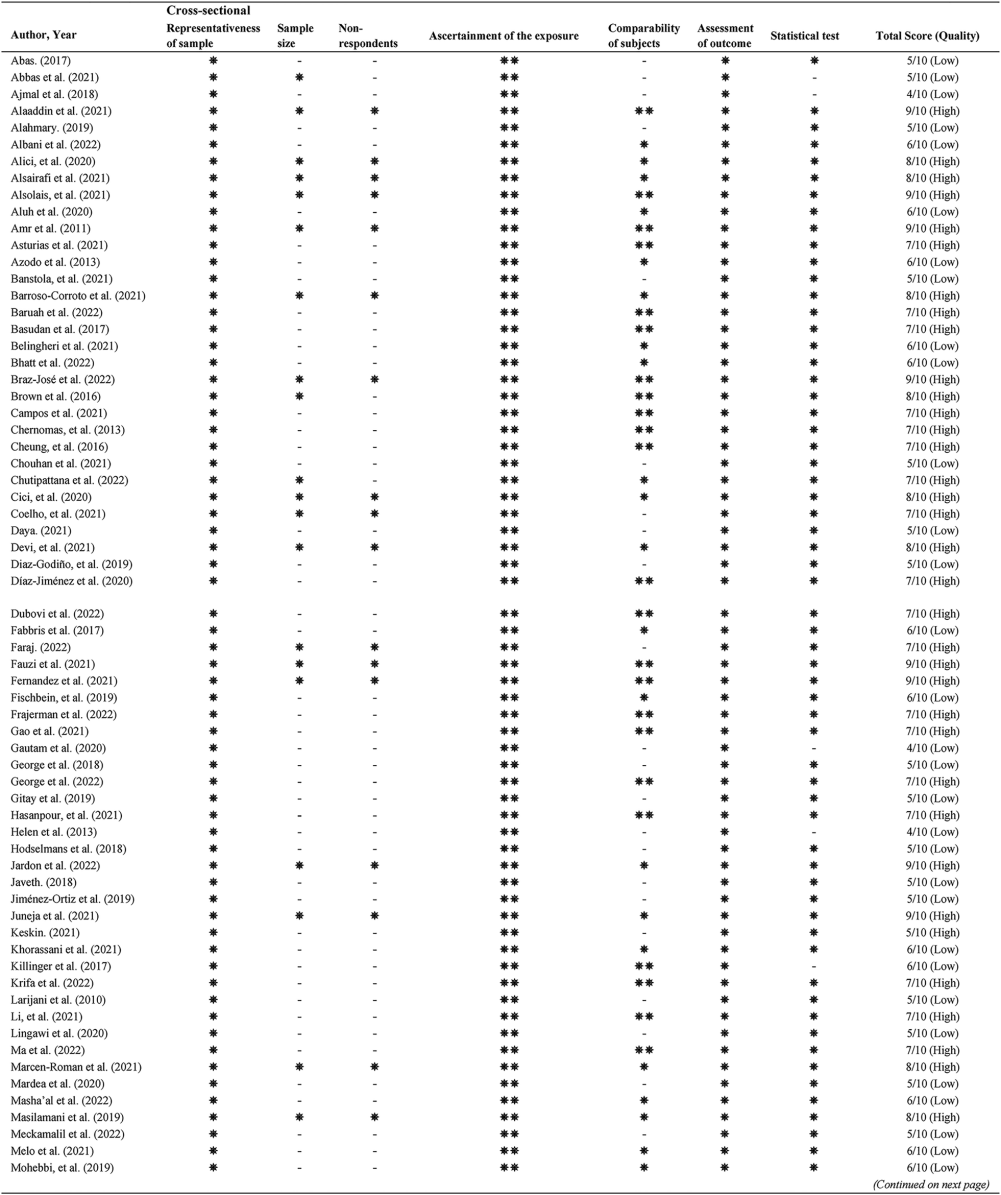

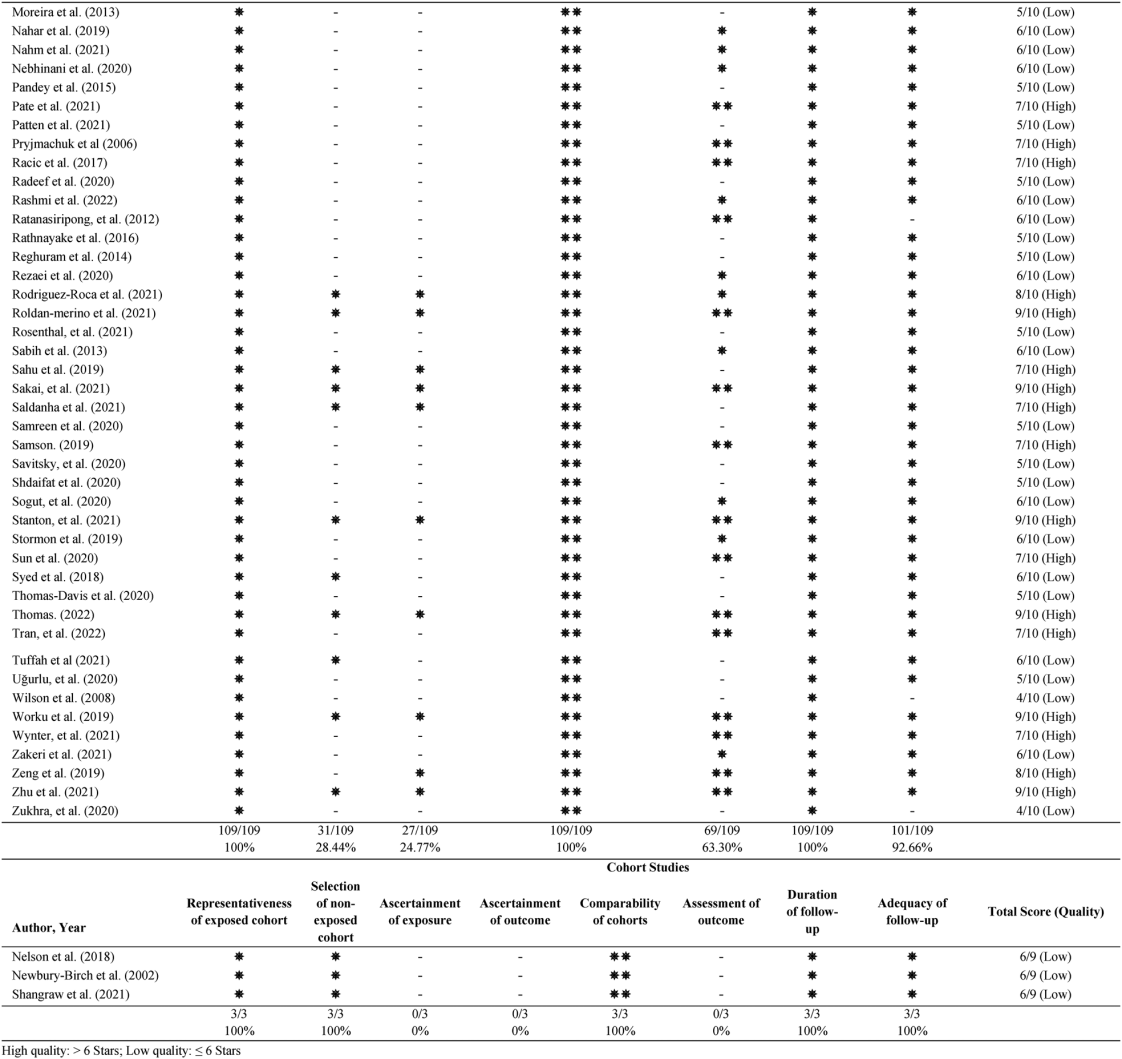
Newcastle-Ottawa Scale grading for quality assessment of the selected articles

### Global prevalence of anxiety stress symptoms among healthcare students

**Fig. 2:**
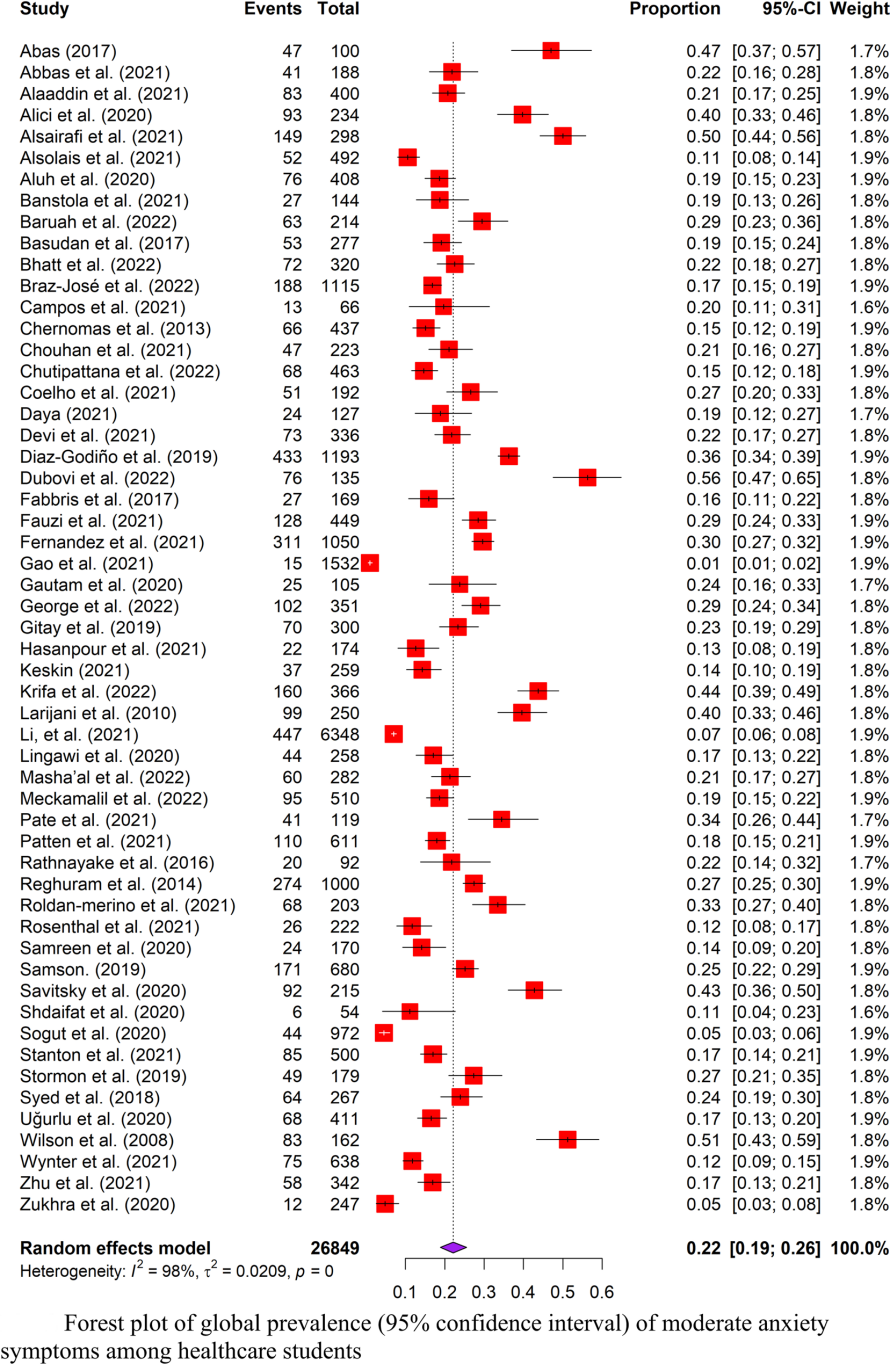
Forest plot of global prevalence (95% confidence interval) of moderate anxiety symptoms among healthcare students

**Fig. 3:**
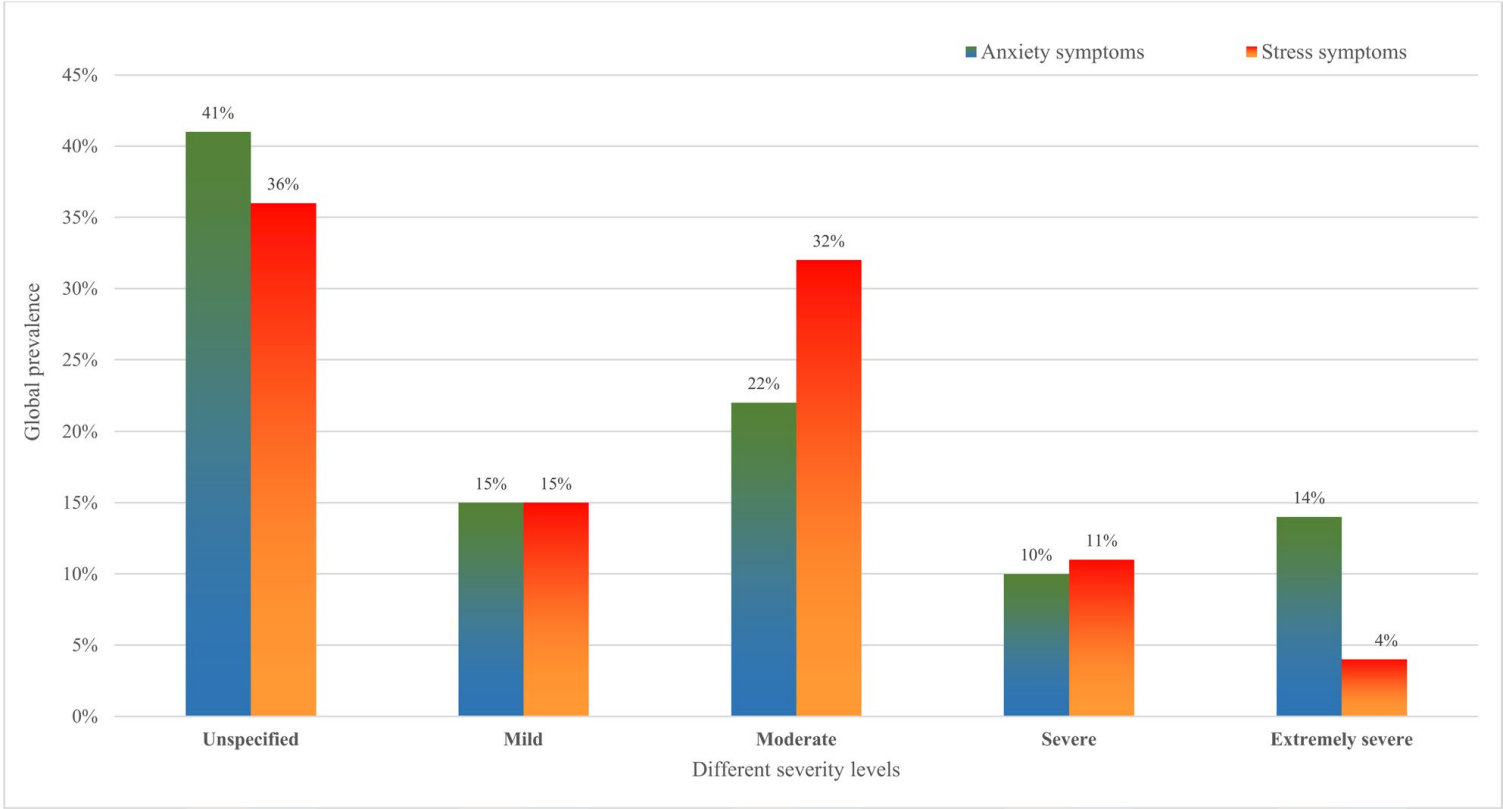
Bar chart of the global prevalence of different severity levels of anxiety and stress symptoms among healthcare students

The pooled global prevalence of different severity levels of anxiety symptoms among healthcare students was 41% (95% CI: 33–50), 15% (95% CI: 12–19), 22% (95% CI: 19–26), 10% (95% CI: 8–13), 14% (95% CI: 11–17) for unspecified (Fig. S1), mild (Fig. S2), moderate (Fig. 2), severe (Fig. S3), and extremely severe anxiety (Fig. S4), respectively. The pooled global prevalence of stress symptoms among healthcare students was 36% (95% CI: 25–47), 15% (95% CI: 12–18), 32% (95% CI: 25–40), 11% (95% CI: 8–15), 4% (95% CI: 2–5) for unspecified (Fig. S5), mild (Fig. S6), moderate (Fig. S7), severe (Fig. S8), and extremely severe stress (Fig. S9), respectively. Figure 3 summarises the global prevalence of different severity levels of anxiety and stress symptoms among healthcare students. Subgroup and meta-regression analyses were conducted to account for the considerable heterogeneity observed across all studies.

### Types of healthcare students

Table 3 summarises global and subgroup analyses of the prevalence of anxiety and stress symptoms among healthcare students. Fig. S10–S49 display the forest plots of subgroup analyses. A significant subgroup difference was found in the severe anxiety symptoms (*p* = 0.08) between nursing students (9%, 95% CI: 6–13) and non-nursing students (13%, 95% CI: 10–17). When comparing nursing and non-nursing students, the test for subgroup differences in the unspecific (*p* = 0.05), mild (*p* = 0.09), and moderate (*p* = 0.07) stress symptoms showed significant differences. Non-nursing students were found to have higher unspecific (48%, 95% CI:29–68) and mild (17%, 95% CI: 14–22) stress symptoms than nursing students, whereas nursing students (37%, 95% CI: 26–49) were found higher moderate stress symptoms than non-nursing students.

**Table 3:** Global and subgroup analyses of the prevalence of anxiety and stress symptoms among healthcare students

Anxiety		No.	Prevalence (95% CI)	*P*_subgroup_	Stress	No.	Prevalence (95% CI)	*P*_subgroup_
Global unspecified anxiety symptoms		28	41 (95% CI: 33, 50)	-	Global unspecified stress	15	36 (95% CI: 25, 47)	-
Subgroup analyses of the global unspecified anxiety					Subgroup analyses of global unspecified stress			
Types of students	Nursing	17	39 (95% CI: 28, 51)	0.60	Nursing	11	31 (95% CI: 19, 45)	0.05
	Non-Nursing	11	44 (95% CI: 30, 58)		Non-Nursing	4	48 (95% CI: 29, 68)	
Measurement instruments	DASS	5	50 (95% CI: 31, 70)	0.19	DASS	4	31 (95% CI: 12, 53)	0.46
	Non-DASS	23	39 (95% CI: 29, 49)		Non-DASS	11	38 (95% CI: 23, 53)	
Geographical region	Africa/Middle East	1	47 (95% CI: 41, 52)	0.06	Africa/Middle East	-	-	0.56
	America	8	40 (95% CI: 23, 58)		America	4	43 (95% CI: 24, 62)	
	Asia Pacific	12	33 (95% CI: 20, 48)		Asia Pacific	6	37 (95% CI: 17, 60)	
	Europe	7	57 (95% CI: 41, 72)		Europe	5	29 (95% CI: 6, 61)	
COVID Pandemic Period	Pre-COVID-19	16	41 (95% CI: 30, 52)	0.93	Pre-COVID-19	11	39 (95% CI: 27, 52)	0.31
	Post-COVID-19	12	42 (95% CI: 27, 57)		Post-COVID-19	4	27 (95% CI: 3, 63)	
Global mild anxiety		48	15 (95% CI: 12, 19)		Global mild stress	39	15 (95% CI: 12, 18)	
Subgroup analyses of global mild anxiety					Subgroup analyses of global mild stress			
Types of students	Nursing	28	17 (95% CI: 12, 23)	0.14	Nursing	19	12 (95% CI: 8, 17)	0.08
	Non-Nursing	20	13 (95% CI: 9, 17)		Non-Nursing	20	17 (95% CI: 14, 22)	
Measurement instruments	DASS	31	9 (95% CI: 8, 11)	< 0.001	DASS	31	15 (95% CI: 12, 18)	0.80
	Non-DASS	17	29 (95% CI: 23, 37)		Non-DASS	8	14 (95% CI: 4, 28)	
Geographical region	Africa/Middle East	3	13 (95% CI: 1, 35)	< 0.001	Africa/Middle East	4	16 (95% CI: 2, 39)	< 0.0001
	America	10	15 (95% CI: **8**, 23)		America	6	12 (95% CI: 7, 18)	
	Asia Pacific	31	17 (95% CI: 12, 22)		Asia Pacific	24	15 (95% CI: 10, 19)	
	Europe	1	7 (95% CI: 6, 9)		Europe	2	25 (95% CI: 3, 60)	
	Oceania	3	11 (95% CI: 3, 22)		Oceania	3	13 (95% CI: 9, 18)	
COVID Pandemic Period	Pre-COVID-19	26	16 (95% CI: 11, 21)	0.92	Pre-COVID-19	25	15 (95% CI: 11, 19)	0.83
	Post-COVID-19	22	15 (95% CI: 10, 21)		Post-COVID-19	14	14 (95% CI: 9, 20)	
Global moderate anxiety		55	22 (95% CI: 19, 26)		Global moderate stress	54	32 (95% CI: 25, 40)	
							*(Continued on next page)*	
Subgroup analyses of global moderate anxiety					Subgroup analyses of global moderate stress			
Types of students	Nursing	34	22 (95% CI: 17, 27)	0.59	Nursing	33	37 (95% CI: 26, 49)	0.07
	Non-Nursing	21	23 (95% CI: 19, 27)		Non-Nursing	21	25 (95% CI: 17, 34)	
Measurement instruments	DASS	31	20 (95% CI: 16, 23)	0.08	DASS	32	15 (95% CI: 12, 17)	< 0.0001
	Non-DASS	24	26 (95% CI: 19, 33)		Non-DASS	22	62 (95% CI: 52, 72)	
Geographical region	Africa/Middle East	3	27 (95% CI: 2, 65)	0.78	Africa/Middle East	5	35 (95% CI: 3, 77)	0.59
	America	11	24 (95% CI: 17, 33)		America	10	25 (95% CI: 12, 42)	
	Asia Pacific	36	21 (95% CI: 17, 26)		Asia Pacific	32	33 (95% CI: 23, 44)	
	Europe	2	24 (95% CI: 0, 100)		Europe	3	49 (95% CI: 3, 96)	
	Oceania	3	18 (95% CI: 3, 40)		Oceania	4	25 (95% CI: 0, 75)	
COVID Pandemic Period	Pre-COVID-19	28	25 (95% CI: 21, 28)	0.16	Pre-COVID-19	35	34 (95% CI: 25, 43)	0.55
	Post-COVID-19	27	20 (95% CI: 14, 26)		Post-COVID-19	19	29 (95% CI: 15, 44)	
Global severe anxiety		54	10 (95% CI: 8, 13)		Global severe stress	52	11 (95% CI: 8, 15)	
Subgroup analyses of severe anxiety					Subgroup analyses of severe stress			
Types of students	Nursing	33	9 (95% CI: 6, 13)	0.08	Nursing	32	12 (95% CI: 7, 18)	0.79
	Non-Nursing	21	13 (95% CI: 10, 17)		Non-Nursing	20	11 (95% CI: **7**, 15)	
Measurement instruments	DASS	31	11 (95% CI: 8, 14)	0.71	DASS	32	8 (95% CI: 6, 11)	0.06
	Non-DASS	23	10 (95% CI: 6, 15)		Non-DASS	20	17 (95% CI: 8, 28)	
Geographical region	Africa/Middle East	3	14 (95% CI: 0, 61)	0.82	Africa/Middle East	6	8 (95% CI: 0, 24)	0.94
	America	11	12 (95% CI: 8, 17)		America	8	12 (95% CI: 6, 19)	
	Asia Pacific	35	9 (95% CI: 6, 13)		Asia Pacific	32	12 (95% CI: 7, 18)	
	Europe	2	15 (95% CI: 0, 100)		Europe	3	9 (95% CI: 0, 45)	
	Oceania	3	11 (95% CI: 3, 22)		Oceania	3	10 (95% CI: 1, 26)	
COVID Pandemic Period	Pre-COVID-19	28	10 (95% CI: 8, 13)	1.00	Pre-COVID-19	33	13 (95% CI: 8, 19)	0.17
	Post-COVID-19	26	11 (95% CI: 6, 16)		Post-COVID-19	19	9 (95% CI: 5, 14)	
Global extremely severe anxiety		29	14 (95% CI: 11, 17)		Global extremely severe stress	29	4 (95% CI: 2, 5)	
							*(Continued on next page)*	
Subgroup analyses of extremely severe anxiety					Subgroup analyses of extremely severe stress			
Types of students	Nursing	15	12 (95% CI: 8, 16)	0.23	Nursing	15	4 (95% CI: 2, 7)	0.63
	Non-Nursing	14	16 (95% CI: 11, 22)		Non-Nursing	14	3 (95% CI: 1, 6)	
Measurement instruments	DASS	28	14 (95% CI: 11, 17)	0.66	DASS	28	4 (95% CI: 2, 5)	0.009
	Non-DASS	1	12 (95% CI: 8, 18)		Non-DASS	1	9 (95% CI: 5, 15)	
Geographical region	Africa/Middle East	3	12 (95% CI: 7, 17)	< 0.001	Africa/Middle East	3	1 (95% CI: 0, 9)	< 0.0001
	America	6	7 (95% CI: 5, 9)		America	6	2 (95% CI: 1, 4)	
	Asia Pacific	17	16 (95% CI: 11, 21)		Asia Pacific	17	4 (95% CI: 2, 7)	
	Europe	1	26 (95% CI: 24, 29)		Europe	1	12 (95% CI: 10, 14)	
	Oceania	2	17 (95% CI: 9, 27)		Oceania	2	8 (95% CI: 0, 39)	
COVID Pandemic Period	Pre-COVID-19	18	15 (95% CI: 11, 19)	0.30	Pre-COVID-19	18	4 (95% CI: 2, 6)	0.85
	Post-COVID-19	11	12 (95% CI: 6,18)		Post-COVID-19	11	4 (95% CI: 1, 7)	

No. = number, CI = Confidence interval, *p < 0.05; **p < 0.01; ***p < 0.001. DASS = Depression, Anxiety and Stress Scale; COVID = coronavirus disease 2019

### Types of measurement

Notably, a series of subgroup analyses revealed that healthcare students experienced significantly (*p* < 0.1) lower prevalences of mild (9%, 95% CI: 8–11) and moderate (20%, 95% CI: 16–23) anxiety symptoms when the DASS-21 was used as the measurement tool than other measurements. The DASS-21 also showed that moderate stress (15%, 95% CI: 12–17), severe stress (8%, 95% CI: 6–11), and extremely severe stress (4%, 95% CI: 2–5) symptoms were significantly less common than with other measures.

### Geographical region

Healthcare students had significantly (*p* < 0.1) higher prevalences of unspecific anxiety symptoms (57%, 95% CI: 41–72), extremely severe anxiety symptoms (26%, 95% CI: 24–29), mild stress symptoms (25%, 95% CI: 3–60), and extremely severe stress symptoms (12%, 95% CI: 10–14) in Europe than other regions. However, healthcare students had a significantly higher prevalence of mild anxiety symptoms in Asia Pacific (17%, 95% CI: 12–22) when compared to other regions. However, only one to three studies were in counterparts, and the interpretation of this result was cautious.

### COVID-19 pandemic period

The global prevalence of different levels of anxiety and stress symptoms was similar before and after the COVID-19 pandemic. Studies conducted before the COVID-19 pandemic had a prevalence of 10%–41% (95% CI: 8–30, 13–52), while those conducted after the COVID-19 pandemic had a prevalence of 11%–42% (95% CI: 6–27, 16–57).

### Meta-regression

A series of meta-regression analyses were conducted for different levels of anxiety and stress symptoms against sample size, study quality (NOS), and year of publication. The sample size was a significant covariate in the global prevalence estimates of moderate anxiety symptoms (*β* < −0.001, *p* = 0.02), severe anxiety symptoms (*β* < −0.001, *p* = 0.04), and moderate stress symptoms (*β* < −0.001, *p* = 0.01). These results indicated that lower prevalence estimates of moderate anxiety symptoms, severe anxiety symptoms, and moderate stress symptoms were found in the studies with a smaller sample size when compared to a larger sample size. Study quality was a significant covariate in the global prevalence estimates of severe anxiety symptoms (*β* = 0.027, *p* = 0.04), indicating the studies with higher quality have a higher prevalence estimate of severe anxiety symptoms. However, the year of publication did not have effects on the different levels of anxiety and stress symptoms.

### Publication bias and certainty of evidence

To investigate publication bias, funnel plots and Egger’s test were used (Fig. S50–S59). Upon visual inspection, the prevalence of unspecified, mild, moderate, and severe anxiety symptoms and moderate stress symptoms were shown as asymmetrical funnel plots. The prevalence of unspecific anxiety (Fig. S50, *p* = 0.01), moderate anxiety (Fig. S52, *p* < 0.001), severe anxiety symptoms (Fig. S53, *p* < 0.001), and moderate stress symptoms (Fig. S57, *p* = 0.004), respectively, had *p*-values of less than 0.05 according to Egger’s test. These findings suggested that there could be publishing biases. Based on the GRADE criteria, the certainty of evidence was very low for different levels of prevalence estimates (Table S5), and the domain of inconsistency was downgraded because all meta-analyses had sustainable heterogeneities between studies and small sample sizes.

## Discussion

### Summary of findings

This systematic review comprised 112 studies totalling 42,331 healthcare students from 20 healthcare courses in 43 countries. The severity of anxiety and stress symptoms was classified as unspecified, mild, moderate, severe, and extremely severe (Fig. 3). The pooled global prevalence of unspecified, mild, moderate, severe, and extremely severe anxiety symptoms was 41%, 15%, 22%, 10%, and 14%, respectively. The pooled global prevalence of unspecified, mild, moderate, severe, and extremely severe stress symptoms was 36%, 15%, 32%, 11%, and 4%. A series of subgroup analyses revealed that types of healthcare students, geographical regions, and types of measurement were all significant factors influencing certain levels of anxiety and stress symptoms. The sample size and study quality were significant covariates on prevalence estimates of moderate anxiety, severe anxiety, and moderate stress symptoms. Although 45.5% of studies were rated as high quality based on NOS, the certainty of evidence was very low for all levels of anxiety and stress symptoms according to the GRADE criteria.

#### Global prevalence of different levels of anxiety symptoms in healthcare students

When comparing the different severity levels of anxiety symptoms, unspecific anxiety symptoms (41%) had the highest prevalence, followed by moderate (22%), mild (15%), extremely severe (14%) and severe (10%) anxiety symptoms (Fig. 3). Our results were consistent with a previous review among nursing students [16], which reported moderate anxiety being most prevalent (25.1%) and followed by mild anxiety (19.4%) and severe anxiety (15.1%). Some discrepancies were found might be due to different classifications of symptoms, and it may miss out on extremely severe anxiety symptoms between the two reviews. Our review found that the prevalence of unspecific anxiety symptoms was 41%. As all different severities of anxiety symptoms were combined into a single category, this may explain the higher prevalence. Disruptive symptoms of moderate anxiety might allow students to identify their adverse mental condition, thus seeking professional help and decreasing severe and extremely severe anxiety rates markedly. However, all studies in these categories were cross-sectional studies, whereby data was only collected at one time point [152]. This may cause fluctuations in anxiety levels depending on the period during which the studies were done. Many studies reporting anxiety were performed before the start of COVID-19 when healthcare education and clinical attachments were still in full force; thus, this may also have led to higher rates of moderate anxiety levels.

### Global prevalence of different levels of stress symptoms among healthcare students

According to our review, unspecified stress symptoms (36%) were the most prevalent type of stress symptom among healthcare students, followed by moderate (32%), mild (15%), severe (11%), and extremely severe (4%) stress symptom levels. The highest prevalence of unspecified stress symptoms can be explained by combining all the severity of stress symptoms into a single group. The pattern of the global prevalence of different levels of stress symptoms was different when we compared the results of a previous systematic review [16]: moderate stress symptoms predominated (42.1%), with severe (19.5%) and mild (16.7%) in the second and third, respectively. This can be explained by the possibility of different populations; our reviews included different types of healthcare students, whereas the previous students focused on nursing students. Healthcare students frequently face stress because of clinical training and academic sources [15]. However, it has been found that mild to moderate stress among healthcare students has positive benefits on working memory and encourages them to perform better [153, 154]. The much lower rates of severe and extremely severe stress may be attributable to enhanced emotional support from instructors in recent years, as well as expanded access to stress management programs and activities for healthcare students on campus [15].

#### Types of healthcare students

Our subgroup analyses showed that the global prevalence of anxiety and stress symptoms in non-nursing students was higher at 13%−44% and 3%−48%, respectively, as compared to nursing students, with a prevalence of 9%−39% and 4%−37%. This result is similar to the findings by Q Miao, L Xie, B Xing, X Wang, S Tang and H Luo [155], which found a difference between anxiety levels between nursing (15.3%) and non-nursing (18.9%) students. The lower prevalence of anxiety and stress symptoms in nursing students may be due to the nursing curriculum, which includes modules on psychology and mental health [155]. Thus, providing students with the ability to better manage their anxiety and stress levels. The nature of nursing profession might also have taught students to be more resilient as compared to their other healthcare counterparts [156]. Nursing students during the COVID-19 pandemic who had been through infection control courses in school would also be able to adapt easily to the ever-changing infection control practices. This would allow future nurses to be less anxious and stressed about changes that the pandemic may bring about [157]. This may allow nursing students to be able to overcome stressful situations easily and adapt positively, thus lowering anxiety and stress levels [158].

#### Use of different measurements

A series of subgroup analyses showed that a lower prevalence of anxiety and stress symptoms was found when using the DASS-21 as a measurement tool [151]. It seems that the different measurement tools could affect the findings. The anxiety domain of the DASS-21 measures the symptoms of worry, irritation, restlessness, and panic [151]. For other scales, such as the GAD-7 [159], it evaluates signs of concern and irritation. Since the different signs and symptoms included different tools, different prevalences may have resulted. Our review observed that the Perceived Stress Scale (PSS) was the most common tool for measuring stress symptoms [160]. PSS primarily measures two characteristics, namely: perceived helplessness and perceived self-efficacy [160], whereas the stress domain of the DASS-21 evaluates feelings of anxious arousal, annoyance, and impatience [151]. Healthcare students may feel helpless since tertiary education places a strong emphasis on self-discipline [161]. This may have resulted in a different prevalence using different tools.

#### Geographical region

Subgroup analyses showed that healthcare students in the European region reported a higher prevalence of stress and anxiety symptoms than in other regions, especially on unspecific anxiety symptoms, extremely severe anxiety symptoms, mild stress symptoms, and extremely severe stress symptoms. This could be attributed to the Spanish studies, which comprised 5 of the 13 studies in this category. Spain has an unemployment rate of 8.6% among those with tertiary education, compared to 6.18% globally [162]. The foreseeable likelihood of unemployment following graduation may have contributed to the increased anxiety and stress. In addition, the COVID-19 regulations, such as the duration of lockdowns in various nations and regions, may have had an impact on the prevalence estimations [163]. We also note that the unbalanced subgroups may have caused inaccurate estimation of effect sizes [36]. As there are only 1–3 studies from certain regions in the subgroup analyses, this may affect the accuracy of this result.

### COVID-19 pandemic period

Due to unforeseen disturbances to the educational system, it was anticipated that the commencement of COVID-19 would increase the prevalence of anxiety and stress in healthcare students [164]. However, this analysis discovered no significant subgroup differences in the prevalence of anxiety. This finding suggests that healthcare educators should address the anxiety and stress phenomena of healthcare students, regardless of the COVID-19 circumstance. Notably, we found that healthcare students experienced more anxiety and stress symptoms at certain levels before the COVID-19 pandemic. These findings are in line with a previous review by M Mulyadi, SI Tonapa, S Luneto, WT Lin and BO Lee [18]. We speculate that this might be due to healthcare students knowing more about the pandemic and being able to use what they have learned to control and lessen anxiety and stress levels [1]. Given that online learning has been used in healthcare education around the world since pre-COVID-19 in addition to traditional face-to-face learning [165], students were able to quickly switch to online sessions when COVID-19 prevented the possibility of physical classes. After COVID-19 was declared a global pandemic, healthcare students had reduced clinical placements [166]. Hence reducing the anxiety and stress levels of healthcare students.

### Study quality and sample size

According to the results of the meta-regression, the study quality and sample size had a substantial effect on the global prevalence estimates of moderate anxiety, severe anxiety, and moderate stress symptoms. Our review indicates that high-quality studies found a higher prevalence of severe anxiety symptoms in healthcare students. We observed that the main reasons for a good-quality study with reliable control of confounding factors will provide more precise and accurate results [167]. Given that this review has four levels of anxiety symptoms, it could be explained by this result. Furthermore, our results suggest a higher prevalence of moderate anxiety and stress symptoms in studies with a smaller sample size. One explanation could be a small sample size with low statistical power, which could overestimate the effect size [168].

## Strengths and limitations

To the best of our knowledge, this is the first to compute the global prevalence of different levels of stress and anxiety symptoms in healthcare students including nursing, pharmacy, and allied health students. A thorough approach was employed, comprising a three-step search strategy that was carried out in ten databases along with grey literature. The results are more broadly applicable because 42,336 healthcare students were included in a total of 112 studies from five different regions. A comprehensive review of quality was also done at both the individual and overall levels. We adopted the Hartung-Knapp-Sidik-Jonkman (HKSJ) method, which was known to perform better by showing consistent results with adequate error rates than other methods [33]. The Freeman-Tukey double arcsine transformation was used to stabilise the variance of each study’s proportion in two-step meta-analysis methods [34]. By performing subgroup and meta-regression analyses, heterogeneity was investigated, and significant factors that influenced the prevalence estimates of different levels of anxiety and stress symptoms were explored.

Nevertheless, several limitations should be acknowledged. First, all studies were written and published in English, so the generalisability of the results was constrained. Second, many of the included papers were from Asia and America, which may have had an impact on how prevalent anxiety and stress symptoms were among healthcare students. Third, a less accurate prevalence could be the consequence of multiple studies using small sample numbers and various assessment tools. Fourth, a self-reported screening instrument was used to compute the prevalence instead of a diagnostic interview, which might lead to a bias towards social desirability. Fifth, the cut-off points utilised to characterise symptoms of stress and anxiety were different, which might have an impact on the prevalence estimates. Due to the significant heterogeneity of pooled prevalence estimates, which may indicate data variability, the results should be carefully evaluated. Lastly, this review showed very low certainty of the evidence, which diminished the internal validity of the results.

### Future research direction

Future studies can investigate relevant strategies that could help reduce anxiety and stress symptoms in healthcare students. Although we performed a series of subgroup and meta-regression analyses, the reasons of substantial heterogeneity were unexplained. Future research may examine additional factors that might have an impact on healthcare students’ anxiety and stress symptoms. We adopted the NOS to assess the quality of cross-sectional studies, but few of them justified their sample sizes and provided information on non-respondents. Sample size justification is essential to prevent under- or overestimating the sample size in research [168], and response bias might arise from incomplete data on non-respondents [169]. Future studies should adhere to the checklist for Strengthening the Reporting of Observational Studies in Epidemiology [170], particularly the explanation of sample size calculation, and non-response rate. Future cohort studies should adequately consider the ascertainment and assessment of outcomes while carrying out the study.

### Implications for clinical practice and healthcare policy

This review provided new knowledge on the different levels of anxiety and stress symptoms, and these findings alerted leaders of healthcare education to improve the awareness of the severity of problems among healthcare students. To benefit healthcare students, early detection and preventive interventions such as cognitive behavioural therapy and eHealth literacy [171] should be given a higher priority. Given that our subgroup analyses showed that non-nursing students in the European region experience higher prevalence estimates than their counterparts, targeted interventions should be developed for these high-risk groups. Our results demonstrate that different instruments employed can change prevalence estimates, indicating the need for standardised assessment methods in future.

## Conclusion

Based on this review, healthcare students around the world frequently experience different levels of stress and anxiety symptoms. The meta-analysis has helped to advance knowledge of the potential effects of various healthcare student types, geographical regions, different instruments, sample sizes, and study quality on the prevalence of anxiety and stress symptoms. These findings add value to healthcare educators’ current knowledge by emphasizing the need for timely interventions to address the requirements of healthcare students globally and stressing the need to prioritise the psychological well-being of healthcare students.

## Supplementary Information

Supplementary Material 1

## Data Availability

No datasets were generated or analysed during the current study.
