# Supplementary material for: Global prevalence of different levels of anxiety and stress symptoms in healthcare students: A meta-analysis and meta-regression
Source: Ann Gen Psychiatry. 2026 Jan 3;25:4. doi: 10.1186/s12991-025-00618-1 (PMC12870317; doi:10.1186/s12991-025-00618-1)
Supplement: Supplementary file 1 — Supplementary Material 1 [file 12991_2025_618_MOESM1_ESM.docx]

**Table S1** PRISMA checklist

| **Section and Topic** | **Item #** | **Checklist item** | **Location where item is reported** |
| --- | --- | --- | --- |
| **TITLE** | | |  |
| Title | 1 | Identify the report as a systematic review. | P1 |
| **ABSTRACT** | | |  |
| Abstract | 2 | See the PRISMA 2020 for Abstracts checklist. | P3 |
| **INTRODUCTION** | | |  |
| Rationale | 3 | Describe the rationale for the review in the context of existing knowledge. | P4-5 |
| Objectives | 4 | Provide an explicit statement of the objective(s) or question(s) the review addresses. | P5 |
| **METHODS** | | |  |
| Eligibility criteria | 5 | Specify the inclusion and exclusion criteria for the review and how studies were grouped for the syntheses. | P5-6 |
| Information sources | 6 | Specify all databases, registers, websites, organisations, reference lists and other sources searched or consulted to identify studies. Specify the date when each source was last searched or consulted. | P6 |
| Search strategy | 7 | Present the full search strategies for all databases, registers and websites, including any filters and limits used. | P6 |
| Selection process | 8 | Specify the methods used to decide whether a study met the inclusion criteria of the review, including how many reviewers screened each record and each report retrieved, whether they worked independently, and if applicable, details of automation tools used in the process. | P6-7 |
| Data collection process | 9 | Specify the methods used to collect data from reports, including how many reviewers collected data from each report, whether they worked independently, any processes for obtaining or confirming data from study investigators, and if applicable, details of automation tools used in the process. | P7 |
| Data items | 10a | List and define all outcomes for which data were sought. Specify whether all results that were compatible with each outcome domain in each study were sought (e.g. for all measures, time points, analyses), and if not, the methods used to decide which results to collect. | P7 |
|  | 10b | List and define all other variables for which data were sought (e.g. participant and intervention characteristics, funding sources). Describe any assumptions made about any missing or unclear information. | P7 |
| Study risk of bias assessment | 11 | Specify the methods used to assess risk of bias in the included studies, including details of the tool(s) used, how many reviewers assessed each study and whether they worked independently, and if applicable, details of automation tools used in the process. | P7 |
| Effect measures | 12 | Specify for each outcome the effect measure(s) (e.g. risk ratio, mean difference) used in the synthesis or presentation of results. | P8 |
| Synthesis methods | 13a | Describe the processes used to decide which studies were eligible for each synthesis (e.g. tabulating the study intervention characteristics and comparing against the planned groups for each synthesis (item #5)). | P8 |
|  | 13b | Describe any methods required to prepare the data for presentation or synthesis, such as handling of missing summary statistics, or data conversions. | P7-8 |
|  | 13c | Describe any methods used to tabulate or visually display results of individual studies and syntheses. | P8 |
|  | 13d | Describe any methods used to synthesize results and provide a rationale for the choice(s). If meta-analysis was performed, describe the model(s), method(s) to identify the presence and extent of statistical heterogeneity, and software package(s) used. | P8 |
|  | 13e | Describe any methods used to explore possible causes of heterogeneity among study results (e.g. subgroup analysis, meta-regression). | P8 |
|  | 13f | Describe any sensitivity analyses conducted to assess robustness of the synthesized results. | P8 |
| Reporting bias assessment | 14 | Describe any methods used to assess risk of bias due to missing results in a synthesis (arising from reporting biases). | P8 |
| Certainty assessment | 15 | Describe any methods used to assess certainty (or confidence) in the body of evidence for an outcome. | P8 |
| **RESULTS** | | |  |
| Study selection | 16a | Describe the results of the search and selection process, from the number of records identified in the search to the number of studies included in the review, ideally using a flow diagram. | P9 |
|  | 16b | Cite studies that might appear to meet the inclusion criteria, but which were excluded, and explain why they were excluded. | 9 |
| Study characteristics | 17 | Cite each included study and present its characteristics. | P10-11 |
| Risk of bias in studies | 18 | Present assessments of risk of bias for each included study. | P10 |
| Results of individual studies | 19 | For all outcomes, present, for each study: (a) summary statistics for each group (where appropriate) and (b) an effect estimate and its precision (e.g. confidence/credible interval), ideally using structured tables or plots. | P11-12 |
| Results of syntheses | 20a | For each synthesis, briefly summarise the characteristics and risk of bias among contributing studies. | P11-12 |
|  | 20b | Present results of all statistical syntheses conducted. If meta-analysis was done, present for each the summary estimate and its precision (e.g. confidence/credible interval) and measures of statistical heterogeneity. If comparing groups, describe the direction of the effect. | P11-12 |
|  | 20c | Present results of all investigations of possible causes of heterogeneity among study results. | P11-12 |
|  | 20d | Present results of all sensitivity analyses conducted to assess the robustness of the synthesized results. | P11-12 |
| Reporting biases | 21 | Present assessments of risk of bias due to missing results (arising from reporting biases) for each synthesis assessed. | P12-13 |
| Certainty of evidence | 22 | Present assessments of certainty (or confidence) in the body of evidence for each outcome assessed. | P12-13 |
| **DISCUSSION** | | |  |
| Discussion | 23a | Provide a general interpretation of the results in the context of other evidence. | P13-18 |
|  | 23b | Discuss any limitations of the evidence included in the review. | P18-19 |
|  | 23c | Discuss any limitations of the review processes used. | P18-19 |
|  | 23d | Discuss implications of the results for practice, policy, and future research. | P19-21 |
| **OTHER INFORMATION** | | |  |
| Registration and protocol | 24a | Provide registration information for the review, including register name and registration number, or state that the review was not registered. | P5 |
|  | 24b | Indicate where the review protocol can be accessed, or state that a protocol was not prepared. | P5 |
|  | 24c | Describe and explain any amendments to information provided at registration or in the protocol. | - |
| Support | 25 | Describe sources of financial or non-financial support for the review, and the role of the funders or sponsors in the review. | P20-21 |
| Competing interests | 26 | Declare any competing interests of review authors. | P21 |
| Availability of data, code and other materials | 27 | Report which of the following are publicly available and where they can be found: template data collection forms; data extracted from included studies; data used for all analyses; analytic code; any other materials used in the review. | References, Appendix |

*From:*  Page MJ, McKenzie JE, Bossuyt PM, Boutron I, Hoffmann TC, Mulrow CD, et al. The PRISMA 2020 statement: an updated guideline for reporting systematic reviews. BMJ 2021;372:n71. doi: 10.1136/bmj.n71

**Table S2** Eligibility criteria

| **Criteria** | **Inclusion** | **Exclusion** |
| --- | --- | --- |
| Population | - Students from nursing, pharmacy, and any other allied-health-related courses | - Students from medical and non-healthcare-related courses |
| Condition | - Prevalence of anxiety or stress, with anxiety or stress measured and defined with validated and reliable tools of measurement and cut-off | - Studies that do not report any measurement of anxiety, depression or stress using a measurement tool |
| Context | - Any setting |  |
| Study design | - Observational studies, such as cohort, case-control, and cross-sectional studies - Experimental studies that provide prevalence rates of anxiety or stress |  |
| Year of publication | - No limit |  |
| Publication type | - Published studies - Unpublished Dissertations and Theses | - Conference papers - Book chapter reviews - Letters - Editorials - Discussion papers - Study protocols - Studies with solely abstract |
| Language | - English | - Non-English |

**Table S3** Search strategy

| **Database** | **ID** | **Search** | **Results** |
| --- | --- | --- | --- |
| PubMed | Concept 1: Anxiety OR Stress | |  |
|  | #1 | **"Anxiety"[Mesh]** | 105,864 |
|  | #2 | Nervous*[Title/Abstract] OR Anxious*[Title/Abstract] OR Anxiet*[Title/Abstract] OR "Social Anxiety"[Title/Abstract] OR Angst[Title/Abstract] OR Hypervigilance[Title/Abstract] OR "Anticipatory anxiety"[Title/Abstract] | 604,372 |
|  | #3 | **"Stress, Psychological"[Mesh]** | 149,228 |
|  | #4 | **Stress*[Title/Abstract] OR "Psychologic Stress*"[Title/Abstract] OR "Psychological Stress*"[Title/Abstract] OR "Life Stress*"[Title/Abstract] OR "Mental Stress*"[Title/Abstract] OR "Acute Stress*"[Title/Abstract] OR "Chronic Stress*"[Title/Abstract]** | 1,029,649 |
|  | #5 | #1 OR #2 OR #3 OR #4 | 1,634,687 |
|  | Concept 2: Healthcare students | |  |
|  | #6 | **((("Students, Dental"[Mesh]) OR "Students, Nursing"[Mesh]) OR "Students, Pharmacy"[Mesh]) OR "Students, Public Health"[Mesh]** | 40,324 |
|  | #7 | **Health occupations student[Title/Abstract] OR Healthcare student[Title/Abstract] OR Allied health student[Title/Abstract] OR Nursing student[Title/Abstract] OR Physiotherapy student[Title/Abstract] OR Occupational therapy student[Title/Abstract] OR Dentistry student[Title/Abstract] OR Veterinary science student[Title/Abstract] OR Pharmacy student[Title/Abstract] OR Psychology student[Title/Abstract] OR Public health student[Title/Abstract] OR Social work student[Title/Abstract] OR Speech therapy student[Title/Abstract] OR Radiology student[Title/Abstract] OR Dietician student[Title/Abstract] OR Podiatry student[Title/Abstract]** | 10,110 |
|  | #8 | #6 OR #7 | 48,025 |
|  | Merging Concepts: (Anxiety OR Stress) AND Healthcare Students | |  |
|  | #9 | #5 AND #8 | 3,299 |
|  |  |  |  |
| Cochrane | Concept 1: Anxiety OR Stress | |  |
|  | #1 | MeSH descriptor: [Anxiety] explode all trees | 9,277 |
|  | #2 | (anxiety OR nervousness OR anxiousness OR "social anxiety" OR angst OR hypervigilance OR "anticipatory anxiety"):ti,ab,kw | 64,421 |
|  | #3 | MeSH descriptor: [Stress, Psychological] explode all trees | 6,894 |
|  | #4 | ("Psychological Stress" OR "acute stress" OR "chronic stress" OR "mental stress" OR "physically induced stress" OR "surgical stress" OR "Life Stresses" OR "Life Stress" OR "Life stressor" OR "Psychologic Stress"):ti,ab,kw | 5,046 |
|  | #5 | #1 OR #2 OR #3 OR #4 | 72,011 |
|  | Concept 3: Healthcare students | |  |
|  | #6 | MeSH descriptor: [Students, Dental] explode all trees OR MeSH descriptor: [Students, Nursing] explode all trees OR MeSH descriptor: [Students, Pharmacy] explode all trees OR MeSH descriptor: [Students, Public Health] explode all trees | 780 |
|  | #7 | (“Health occupations student” OR “Healthcare student” OR “Allied health student” OR “Nursing student” OR “Physiotherapy student” OR “Occupational therapy student” OR “Dentistry student” OR “Veterinary science student” OR “Pharmacy student” OR “Psychology student” OR “Public health student” OR “Social work student” OR “Speech therapy student” OR “Radiology student” OR “Dietician student” OR “Podiatry student”):ti,ab,kw | 801 |
|  | #8 | #6 OR #7 | 1,385 |
|  | Merging Concepts: (Anxiety OR Stress) AND Healthcare Students | |  |
|  | #8 | #5 AND #8 | 169 |
|  |  |  |  |
| EMBASE.com | Concept 1: Anxiety OR Stress | |  |
|  |  | |  |
|  | #1 | 'anxiety'/exp | 266,194 |
|  | #2 | nervous*:ab,ti OR anxious*:ab,ti OR anxiet*:ab,ti OR 'social anxiety':ab,ti OR angst:ab,ti OR hypervigilance:ab,ti OR 'anticipatory anxiety':ab,ti | 777,248 |
|  | #3 | 'mental stress'/exp | 189,358 |
|  | #4 | stress*:ab,ti OR 'psychologic stress*':ab,ti OR 'psychological stress*':ab,ti OR 'life stress*':ab,ti OR 'mental stress*':ab,ti OR 'acute stress*':ab,ti OR 'chronic stress*':ab,ti | 1,250,986 |
|  | #5 | #1 OR #2 OR #3 OR #4 | 2,073,684 |
|  | Concept 3: Healthcare students | |  |
|  | #6 | 'paramedical student'/exp OR 'public health student'/exp OR 'social work student'/exp OR 'veterinary student'/exp | 51,730 |
|  | #7 | 'health occupations student':ab,ti OR 'healthcare student':ab,ti OR 'allied health student':ab,ti OR 'nursing student':ab,ti OR 'physiotherapy student':ab,ti OR 'occupational therapy student':ab,ti OR 'dentistry student':ab,ti OR 'veterinary science student':ab,ti OR 'pharmacy student':ab,ti OR 'psychology student':ab,ti OR 'public health student':ab,ti OR 'social work student':ab,ti OR 'speech therapy student':ab,ti OR 'radiology student':ab,ti OR 'dietician student':ab,ti OR 'podiatry student':ab,ti | 3,470 |
|  | #8 | #6 OR #7 | 52,747 |
|  | Merging Concepts: Anxiety OR Stress | |  |
|  |  |  |  |
|  | Merging Concepts: (Anxiety OR Stress) AND Healthcare Students | |  |
|  | #9 | #5 AND #8 | 4,138 |
|  |  |  |  |
| CINAHL | Concept 1: Anxiety | |  |
|  | #1 | (MH "Anxiety") | 55,245 |
|  | #2 | TI ( Nervous* OR Anxious* OR Anxiet* OR “Social Anxiety” OR Angst OR Hypervigilance OR “Anticipatory anxiety” ) OR AB ( Nervous* OR Anxious* OR Anxiet* OR “Social Anxiety” OR Angst OR Hypervigilance OR “Anticipatory anxiety” ) | 137,470 |
|  | #3 | (MH "Stress") | 11,335 |
|  | #4 | TI ( Stress* OR “Psychologic Stress*” OR “Psychological Stress*” OR “Life Stress*” OR “Mental Stress*” OR “Acute Stress*” OR “Chronic Stress*” ) OR AB ( Stress* OR “Psychologic Stress*” OR “Psychological Stress*” OR “Life Stress*” OR “Mental Stress*” OR “Acute Stress*” OR “Chronic Stress*” ) | 192,322 |
|  | #5 | #1 OR #2 OR #3 OR #4 | 325,115 |
|  | Concept 3: Healthcare students | |  |
|  | #6 | (MH "Students, Allied Health+") OR (MH "Students, Chiropractic") OR (MH "Students, Dental") OR (MH "Students, Midwifery") OR (MH "Students, Nursing+") OR (MH "Students, Nursing, Practical") OR (MH "Students, Pharmacy") OR (MH "Students, Podiatry") OR (MH "Students, Pre-Nursing") | 62,950 |
|  | #7 | TI ( “Health occupations student” OR “Healthcare student” OR “Allied health student” OR “Nursing student” OR “Physiotherapy student” OR “Occupational therapy student” OR “Dentistry student” OR “Veterinary science student” OR “Pharmacy student” OR “Psychology student” OR “Public health student” OR “Social work student” OR “Speech therapy student” OR “Radiology student” OR “Dietician student” OR “Podiatry student” ) OR AB ( “Health occupations student” OR “Healthcare student” OR “Allied health student” OR “Nursing student” OR “Physiotherapy student” OR “Occupational therapy student” OR “Dentistry student” OR “Veterinary science student” OR “Pharmacy student” OR “Psychology student” OR “Public health student” OR “Social work student” OR “Speech therapy student” OR “Radiology student” OR “Dietician student” OR “Podiatry student” ) | 3,634 |
|  | #8 | #6 OR #7 | 64,128 |
|  | Merging Concepts: (Anxiety OR Stress) AND Healthcare Students | |  |
|  | #9 | #5 AND #8 | 3,736 |
|  |  |  |  |
| PsycINFO | Concept 1: Anxiety OR Stress | |  |
|  | #1 | exp Anxiety/ | 85,145 |
|  | #2 | (anxiet* or nervous* or anxious* or "social anxiety" or Angst or Hypervigilance or "anticipatory anxiety").ab,ti. | 289,546 |
|  | #3 | exp stress/ | 133,708 |
|  | #4 | ("psychological stress*" or "acute stress*" or "chronic stress*" or "mental stress*" or "stress*" or "life stress*" or "psychologic stress*").ab,ti. | 289,725 |
|  | #5 | #1 OR #2 OR #3 OR #4 | 560,300 |
|  | Concept 3: Healthcare students | |  |
|  | #6 | exp Nursing Students/ OR exp Dental Students/ | 6,487 |
|  | #7 | ("Health occupations student" or "Healthcare student" or "Allied health student" or "Nursing student" or "Physiotherapy student" or "Occupational therapy student" or "Dentistry student" or "Veterinary science student" or "Pharmacy student" or "Psychology student" or "Public health student" or "Social work student" or "Speech therapy student" or "Radiology student" or "Dietician student" or "Podiatry student").ab,ti. | 1,255 |
|  | #8 | #6 OR #7 | 7,252 |
|  | Merging Concepts: (Anxiety OR Stress) AND Healthcare Students | |  |
|  | #9 | #5 AND #8 | 999 |
|  |  |  |  |
| ProQuest Social Science Database | Concept 1: Anxiety OR Stress | |  |
|  | #1 | MAINSUBJECT.EXACT("Anxieties") | 15 |
|  | #2 | title(Nervous* OR Anxious* OR Anxiet* OR “Social Anxiety” OR Angst OR Hypervigilance OR “Anticipatory anxiety”) OR abstract(Nervous* OR Anxious* OR Anxiet* OR “Social Anxiety” OR Angst OR Hypervigilance OR “Anticipatory anxiety”) | 31,255 |
|  | #3 | MAINSUBJECT.EXACT("Stress") OR MAINSUBJECT.EXACT("Occupational stress") | 14.655 |
|  | #4 | title(Stress* OR “Psychologic Stress*” OR “Psychological Stress*” OR “Life Stress*” OR “Mental Stress*” OR “Acute Stress*” OR “Chronic Stress*”) OR abstract(Stress* OR “Psychologic Stress*” OR “Psychological Stress*” OR “Life Stress*” OR “Mental Stress*” OR “Acute Stress*” OR “Chronic Stress*”) | 42,059 |
|  | #5 | #1 OR #2 OR #3 OR #4 | 71,084 |
|  | Concept 3: Healthcare students | |  |
|  | #6 | MAINSUBJECT.EXACT("Nursing schools") OR MAINSUBJECT.EXACT("Midwifery education") OR MAINSUBJECT.EXACT("Nursing education") OR MAINSUBJECT.EXACT("Dental schools") | 495 |
|  | #7 | title(“Health occupations student” OR “Healthcare student” OR “Allied health student” OR “Nursing student” OR “Physiotherapy student” OR “Occupational therapy student” OR “Dentistry student” OR “Veterinary science student” OR “Pharmacy student” OR “Psychology student” OR “Public health student” OR “Social work student” OR “Speech therapy student” OR “Radiology student” OR “Dietician student” OR “Podiatry student”) OR abstract(“Health occupations student” OR “Healthcare student” OR “Allied health student” OR “Nursing student” OR “Physiotherapy student” OR “Occupational therapy student” OR “Dentistry student” OR “Veterinary science student” OR “Pharmacy student” OR “Psychology student” OR “Public health student” OR “Social work student” OR “Speech therapy student” OR “Radiology student” OR “Dietician student” OR “Podiatry student”) | 158 |
|  | #8 | #6 OR #7 | 642 |
|  | Merging Concepts: (Anxiety OR Stress) AND Healthcare Students | |  |
|  | #9 | #5 AND #8 | 40 |
|  |  |  |  |
| Scopus | Concept 1: Anxiety OR Stress | |  |
|  | #1 | TITLE-ABS-KEY ( anxiety  OR  nervousness  OR  anxiousness  OR  "Social Anxiety"  OR  "Performance anxiety"  OR  "Test anxiety"  OR  angst  OR  hypervigilance  OR  "Anticipatory anxiety"  OR  "Dental anxiety") | 491,653 |
|  | #2 | TITLE-ABS-KEY ( "Psychological Stress"  OR  "acute stress"  OR  "chronic stress"  OR  "mental stress"  OR  "physically induced stress"  OR  "surgical stress"  OR  "Life Stresses"  OR  "Life Stress"  OR  "Life stressor"  OR  "Psychologic Stress") | 157,822 |
|  | #3 | #1 OR #2 | 618,891 |
|  | Concept 3: Healthcare students | |  |
|  | #4 | TITLE-ABS-KEY ( "health occupations student"  OR  "healthcare student"  OR  "allied health student"  OR  "nursing student"  OR  "physiotherapy student"  OR  "occupational therapy student"  OR  "dentistry student"  OR  "veterinary science student"  OR  "pharmacy student"  OR  "psychology student"  OR  "public health student"  OR  "social work student"  OR  "speech therapy student"  OR  "radiology student"  OR  "dietician student"  OR  "podiatry student" | 55,016 |
|  | Merging Concepts: (Anxiety OR Stress) AND Healthcare Students | |  |
|  | #5 | #3 AND #4 | 2,862 |
|  |  |  |  |
| Web of Science | Concept 1: Anxiety OR Stress | |  |
|  | #1 | **TS=(Nervous* OR Anxious* OR Anxiet* OR “Social Anxiety” OR Angst OR Hypervigilance OR “Anticipatory anxiety”)** | 814,289 |
|  | #2 | **TS=(Stress* OR “Psychologic Stress*” OR “Psychological Stress*” OR “Life Stress*” OR “Mental Stress*” OR “Acute Stress*” OR “Chronic Stress*”)** | 2,459,916 |
|  | #3 | #1 OR #2 | 3,144,794 |
|  | Concept 3: Healthcare students | |  |
|  | #4 | **TS=(“Health occupations student” OR “Healthcare student” OR “Allied health student” OR “Nursing student” OR “Physiotherapy student” OR “Occupational therapy student” OR “Dentistry student” OR “Veterinary science student” OR “Pharmacy student” OR “Psychology student” OR “Public health student” OR “Social work student” OR “Speech therapy student” OR “Radiology student” OR “Dietician student” OR “Podiatry student”)** | 4,062 |
|  | Merging Concepts: (Anxiety OR Stress) AND Healthcare Students | |  |
|  | #5 | #3 AND #4 | 490 |
|  |  | |  |
| ProQuest Disseratations & Theses | Concept 1: Anxiety OR Stress | |  |
|  | #1 | title(Nervous* OR Anxious* OR Anxiet* OR “Social Anxiety” OR Angst OR Hypervigilance OR “Anticipatory anxiety”) OR abstract(Nervous* OR Anxious* OR Anxiet* OR “Social Anxiety” OR Angst OR Hypervigilance OR “Anticipatory anxiety”) | 84,739 |
|  | #2 | title(Stress* OR “Psychologic Stress*” OR “Psychological Stress*” OR “Life Stress*” OR “Mental Stress*” OR “Acute Stress*” OR “Chronic Stress*”) OR abstract(Stress* OR “Psychologic Stress*” OR “Psychological Stress*” OR “Life Stress*” OR “Mental Stress*” OR “Acute Stress*” OR “Chronic Stress*”) | 220,721 |
|  | #3 | #1 OR #2 | 291,742 |
|  | Concept 3: Healthcare students | |  |
|  | #4 | title(“Health occupations student” OR “Healthcare student” OR “Allied health student” OR “Nursing student” OR “Physiotherapy student” OR “Occupational therapy student” OR “Dentistry student” OR “Veterinary science student” OR “Pharmacy student” OR “Psychology student” OR “Public health student” OR “Social work student” OR “Speech therapy student” OR “Radiology student” OR “Dietician student” OR “Podiatry student”) OR abstract(“Health occupations student” OR “Healthcare student” OR “Allied health student” OR “Nursing student” OR “Physiotherapy student” OR “Occupational therapy student” OR “Dentistry student” OR “Veterinary science student” OR “Pharmacy student” OR “Psychology student” OR “Public health student” OR “Social work student” OR “Speech therapy student” OR “Radiology student” OR “Dietician student” OR “Podiatry student”) | 1,127 |
|  | Merging Concepts: (Anxiety OR Stress) AND Healthcare Students | |  |
|  | #5 | #3 AND #4 | 186 |

**Table S4** List of excluded studies

|  | **Author (Year)** | **Name of article** | **Reason for exclusion** |
| --- | --- | --- | --- |
| 1 | Abdel-Khalek, et al. (2005) | Anxiety and death anxiety in Egyptian and Spanish nursing students | Prevalence unreported |
| 2 | Al-Dabal, et al. (2010) | A comparative study of perceived stress among female medical and non-medical university students in Dammam, Saudi Arabia | Incorrect population |
| 3 | Alamri, et al. (2020) | Association of Test Anxiety with Temporomandibular Disorder in Health Professions Students: A Cross-Sectional Study | Prevalence unreported |
| 4 | Almalik, et al. (2018) | Clinical Anxiety among Saudi Postgraduate Pediatric Dentistry Students in Jeddah City | Prevalence unreported |
| 5 | Alshammari. (2019) | Anxiety among male nursing students in a Saudi University | Prevalence unreported |
| 6 | Alsini, et al. (2021) | A national survey of self-prescription of beta-blockers and their relation to undiscovered anxiety among medical and pharmacological students in Saudi Arabia | Prevalence unreported |
| 7 | Alzayyat, et al. (2016) | Correlates of Stress and Coping among Jordanian Nursing Students during Clinical Practice in Psychiatric/Mental Health Course | Prevalence unreported |
| 8 | Aradilla-Herrero, et al. (2006) | Effects of an emotional education program on death anxiety in nursing students | Prevalence unreported |
| 9 | Atreya, et al. (2022) | Assessment of fear, anxiety, obsession and functional impairment of COVID-19 amongst health-care workers and trainees: A cross-sectional study in Nepal | Prevalence unreported |
| 10 | Augner. (2015) | Depressive symptoms and perceived chronic stress predict test anxiety in nursing students | Prevalence unreported |
| 11 | Awadh, et l. (2013) | A comparison study of perceived stress and quality of life among master of pharmacy and non-pharmacy master's students | Prevalence unreported |
| 12 | Babaahmadi, et al. (2021) | Comparison Between Peer Learning and Conventional Methods in Biostatistics Course Among Postgraduate Nursing Students’ Final Score, Statistics and Test Anxiety: A Quasi-experimental Study with a Control Group | Prevalence unreported |
| 13 | Baksi, et al. (2017) | Effectiveness of the Preparatory Clinical Education on Nursing Students Anxiety: A Randomized Controlled Trial | Prevalence unreported |
| 14 | Bartlett, et al. (2016) | Comparison of mental health characteristics and stress between baccalaureate nursing students and non-nursing students | Prevalence unreported |
| 15 | Bati, et al. (2013) | Anxiety of first cadaver demonstration in medical, dentistry and pharmacy faculty students | Prevalence unreported |
| 16 | Beddoe, et al. (2004) | Does mindfulness decrease stress and foster empathy among nursing students? | Prevalence unreported |
| 17 | Berdida, et al. (2022) | Academic stress, COVID-19 anxiety, and quality of life among nursing students: The mediating role of resilience | Prevalence unreported |
| 18 | Bogardus, et al. (2021) | Depression, Anxiety, and Stress in Doctor of Physical Therapy Students: Analysis of Incidence and Lived Experiences | Prevalence unreported |
| 19 | Carvalho, et al. (2004) | Nursing undergraduates' anxiety about the first surgical instrumentation | Non-English |
| 20 | Chandrasekaran, et al. (2014) | Dental Students' Perception and Anxiety Levels during their First Local Anesthetic Injection | Prevalence unreported |
| 21 | Coelho Brito, et al. (2019) | ANÁLISE DA OCORRÊNCIA DE ESTRESSE ENTRE ESTUDANTES DE ENFERMAGEM | Non-English |
| 22 | Costa, et al. (2014) | Common mental disorders and associated factors among final-year healthcare students | Prevalence unreported |
| 23 | Denton, et al. (1977) | Death experience and death anxiety among nurses and nursing students | Prevalence unreported |
| 24 | Drake, et al. (2017) | A decade of counseling services in one college of veterinary medicine: Veterinary medical students' psychological distress and help-seeking trends | Prevalence unreported |
| 25 | Farrington. (1997) | Clinical management. Strategies for reducing stress and burnout in nursing | Prevalence unreported |
| 26 | Feith, et al. (2012) | Comparative study of future family plans and perceived stress factors among Hungarian students in higher education | Incorrect population |
| 27 | Figen, et al. (2020) | Correlation between Educational Stress and Professional Self-Esteem of Nursing Students | Prevalence unreported |
| 28 | Gomathi. (2020) | Effectiveness of selected yoga practices on the level of stress among student nurses in selected nursing educational institution, Dehradun | Prevalence unreported |
| 29 | Gorter, et al. (2020) | [Study stress, burnout and physical complaints among dental students] | Non-English |
| 30 | Gupta, et al. (2022) | Prevalence of Anxiety and its Severity among Different Specialties of Health Care Professionals during COVID-19 Pandemic | Incorrect population |
| 31 | Gutierrez, et al. (2011) | Baccalaureate nursing students' stress levels in neonatal intensive care units | Prevalence unreported |
| 32 | Hanna, et al. (2018) | A questionnaire study to investigate stress among future pharmacists by gender and year group | Prevalence unreported |
| 33 | Haskins. (2009) | An exploration of satisfaction, psychological stress, and readiness for interprofessional learning in medical, nursing, allied health, and social work students in an interprofessional health care course | Prevalence unreported |
| 34 | He, et al. (2018) | Assessing stress, protective factors and psychological well-being among undergraduate nursing students | Prevalence unreported |
| 35 | Hee Kyoung, et al. (2014) | A Concept Mapping Study on Clinical Stress for Nursing Students during Clinical Practice | Non-English |
| 36 | Heise. (2022) | Relationships of race-related stress to burnout and compassion fatigue among nursing students | Prevalence unreported |
| 37 | Jagroop-Dearing, et al. (2022) | COVID-19 Lockdown in New Zealand: Perceived Stress and Wellbeing among International Health Students Who Were Essential Frontline Workers | Prevalence unreported |
| 38 | Johnsen. (2014) | Effect of Aromatherapy on Cognitive Test Anxiety Among Nursing Students | Prevalence unreported |
| 39 | Jongh, et al. (1995) | Dutch students' dental anxiety and occurrence of thoughts related to treatment | Prevalence unreported |
| 40 | Kanderis Lane, et al. (2021) | A 15-Minute Yoga Intervention to Reduce Entry-Level Dental Hygiene Student Stress | Prevalence unreported |
| 41 | Karaca, et al. (2019) | Effects of a stress management training program with mindfulness-based stress reduction | Prevalence unreported |
| 42 | Karaduman, et al. (2022) | Coronavirus and Health Anxiety of Nursing Students in Two Different Countries | Prevalence unreported |
| 43 | Kim. (2003) | Baccalaureate nursing students' experiences of anxiety producing situations in the clinical setting | Prevalence unreported |
| 44 | Kinyon. (2021) | A Stress Reduction Intervention for First Semester Nursing Students During COVID-19 | Prevalence unreported |
| 45 | Köktürk Dalcalı, et al. (2021) | Anxiety levels and sleep quality in nursing students during the COVID-19 pandemic | Prevalence unreported |
| 46 | Kupcewicz, et al. (2020) | Analysis of the relationship between stress intensity and coping strategy and the quality of life of nursing students in Poland, Spain and Slovakia | Prevalence unreported |
| 47 | Kupcewicz, et al. (2022) | Correlation between Positive Orientation and Control of Anger, Anxiety and Depression in Nursing Students in Poland, Spain and Slovakia during the COVID-19 Pandemic | Prevalence unreported |
| 48 | Labrague, et al. (2018) | A cross-country comparative study on stress and quality of life in nursing students | Prevalence unreported |
| 49 | Laurence, et al. (2009) | Depressive Symptoms, Stress, and Social Support Among Dental Students at a Historically Black College and University | Prevalence unreported |
| 50 | Leggett. (2011) | Effectiveness of a brief stress reduction intervention for nursing students in reducing physiological stress indicators and improving well-being and mental health | Prevalence unreported |
| 51 | Leung, et al. (2017) | Burnout, stress and satisfaction among Australian and New Zealand radiation oncology trainees | Incorrect population |
| 52 | Lo. (2002) | A longitudinal study of perceived level of stress, coping and self-esteem of undergraduate nursing students: An Australian case study | Prevalence unreported |
| 53 | Machado, et al. (2020) | Anxiety and Sleep Quality in Dental Students at a Private Brazilian University | Non-English |
| 54 | Mafla, et al. (2015) | Burnout prevalence and correlates amongst Colombian dental students: the STRESSCODE study | Prevalence unreported |
| 55 | Mahmoudi, et al. (2020) | Coping styles with stress in nursing and midwifery students | Prevalence unreported |
| 56 | Marthiensen, et al. (2019) | Effects of a Brief Mindfulness Intervention on After-Degree Nursing Student Stress | Prevalence unreported |
| 57 | Martins, et al. (2017) | SITUAÇÕES INDUTORAS DE STRESS E BURNOUT EM ESTUDANTES DE ENFERMAGEM NOS ENSINOS CLÍNICOS | Non-English |
| 58 | Masha’al, et al. (2020) | Distance Learning-Related Stress Among Undergraduate Nursing Students During the COVID-19 Pandemic | Prevalence unreported |
| 59 | Matrínez-Arce, et al. (2021) | Anxiety levels in interprofessional teams during training with clinical simulation | Incorrect population |
| 60 | McCarthy, et al. (2018) | Coping with stressful events: A pre-post-test of a psycho-educational intervention for undergraduate nursing and midwifery students | Prevalence unreported |
| 61 | Mejía-Barrientos, et al. (2012) | Academic stress as a risk factor for dental caries | Prevalence unreported |
| 62 | Mir, et al. (2014) | Dentistry students ageing anxiety levels in northern Iran | Prevalence unreported |
| 63 | Moreira de Sousa, et al. (2018) | Anxiety, depression and academic performance: A study amongst Portuguese medical students versus non-medical students | Incorrect population |
| 64 | Moreno, et al. (2022) | Association between stress perceived and coping strategies among nursing students in times of COVID-19 | Prevalence unreported |
| 65 | Mussi, et al. (2019) | Comparison of stress in freshman and senior nursing students | Prevalence unreported |
| 66 | Nazir, et al. (2021) | A quantitative study of test anxiety and its influencing factors among medical and dental students | Prevalence unreported |
| 67 | Nguyen, et al. (2013) | Depression, anxiety, and suicidal ideation among Vietnamese secondary school students and proposed solutions: a cross-sectional study | Incorrect population |
| 68 | Oker, et al. (2021) | Comparison of the Relationship Between Death Anxiety and Depressive and Anxiety Symptoms Among Norwegian and Turkish Female Psychology Students | Prevalence unreported |
| 69 | Ollé, et al. (2016) | Anxiety in children submitted to dental appointment | Incorrect population |
| 70 | Onieva-Zafra, et al. (2020) | Anxiety, perceived stress and coping strategies in nursing students: a cross-sectional, correlational, descriptive study | Prevalence unreported |
| 71 | Özdelikara, et al. (2018) | Determination of health perception, health anxiety and effecting factors among nursing students | Prevalence unreported |
| 72 | Pavan, et al. (2021) | Covid-19 impact on the mental health of indian pharmacy students: An online survey | Prevalence unreported |
| 73 | Pereira, et al. (2014) | Assessment of stress in the inclusion of nursing students in hospital practice | Prevalence unreported |
| 74 | Pereira, et al. (2016) | Biofeedback and stress management in university first year nursing students | Prevalence unreported |
| 75 | Peretz, et al. (2000) | Dental anxiety among Israeli dental students: a 4-year longitudinal study | Prevalence unreported |
| 76 | Pfeifer, et al. (2022) | An investigation of stress and anxiety among health professions students in the early stages of the COVID-19 pandemic | Prevalence unreported |
| 77 | Phun, et al. (2010) | Alcohol consumption and stress in second year nursing students | Prevalence unreported |
| 78 | Pourafzal, et al. (2013) | Relationship between Perceived Stress with Resilience among Undergraduate Nursing Students | Prevalence unreported |
| 79 | Ramyarathidevi, et al. (2020) | Assessment of level of academic stress and stress coping style on impact of lockdown COVID 19 among nursing students at SRM College of Nursing, Chengalpattu (DT) | Prevalence unreported |
| 80 | Romo-Barrientos, et al. (2019) | Anatomical prosection practices in the Occupational Therapy degree. Student anxiety levels and academic effectiveness | Prevalence unreported |
| 81 | Romo-Barrientos, et al. (2020) | Anxiety levels among health sciences students during their first visit to the dissection room | Prevalence unreported |
| 82 | Rosburg. (1988) | A comparison of the anxiety levels of college students: nurses, police, and firefighters vs. general college students | Incorrect population |
| 83 | Sabourin, et al. (2019) | Assessment of mental health in doctor of pharmacy students | Prevalence unreported |
| 84 | Sanders, et al. (2002) | Effect of perceived stress on student performance in dental school | Prevalence unreported |
| 85 | Sandoval, et al. (2021) | Depression, Stress and Anxiety in Students of Human Medicine in Ayacucho (Peru) in the Context of the COVID-19 Pandemic | Prevalence unreported |
| 86 | Saul, et al. (2021) | Assessment of Stress and Quality of Life in Non-native and Native English-speaking Pharmacy Students | Prevalence unreported |
| 87 | Sedgeman. (2009) | Effects of a Mind-Consciousness-Thought (MCT) intervention on stress and well-being in freshman nursing students | Prevalence unreported |
| 88 | Seedhom, ete al. (2019) | Predictors of perceived stress among medical and nonmedical college students, Minia, Egypt | Incorrect population |
| 89 | Shahsavari, et al. (2017) | Effect of a clinical skills refresher course on the clinical performance, anxiety and self-efficacy of the final year undergraduate nursing students | Prevalence unreported |
| 90 | Siebern. (2009) | A study of the mediating effects of sleep on stress, health outcome and exam performance | Prevalence unreported |
| 91 | Simukonda, et al. (1989) | Anxiety in male nursing students at Kamuzu College of Nursing | Prevalence unreported |
| 92 | Stubin. (2020) | Clinical stress among undergraduate nursing students: Perceptions of clinical nursing faculty | Prevalence unreported |
| 93 | Tang, et al. (2021) | Associations between demographic characteristics, perceived threat, perceived stress, coping responses and adherence to COVID-19 prevention measures among Chinese healthcare students | Prevalence unreported |
| 94 | Theroux, et al. (2019) | A cross-sectional study of the association between anxiety and temporomandibular disorder in Australian chiropractic students | Prevalence unreported |
| 95 | Thomas, et al. (2016) | Dental Anxiety among Dental, Medical, and Nursing Students in India and Its Correlation Their Field of Study | Prevalence unreported |
| 96 | Turan, et al. (2021) | Analysis of anxiety levels and attitudes of nursing students toward the nursing profession during the COVID-19 pandemic | Prevalence unreported |
| 97 | Washington. (2009) | Effects of anxiety reducing interventions on performance anxiety in graduate nurses | Prevalence unreported |
| 98 | Watson, et al. (2009) | A longitudinal study of stress and psychological distress in nurses and nursing students | Prevalence unreported |
| 99 | Wilson. (1991) | Computer anxiety in nursing students | Prevalence unreported |
| 100 | Wu, et al. (2020) | Effects of anxiety on dental students' noncognitive performance in their first objective structured clinical examination | Prevalence unreported |
| 101 | Xiao, et al. (2022) | Anxiety, depression, and satisfaction with life among college students in China: Nine months after initiation of the outbreak of COVID-19 | Incorrect population |
| 102 | Yan, et al. (2014) | Attitudes Toward Death Anxiety and Dying Among Physiotherapy Students In Singapore | Prevalence unreported |
| 103 | Zarzecka, et al. (2021) | Dental environmental stress during the COVID-19 pandemic at the Jagiellonian University Medical College, Kraków, Poland | Prevalence unreported |

**
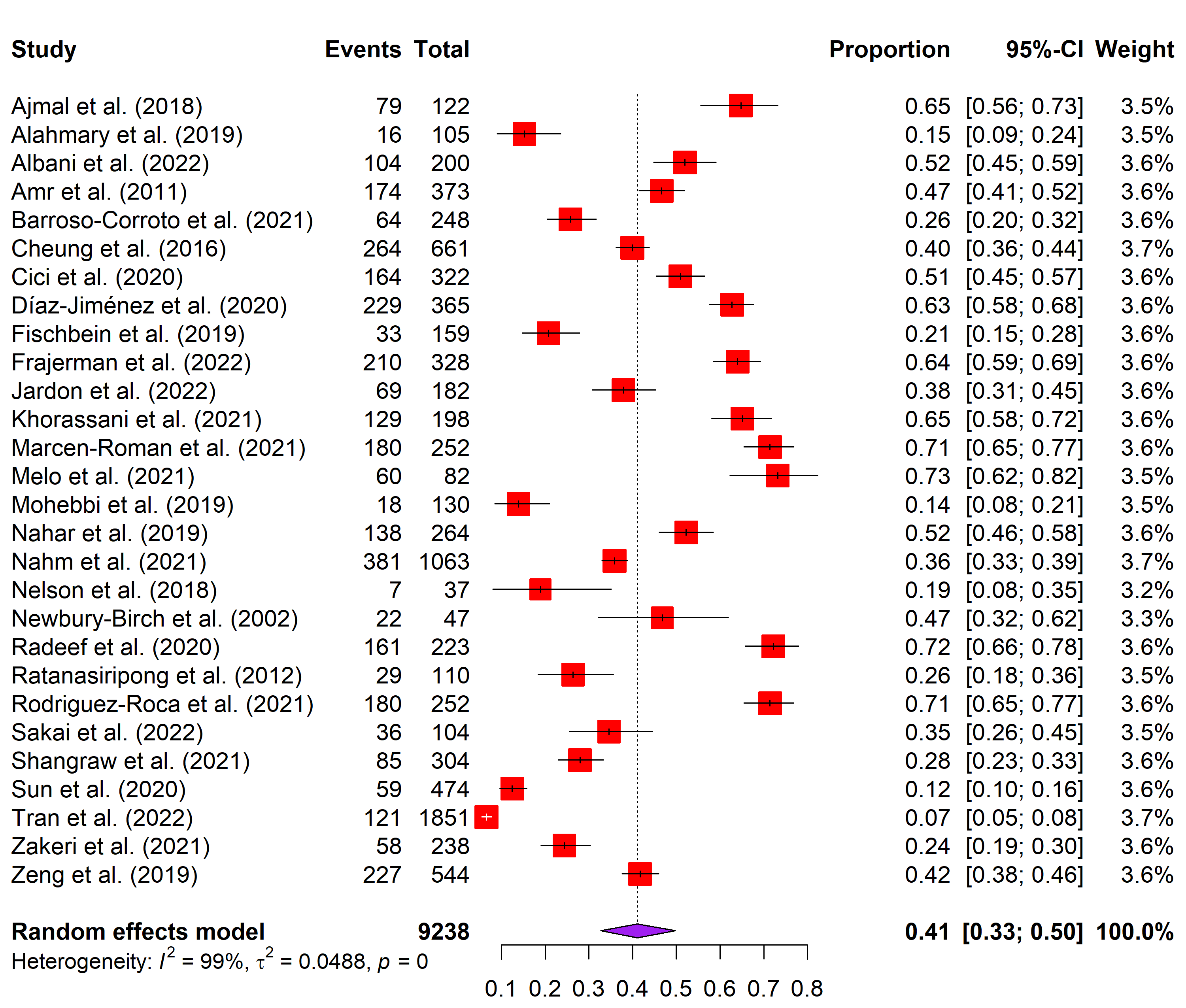
**

**Fig. S1** Forest plot of global prevalence (95% confidence interval) of unspecific anxiety symptoms among healthcare students

**
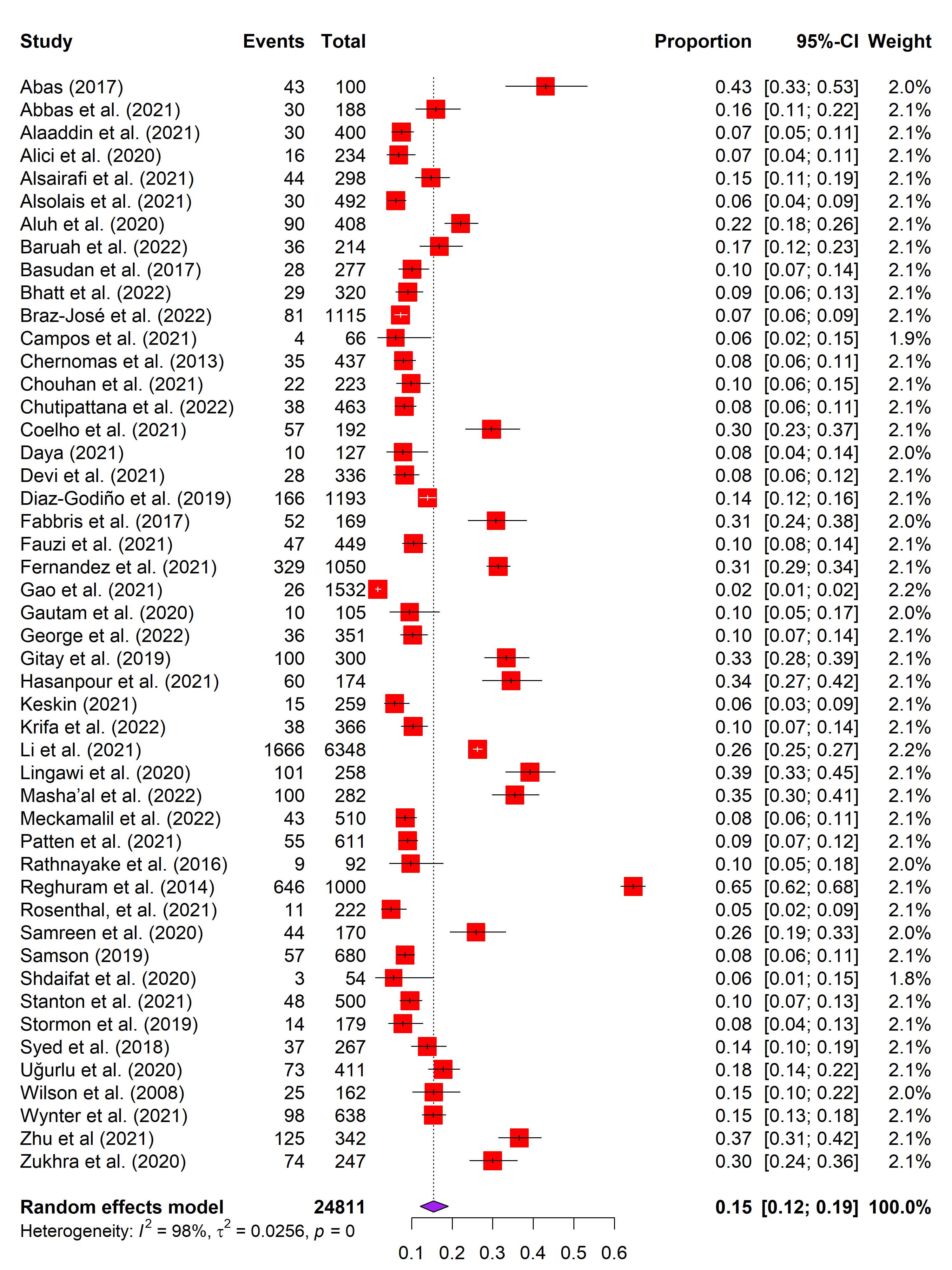
**

**Fig. S2** Forest plot of global prevalence (95% confidence interval) of mild anxiety symptoms among healthcare students

**
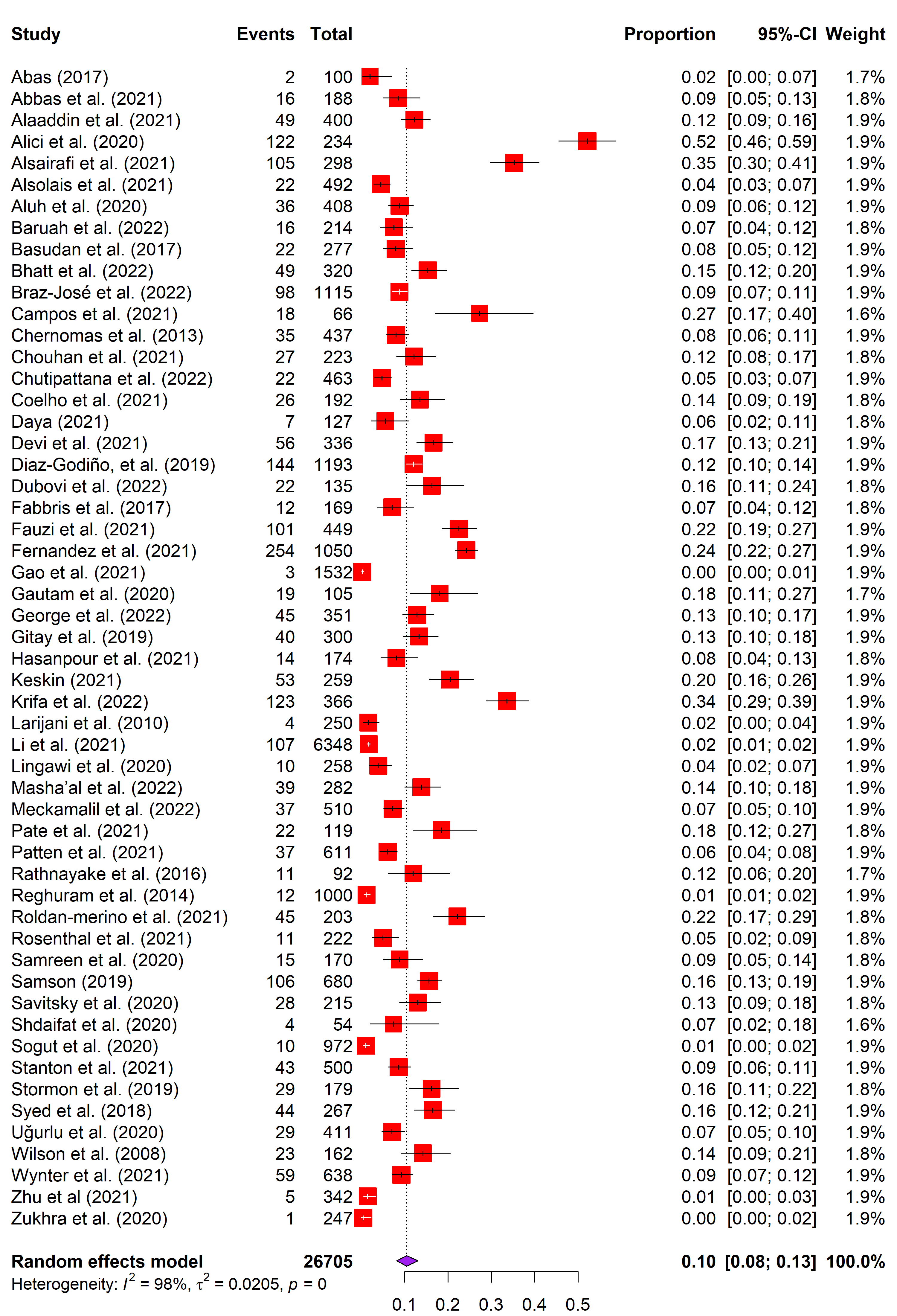
**

**Fig. S3** Forest plot of global prevalence (95% confidence interval) of severe anxiety symptoms among healthcare students

**
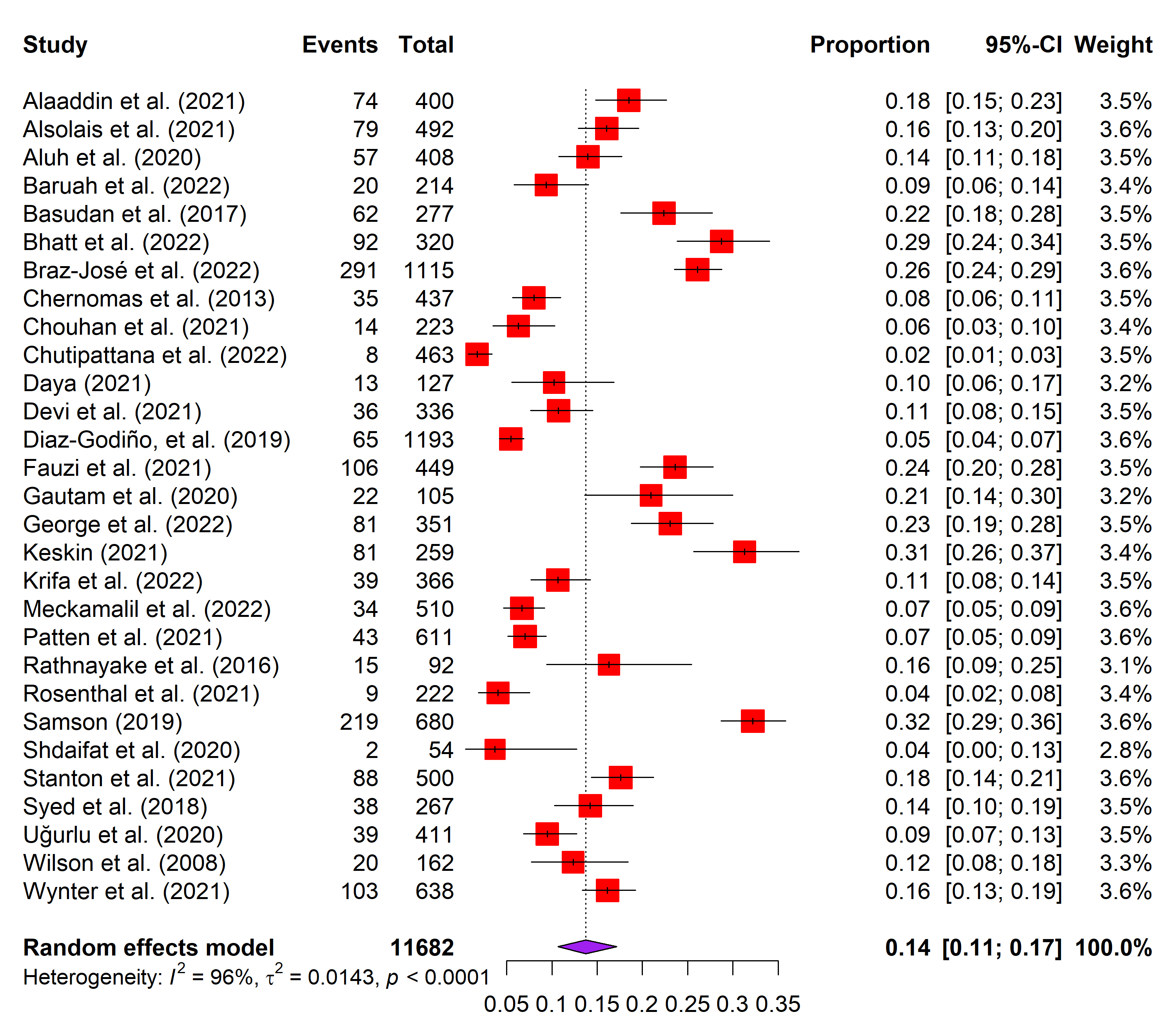
**

**Fig. S4** Forest plot of global prevalence (95% confidence interval) of extremely severe anxiety symptoms among healthcare students

**
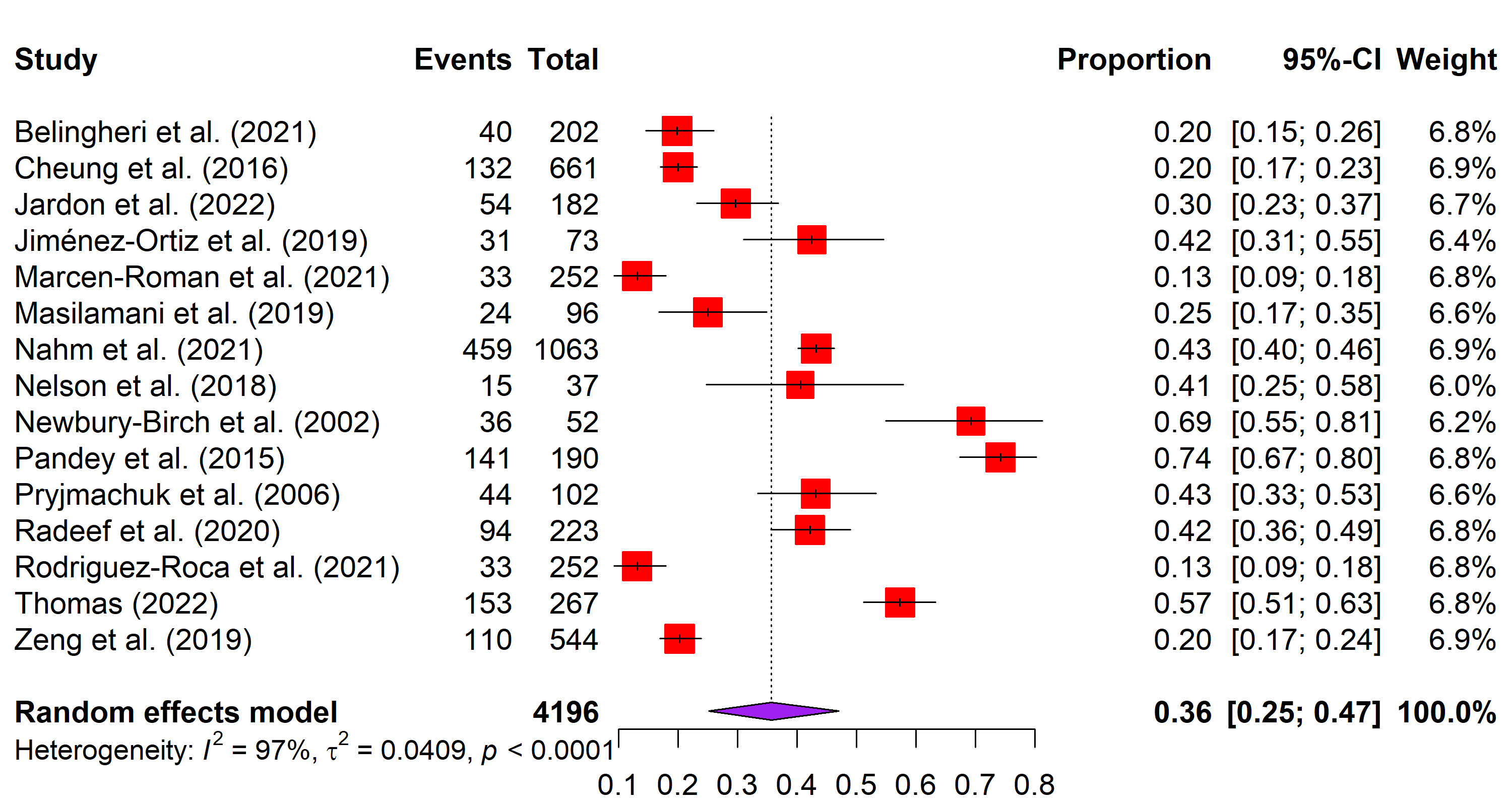
**

**Fig. S5** Forest plot of global prevalence (95% confidence interval) of unspecified stress symptoms among healthcare students

**
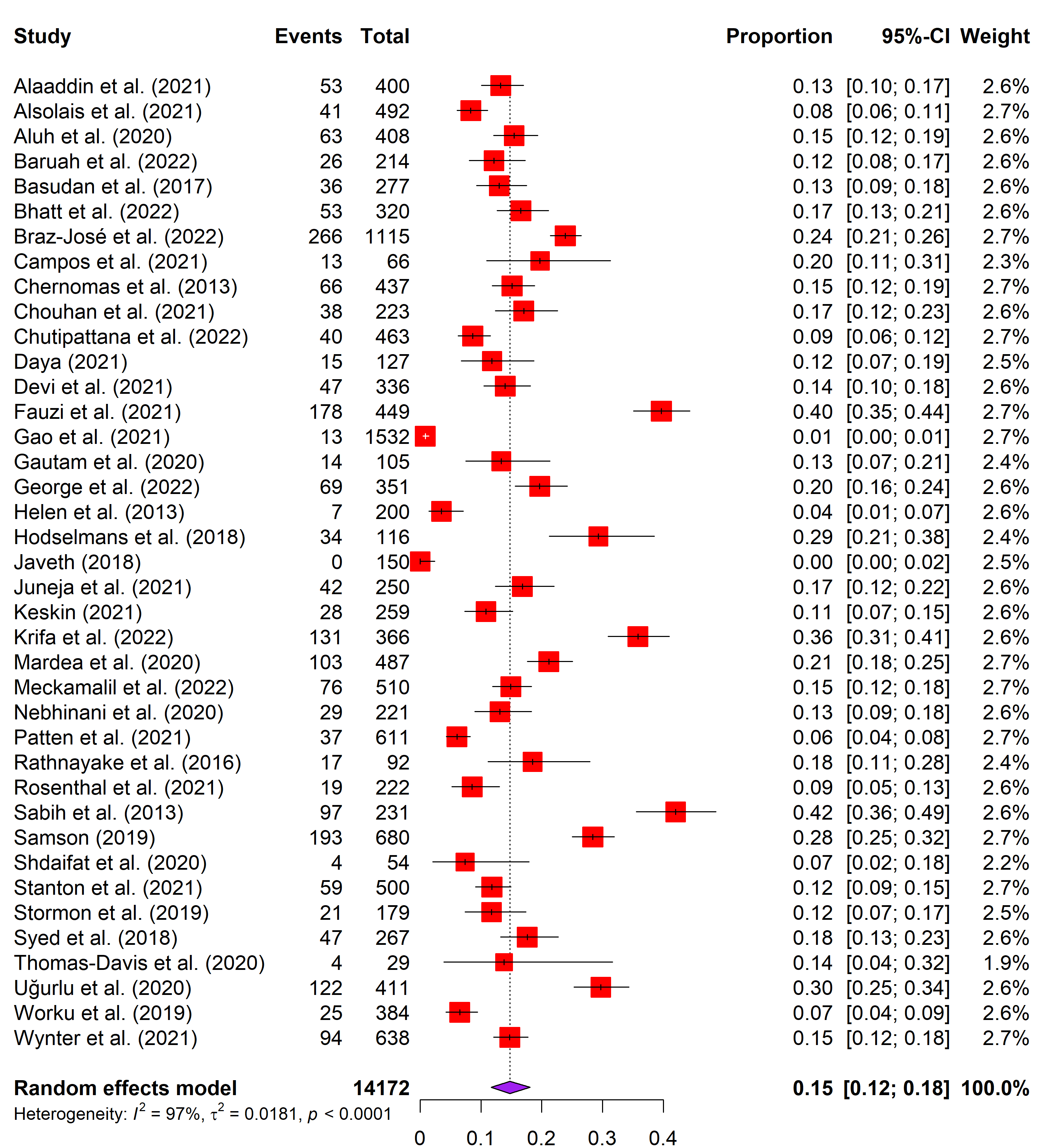
**

**Fig. S6** Forest plot of global prevalence (95% confidence interval) of mild stress symptoms among healthcare students

**
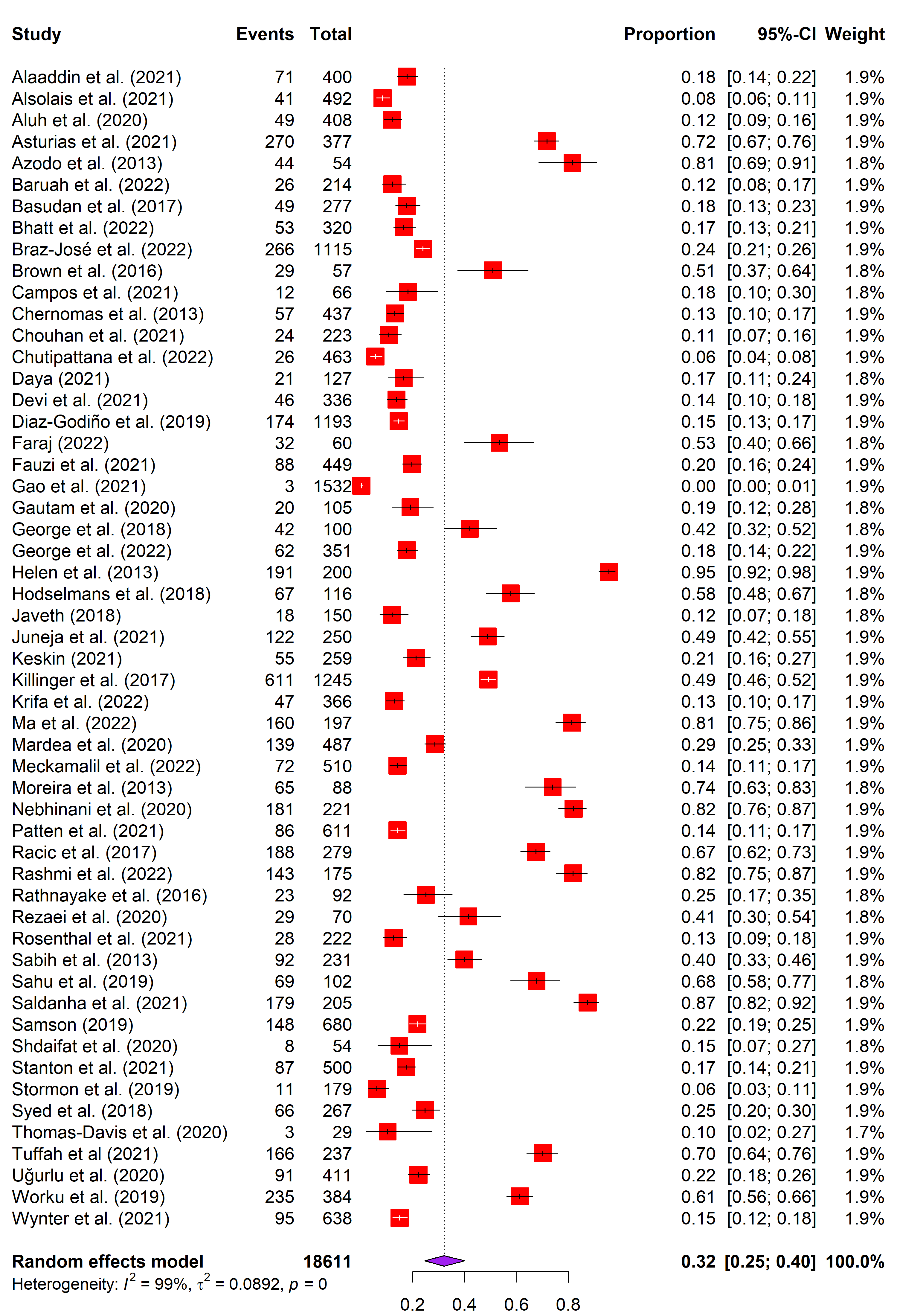
**

**Fig. S7** Forest plot of global prevalence (95% confidence interval) of moderate stress symptoms among healthcare students

**
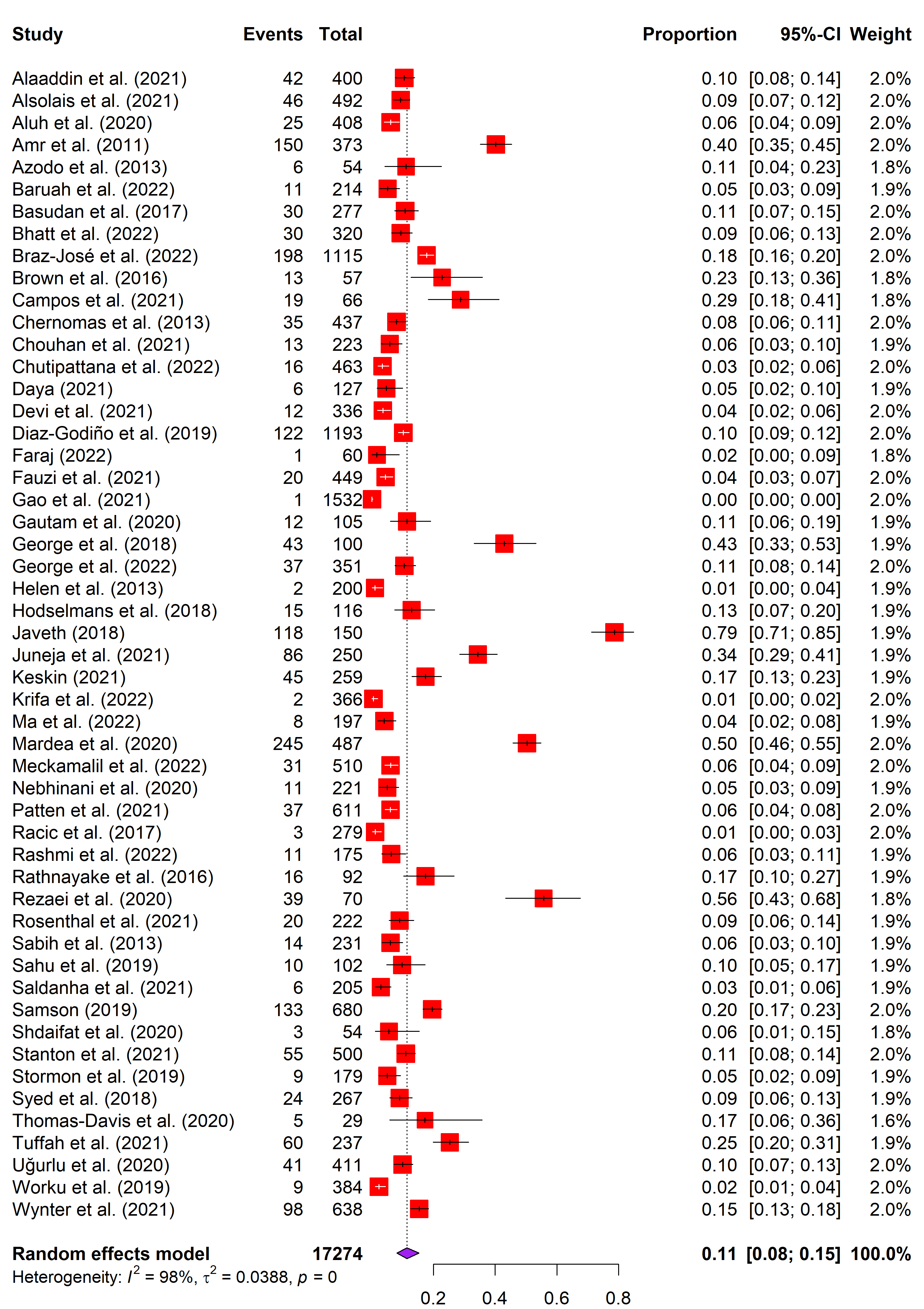
**

**Fig. S8** Forest plot of global prevalence (95% confidence interval) of severe stress symptoms among healthcare students.

**
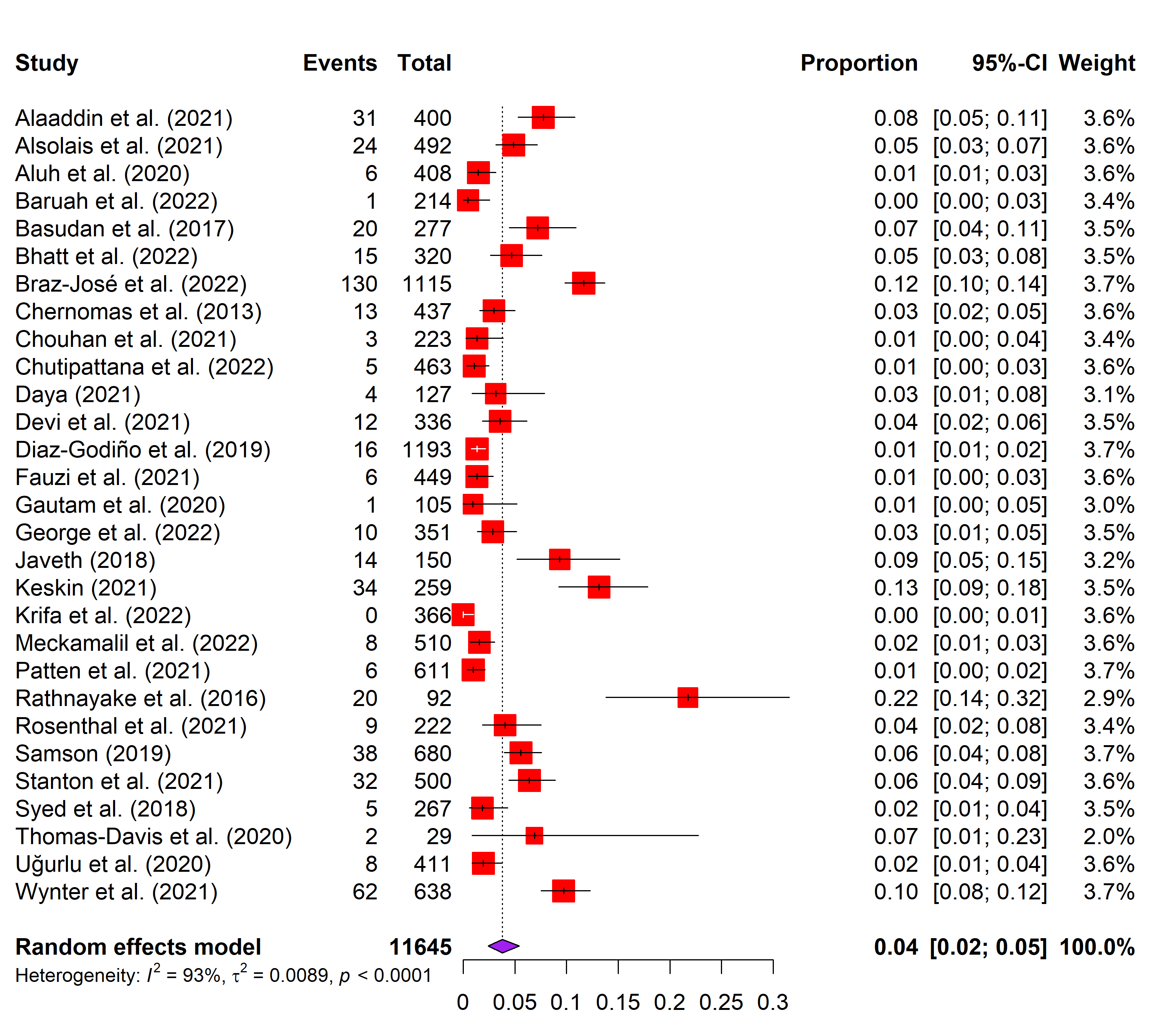
**

**Fig. S9** Forest plot of global prevalence (95% confidence interval) of extremely severe stress symptoms among healthcare students.


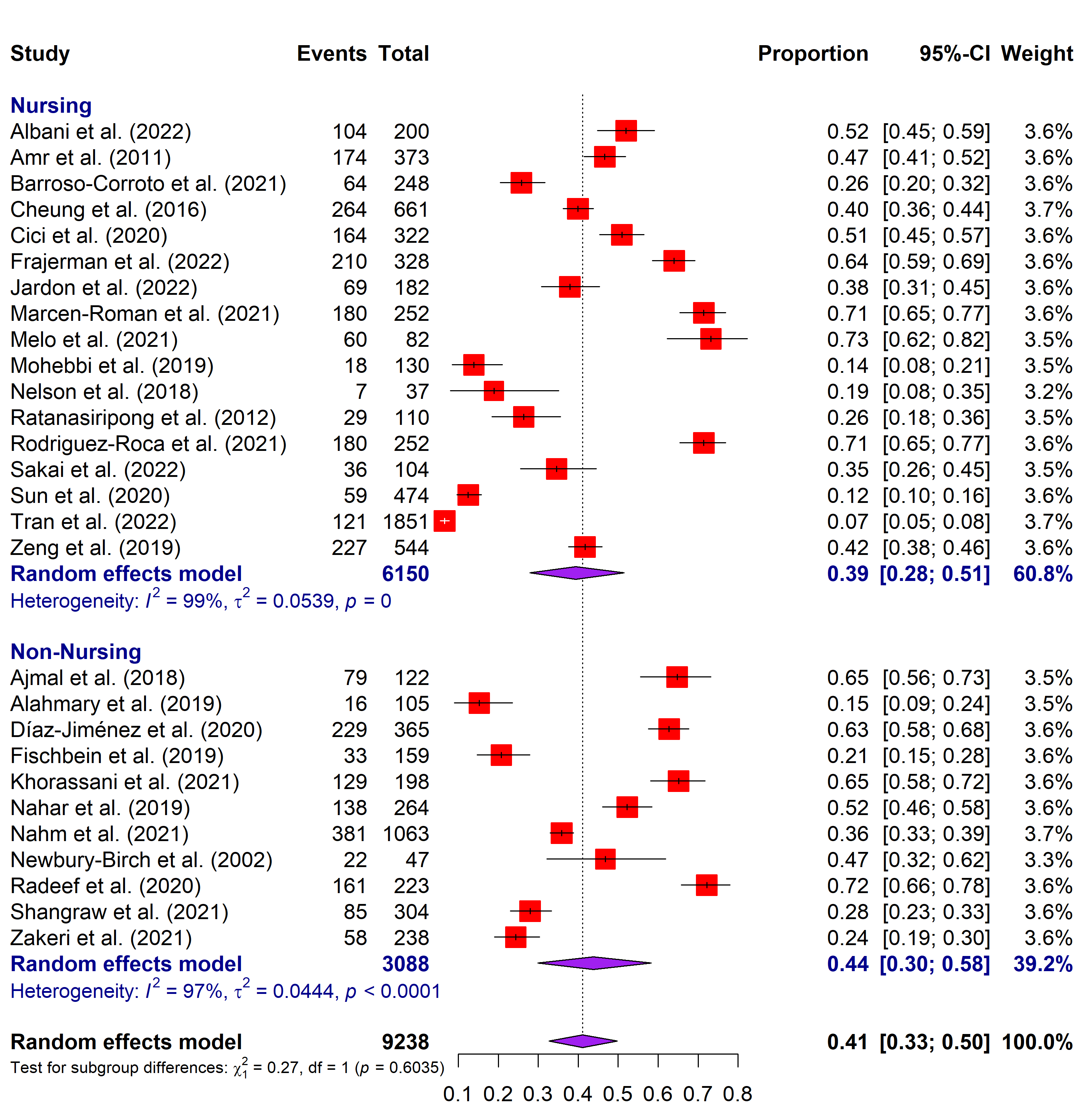


**Fig. S10** Subgroup analysis of global prevalence (95% confidence interval) of unspecific anxiety symptoms by types of students.

**
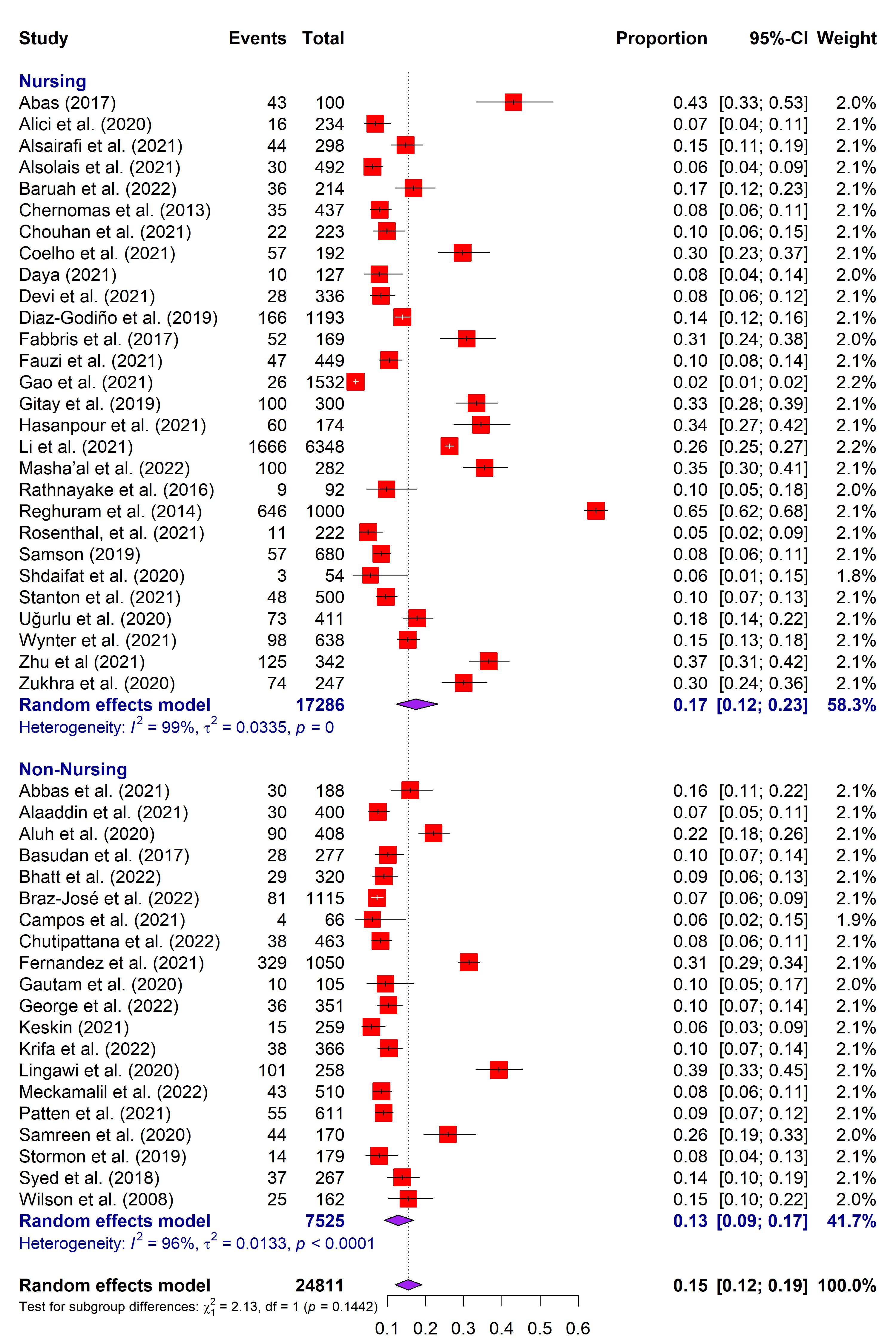
**

**Fig. S11** Subgroup analysis of global prevalence (95% confidence interval) of mild anxiety symptoms by types of students.

**
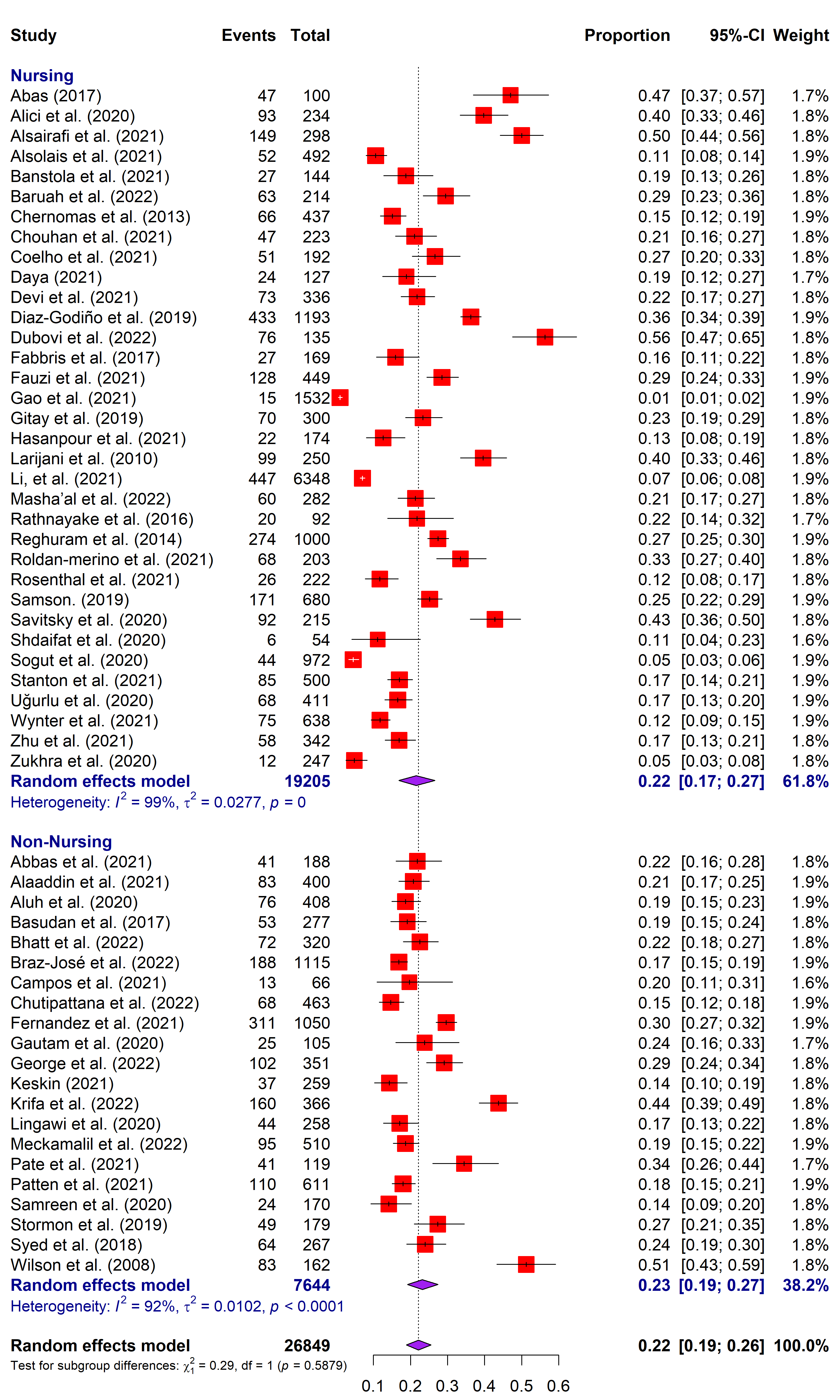
**

**Fig. S12** Subgroup analysis of global prevalence (95% confidence interval) of moderate anxiety symptoms by types of students.

**
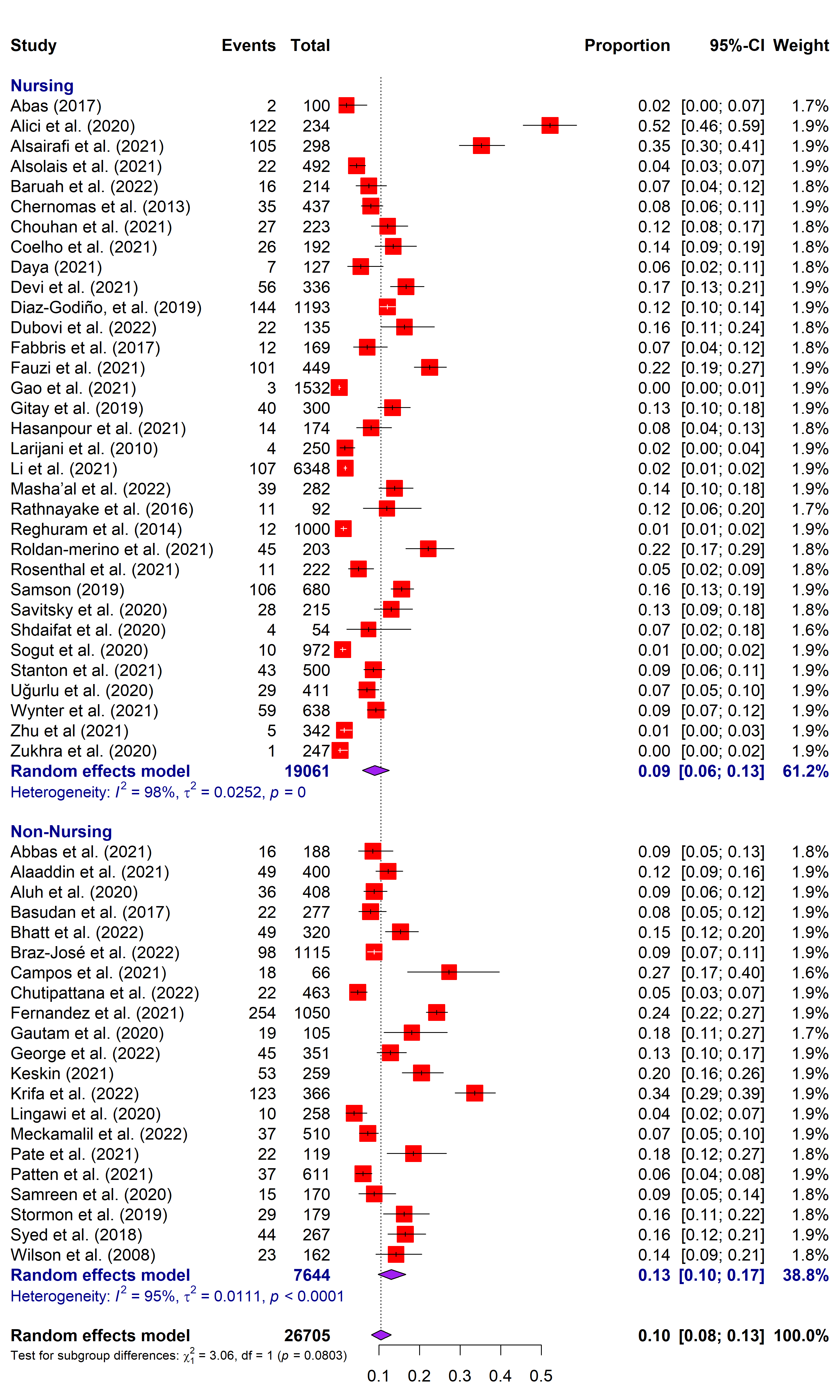
**

**Fig. S13** Subgroup analysis of global prevalence (95% confidence interval) of severe anxiety symptoms by types of students.

**
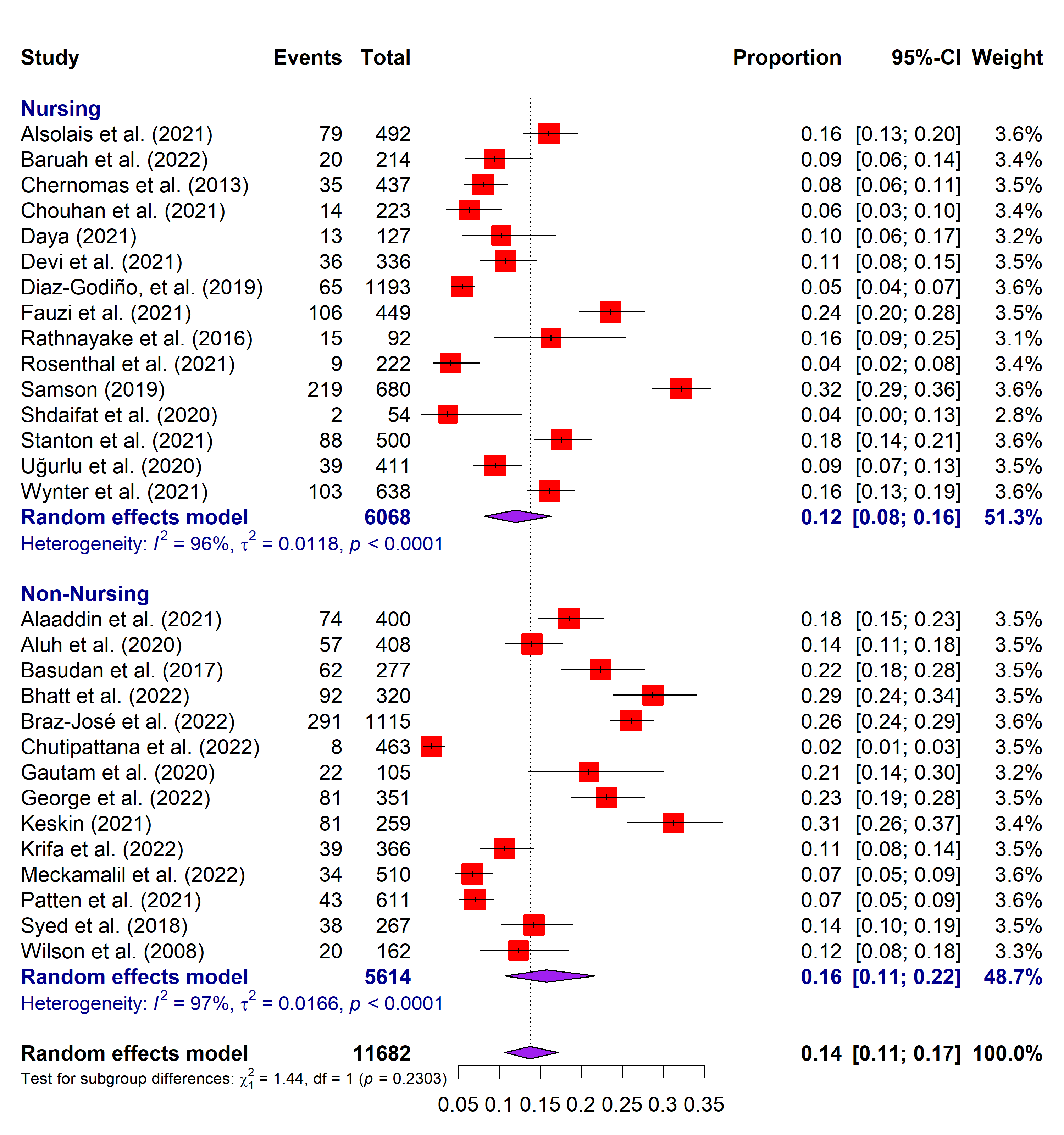
**

**Fig. S14** Subgroup analysis of global prevalence (95% confidence interval) of extremely severe anxiety symptoms by types of students.

**
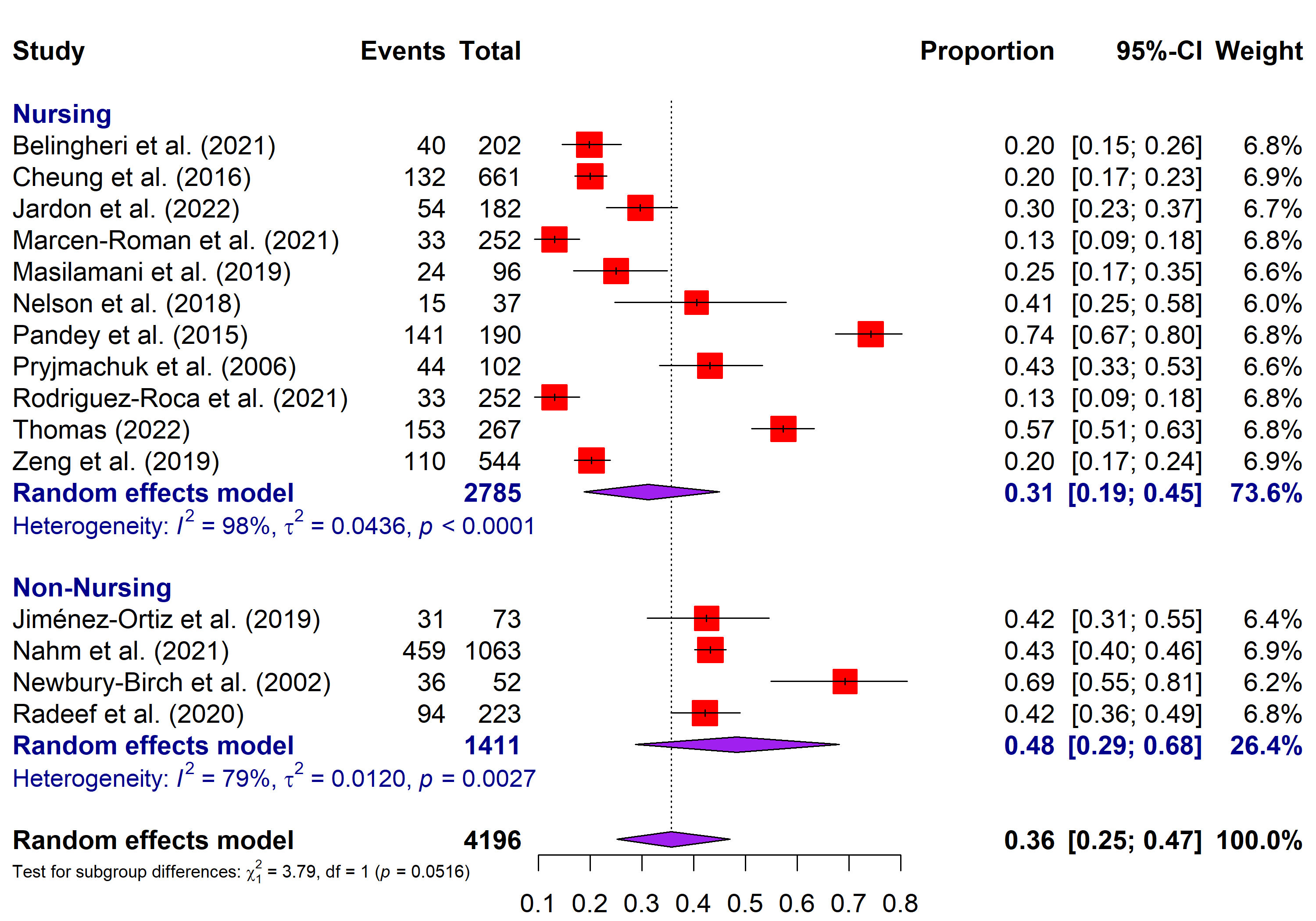
**

**Fig. S15** Subgroup analysis of global prevalence (95% confidence interval) of unspecific stress symptoms by types of students.

**
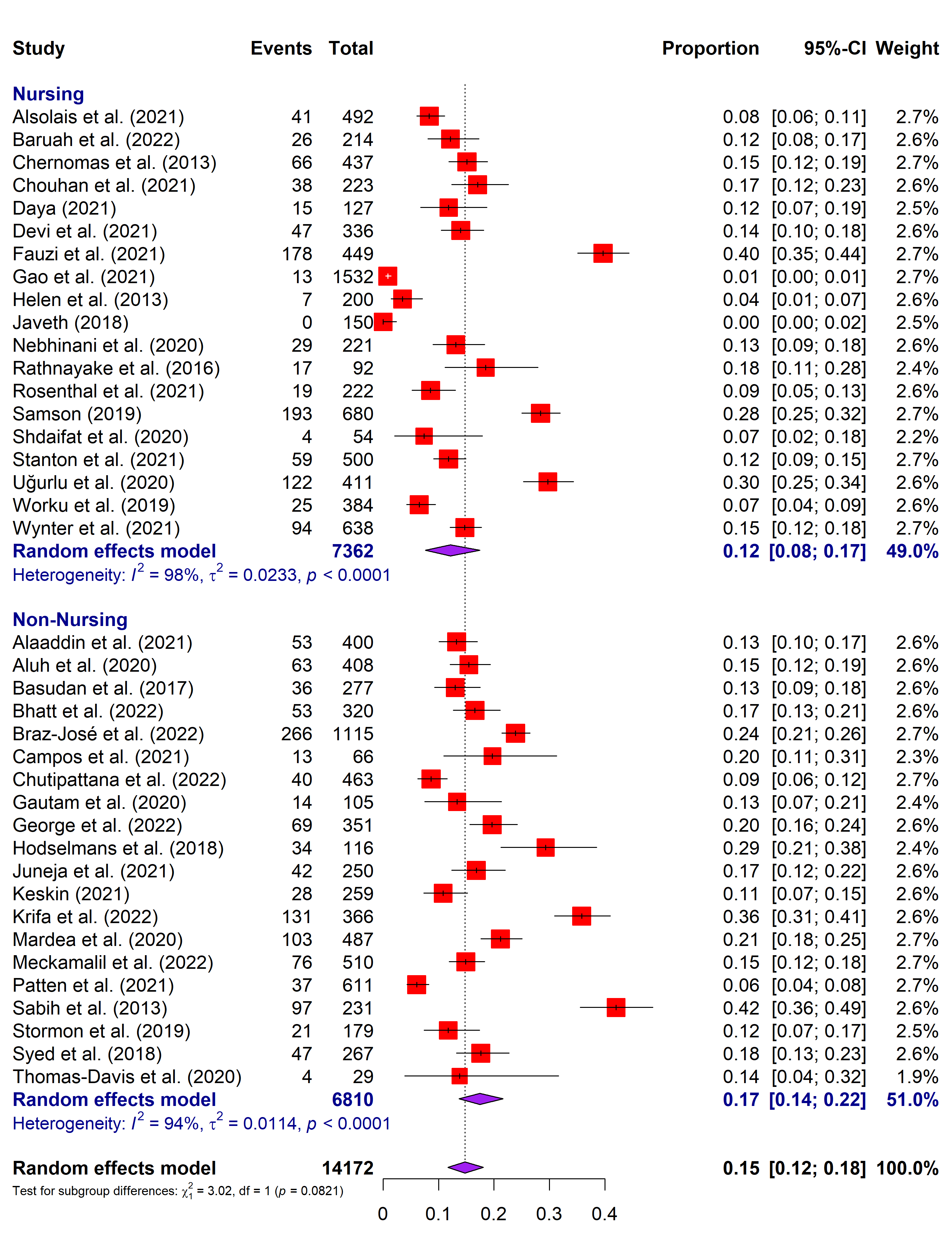
**

**Fig. S16** Subgroup analysis of global prevalence (95% confidence interval) of mild stress symptoms by types of students.

**
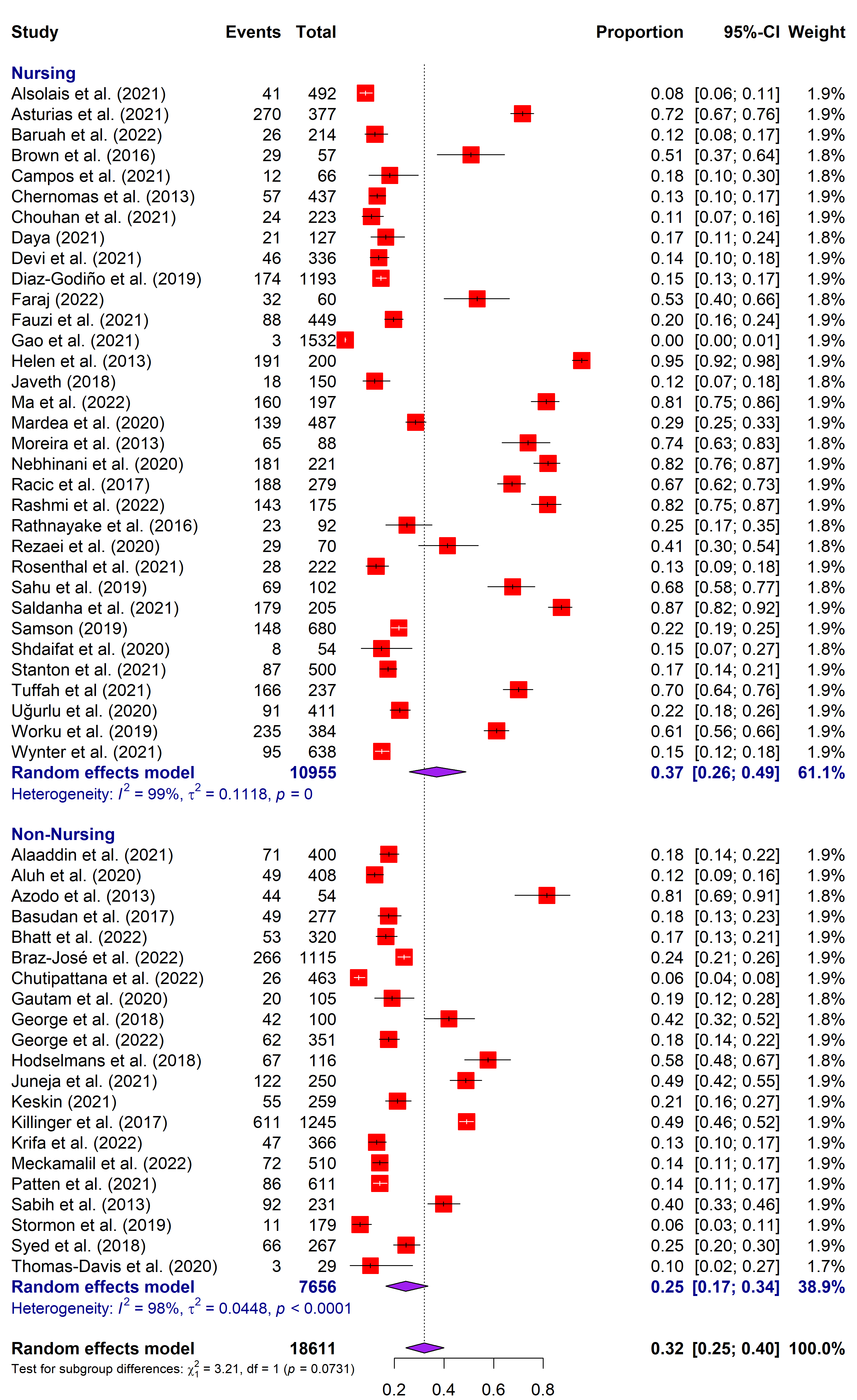
**

**Fig. S17** Subgroup analysis of global prevalence (95% confidence interval) of moderate stress symptoms by types of students.

**
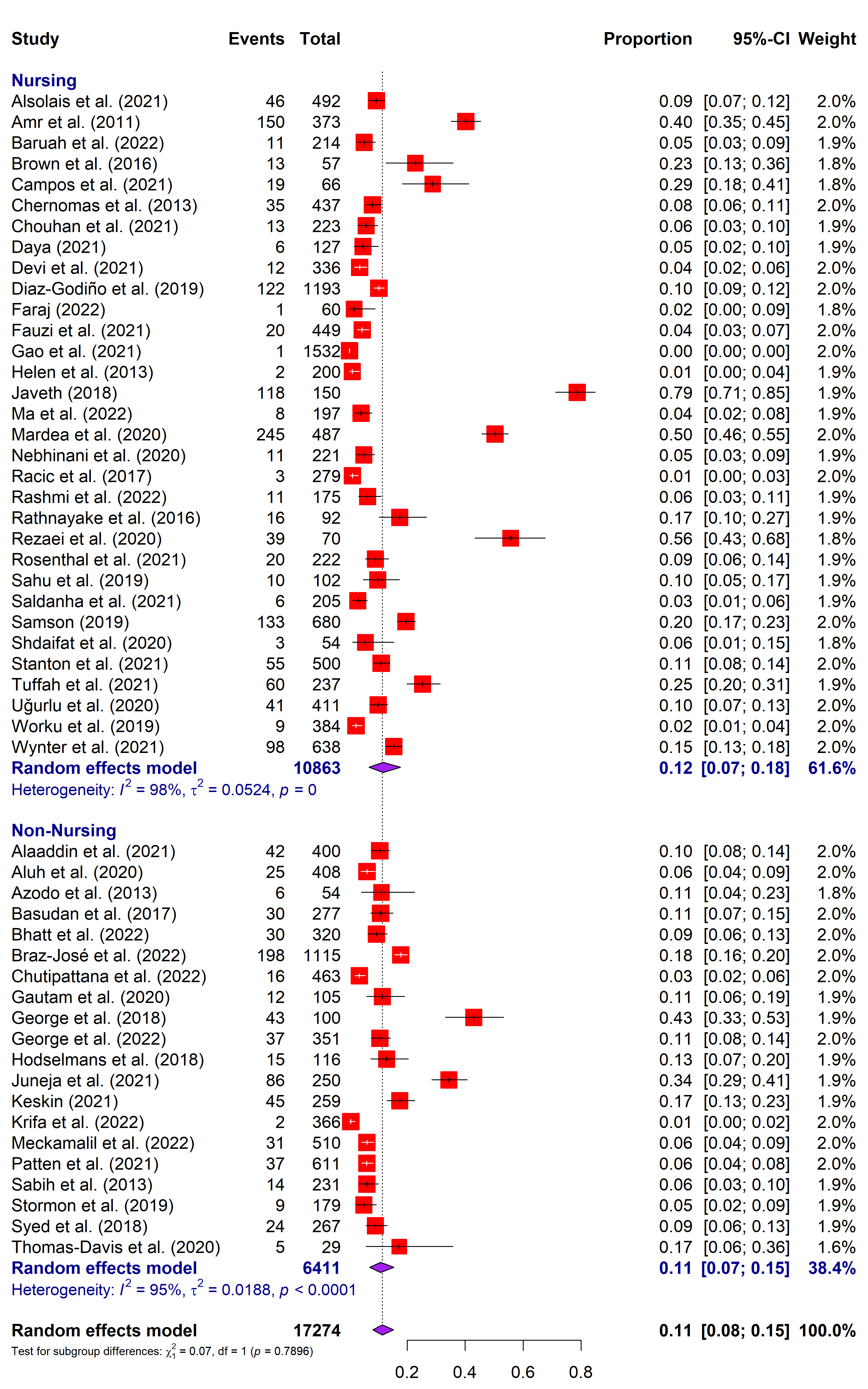
**

**Fig. S18** Subgroup analysis of global prevalence (95% confidence interval) of severe stress symptoms by types of students.

**
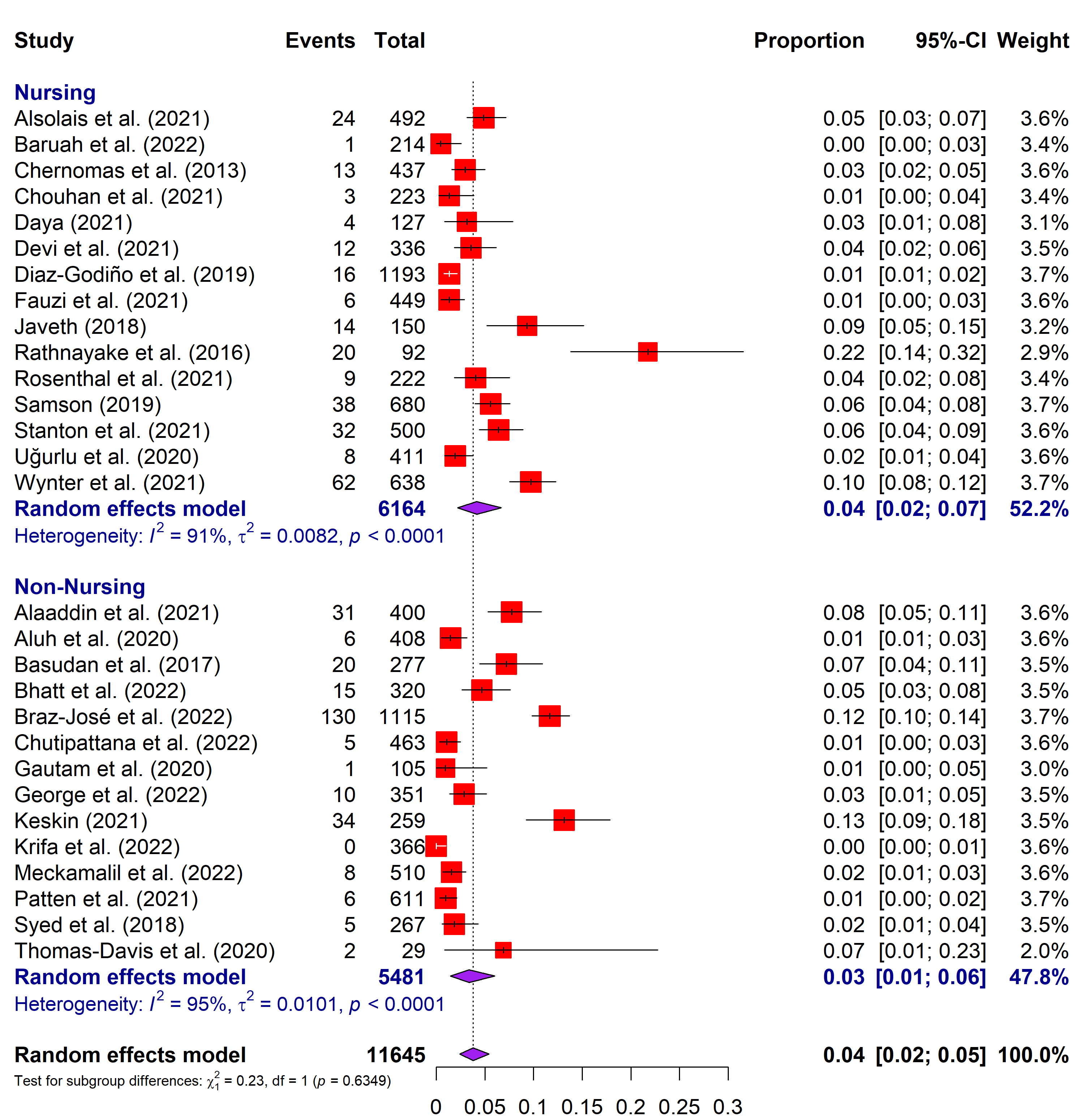
**

**Fig. S19** Subgroup analysis of global prevalence (95% confidence interval) of extremely stress symptoms by types of students.

**
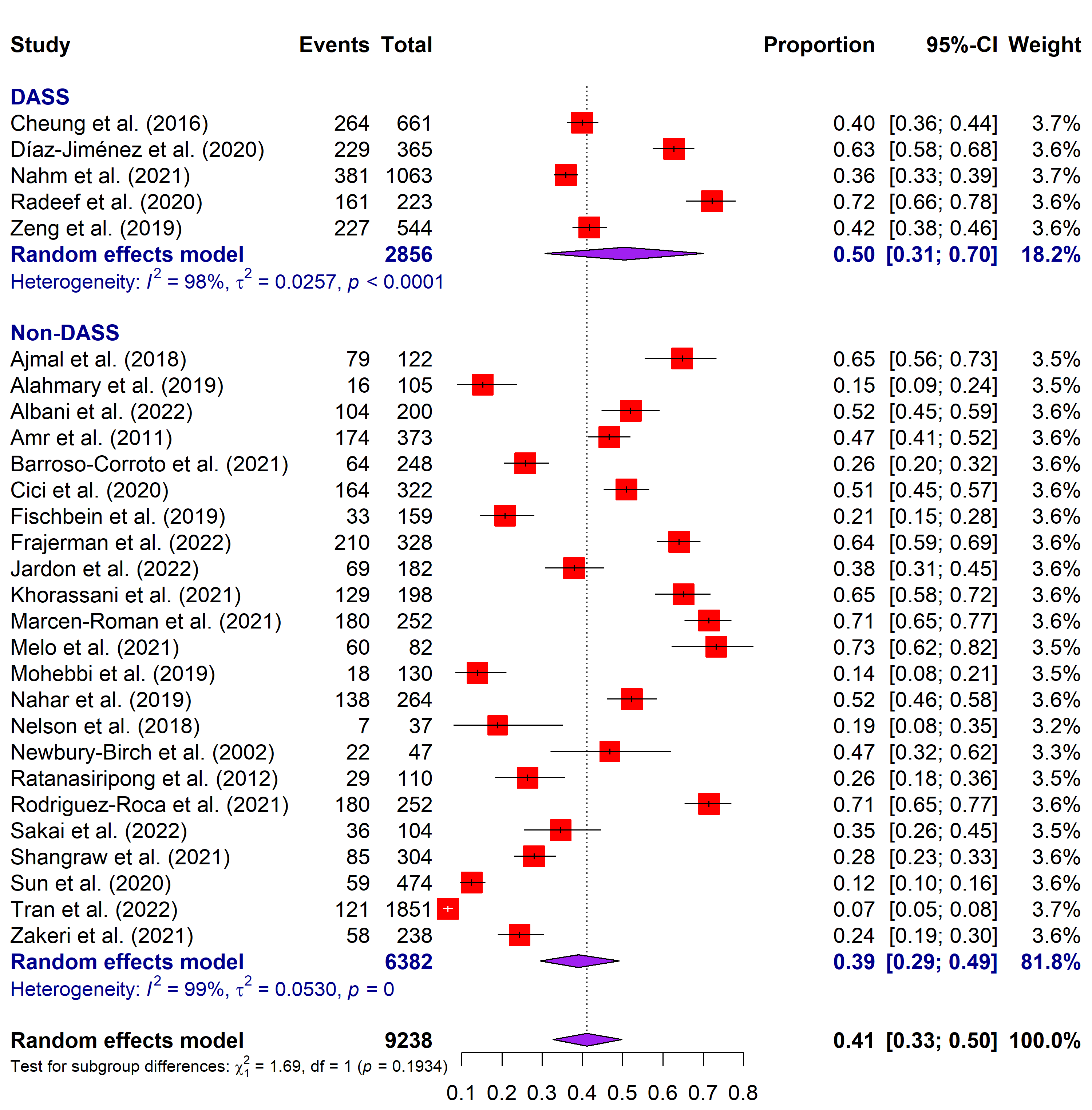
**

**Fig. S20** Subgroup analysis of global prevalence (95% confidence interval) of unspecific anxiety symptoms by types of measurement.

**
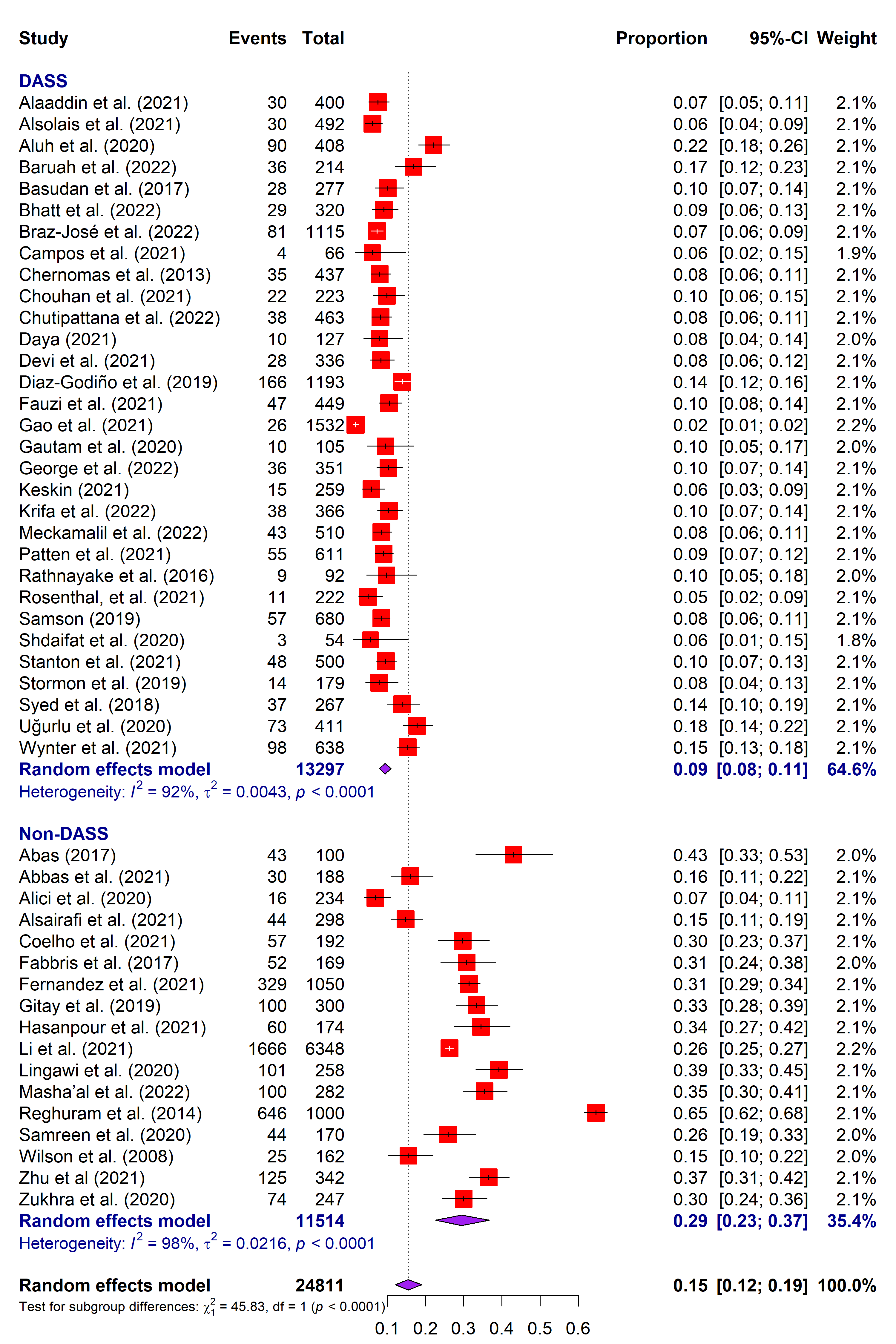
**

**Fig. S21** Subgroup analysis of global prevalence (95% confidence interval) of mild anxiety symptoms by types of measurement.

**
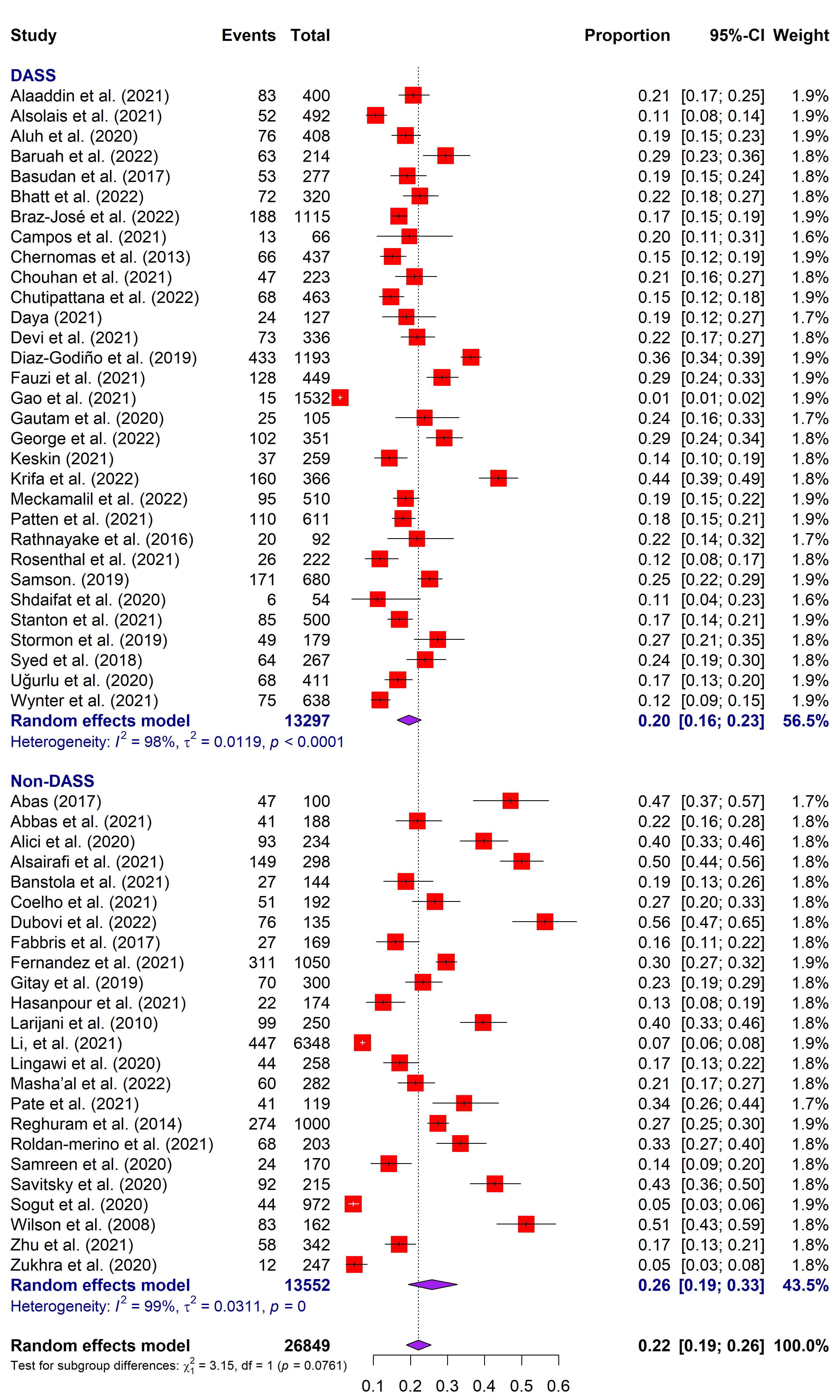
**

**Fig. S22** Subgroup analysis of global prevalence (95% confidence interval) of moderate anxiety symptoms by types of measurement.

**
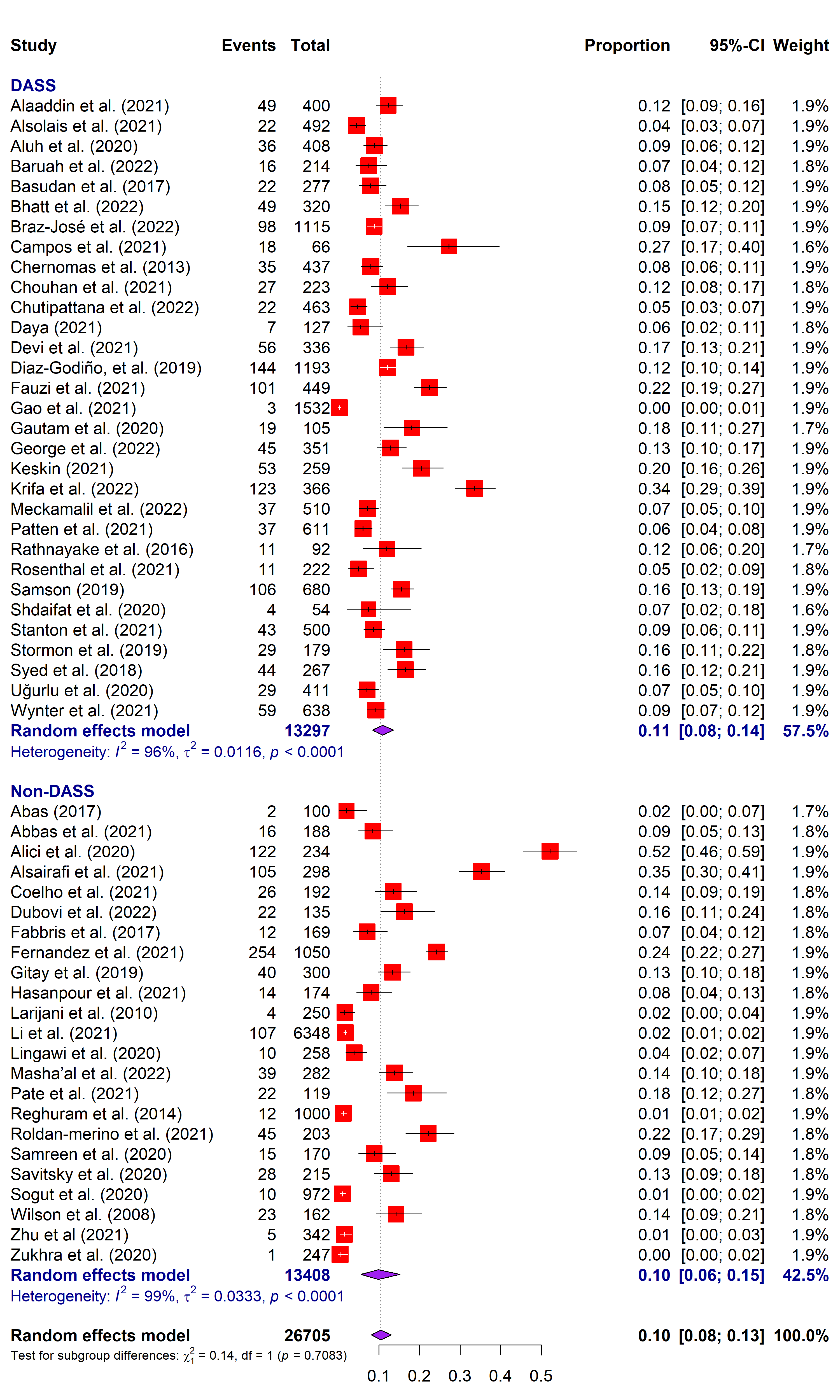
**

**Fig. S23** Subgroup analysis of global prevalence (95% confidence interval) of severe anxiety symptoms by types of measurement.

**
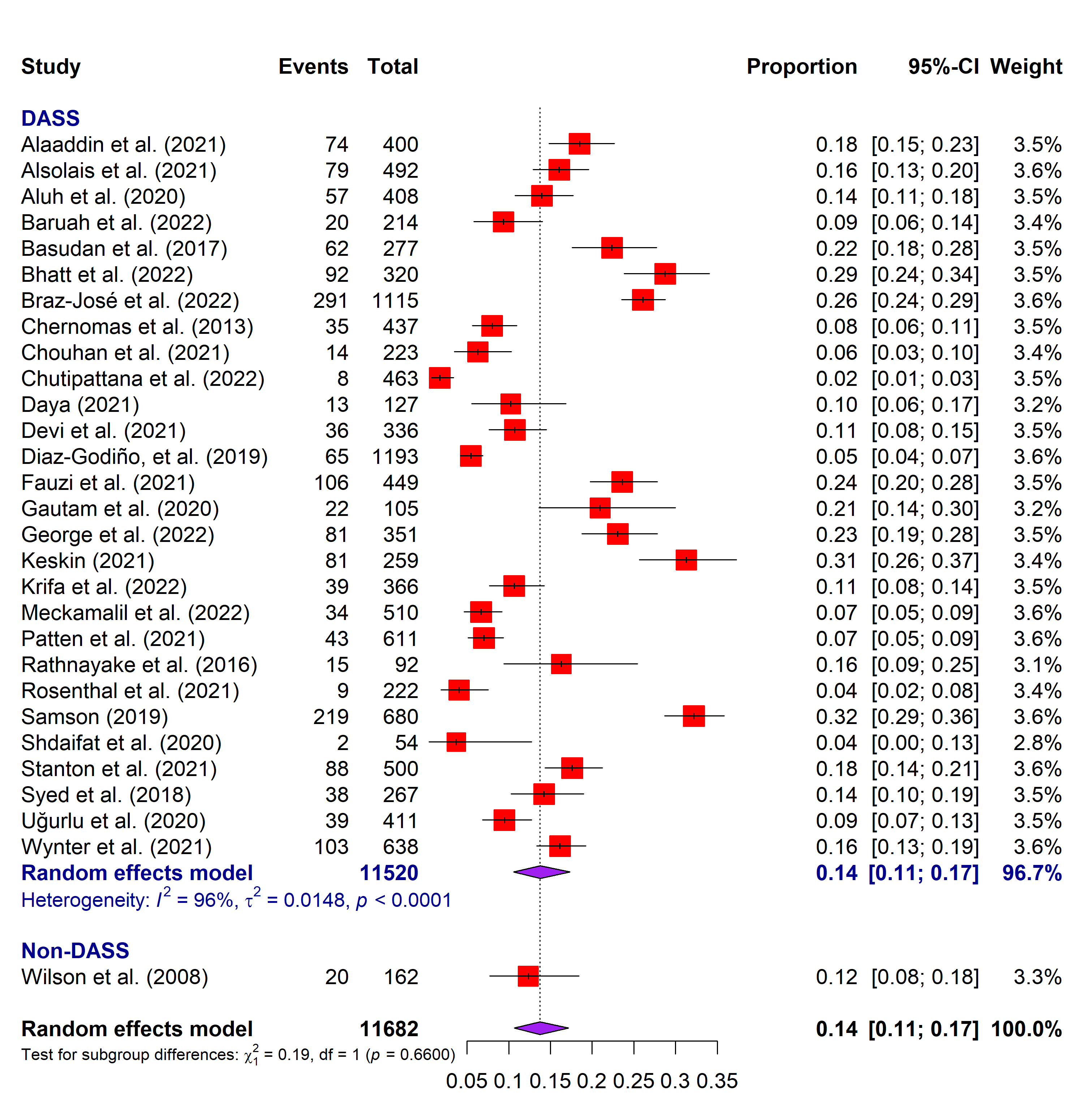
**

**Fig. S24** Subgroup analysis of global prevalence (95% confidence interval) of extremely severe anxiety symptoms by types of measurement.

**
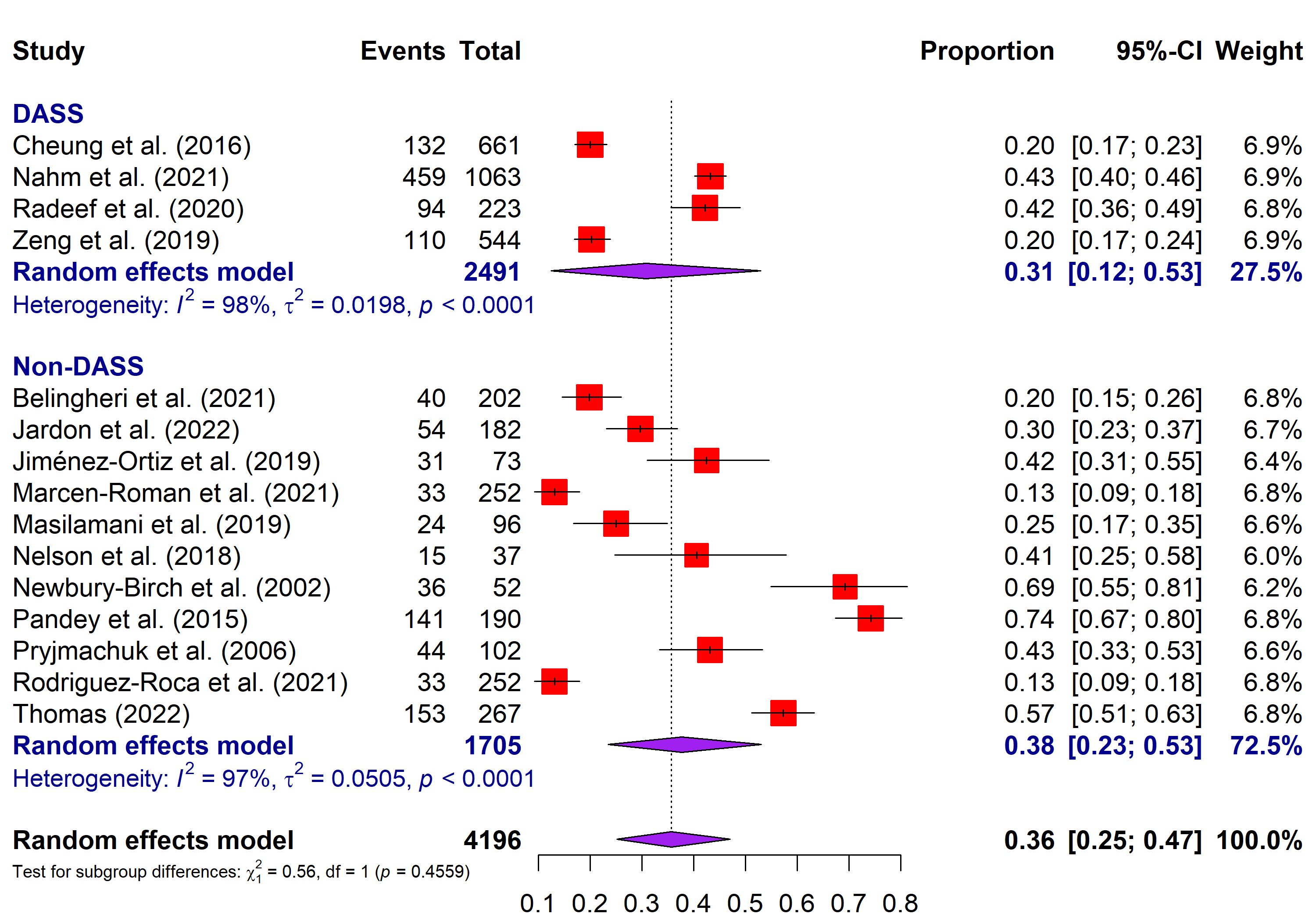
**

**Fig. S25** Subgroup analysis of global prevalence (95% confidence interval) of unspecific stress symptoms by types of measurement.

**
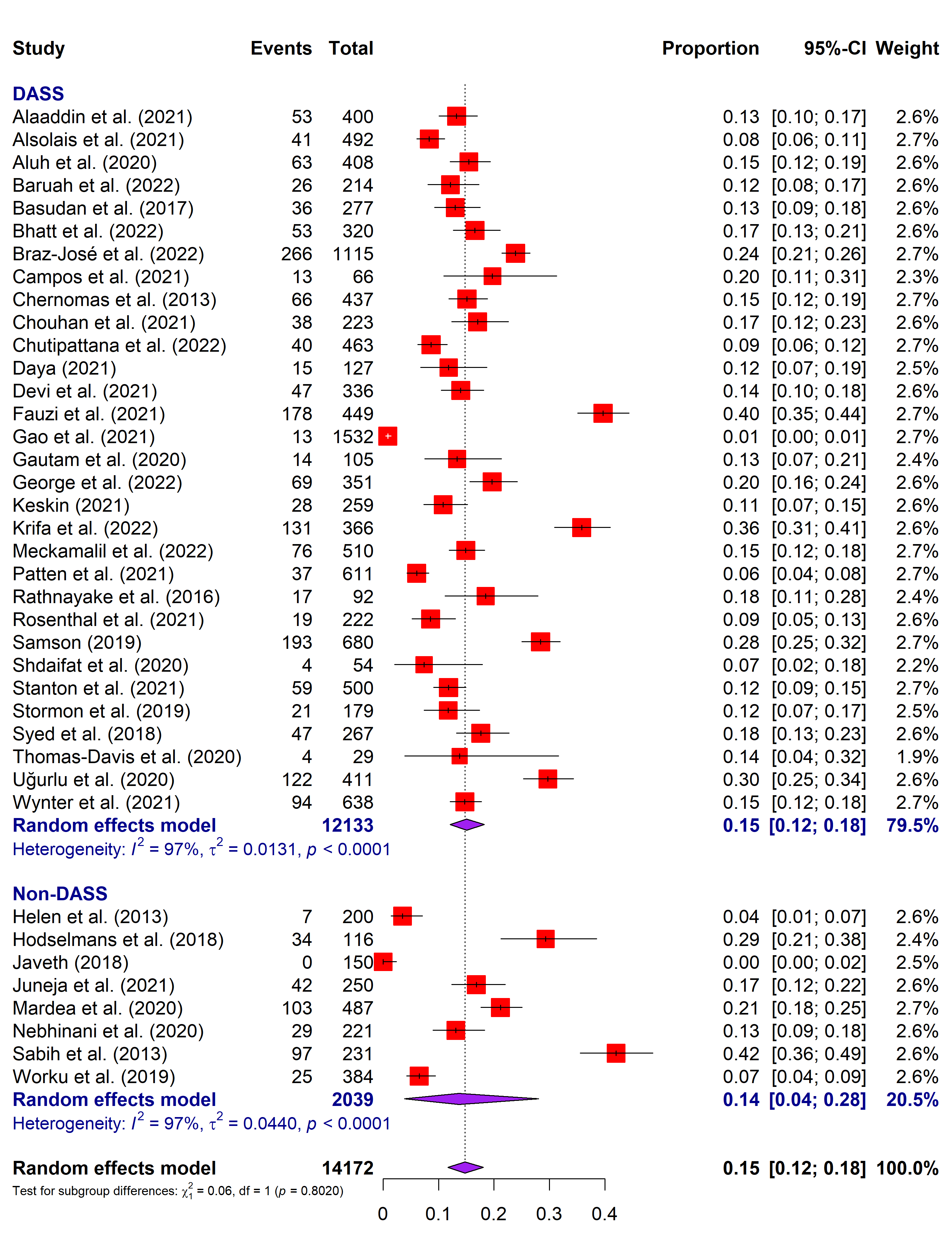
**

**Fig. S26** Subgroup analysis of global prevalence (95% confidence interval) of mild stress symptoms by types of measurement.

**
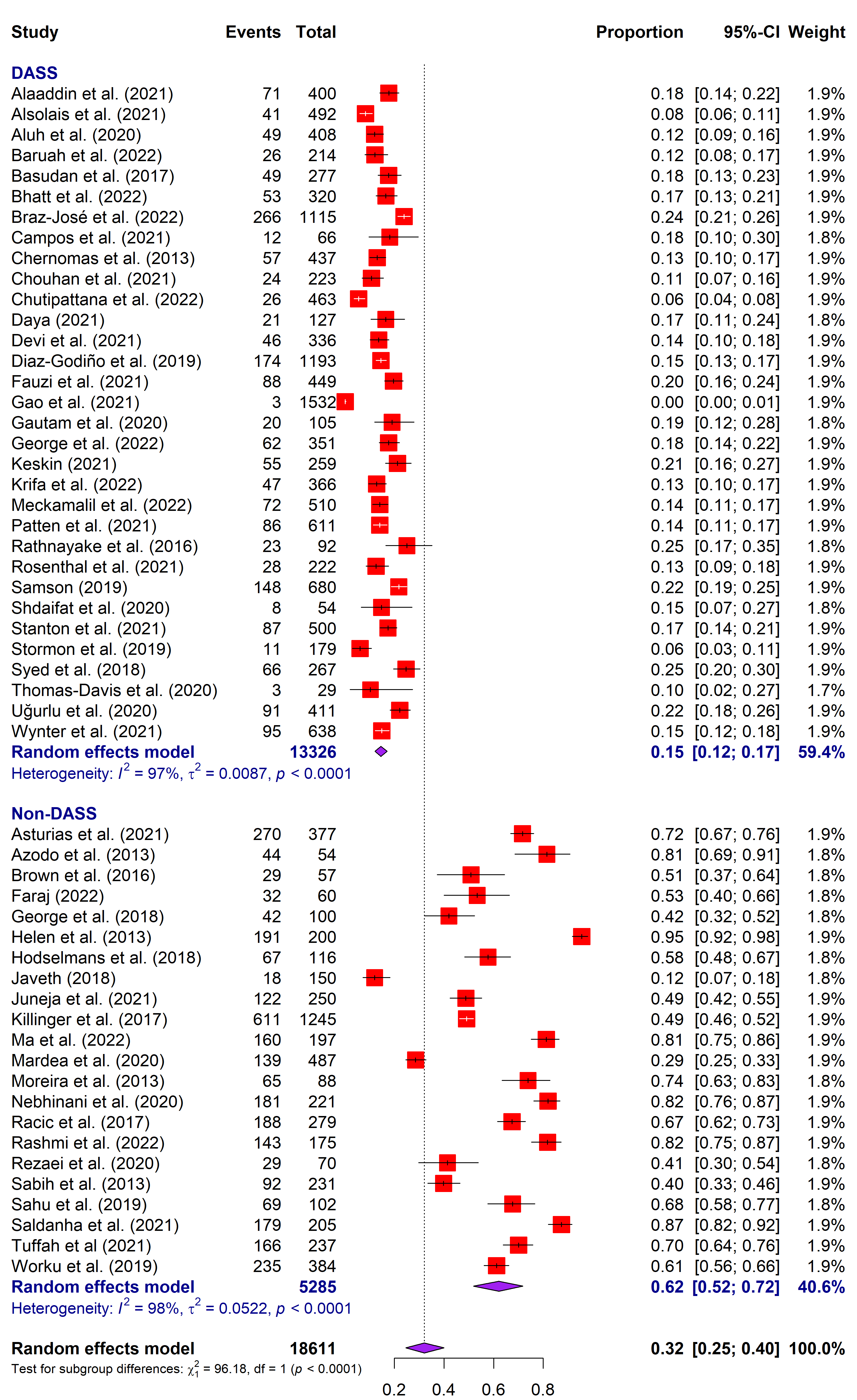
**

**Fig. S27** Subgroup analysis of global prevalence (95% confidence interval) of moderate stress symptoms by types of measurement.

**
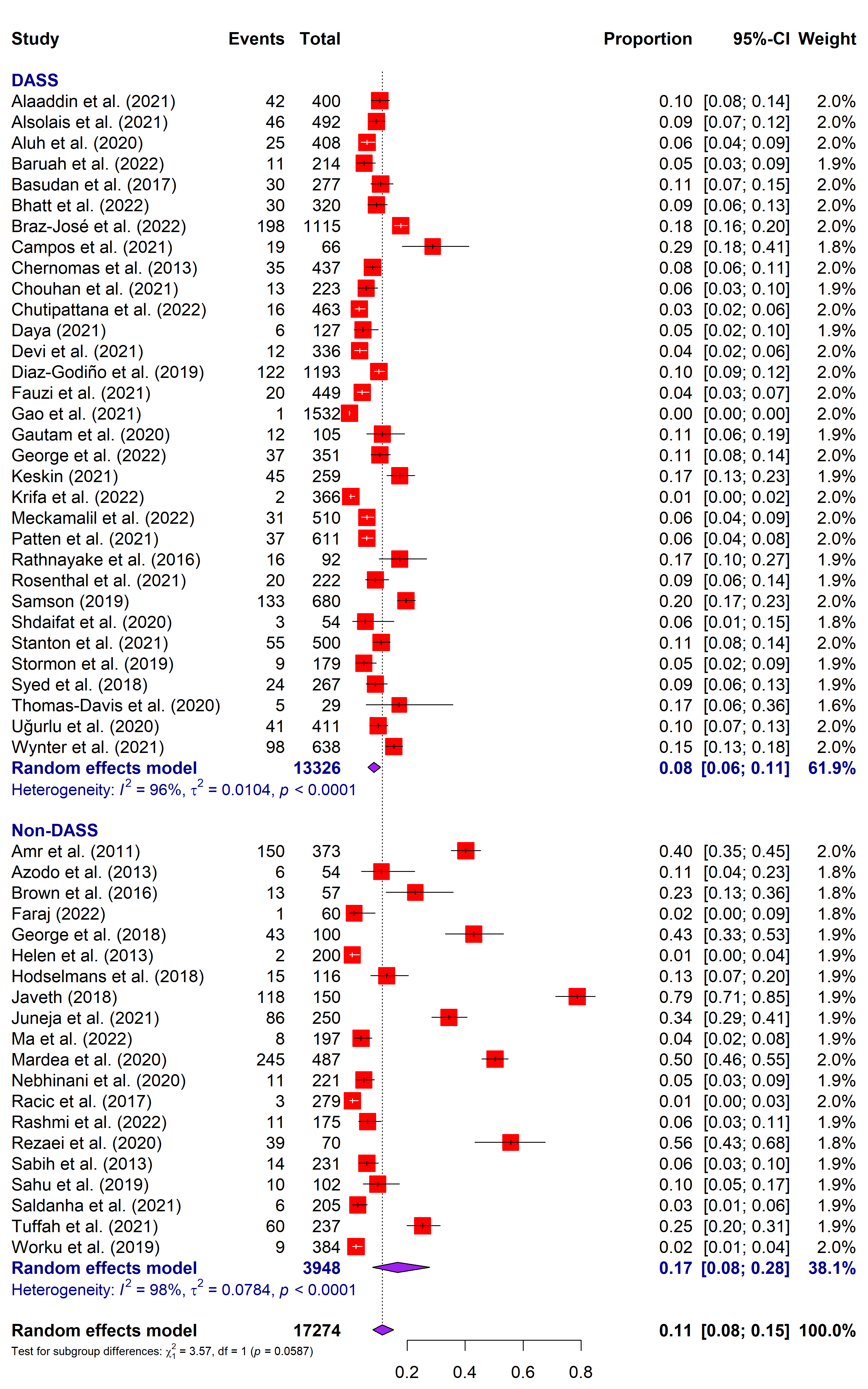
**

**Fig. S28** Subgroup analysis of global prevalence (95% confidence interval) of severe stress symptoms by types of measurement.

**
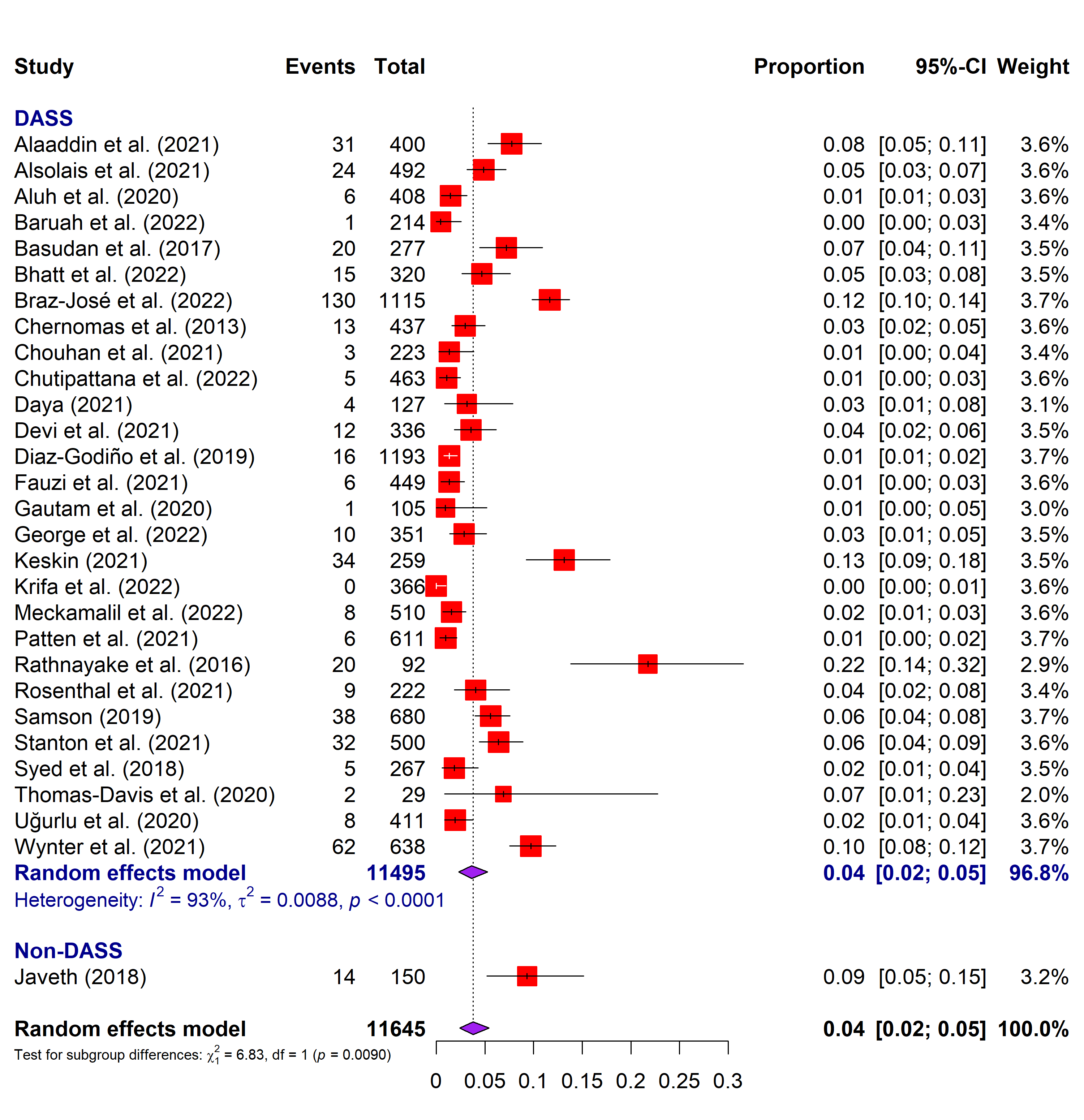
**

**Fig. S29** Subgroup analysis of global prevalence (95% confidence interval) of extremely severe stress symptoms by types of measurement.

**
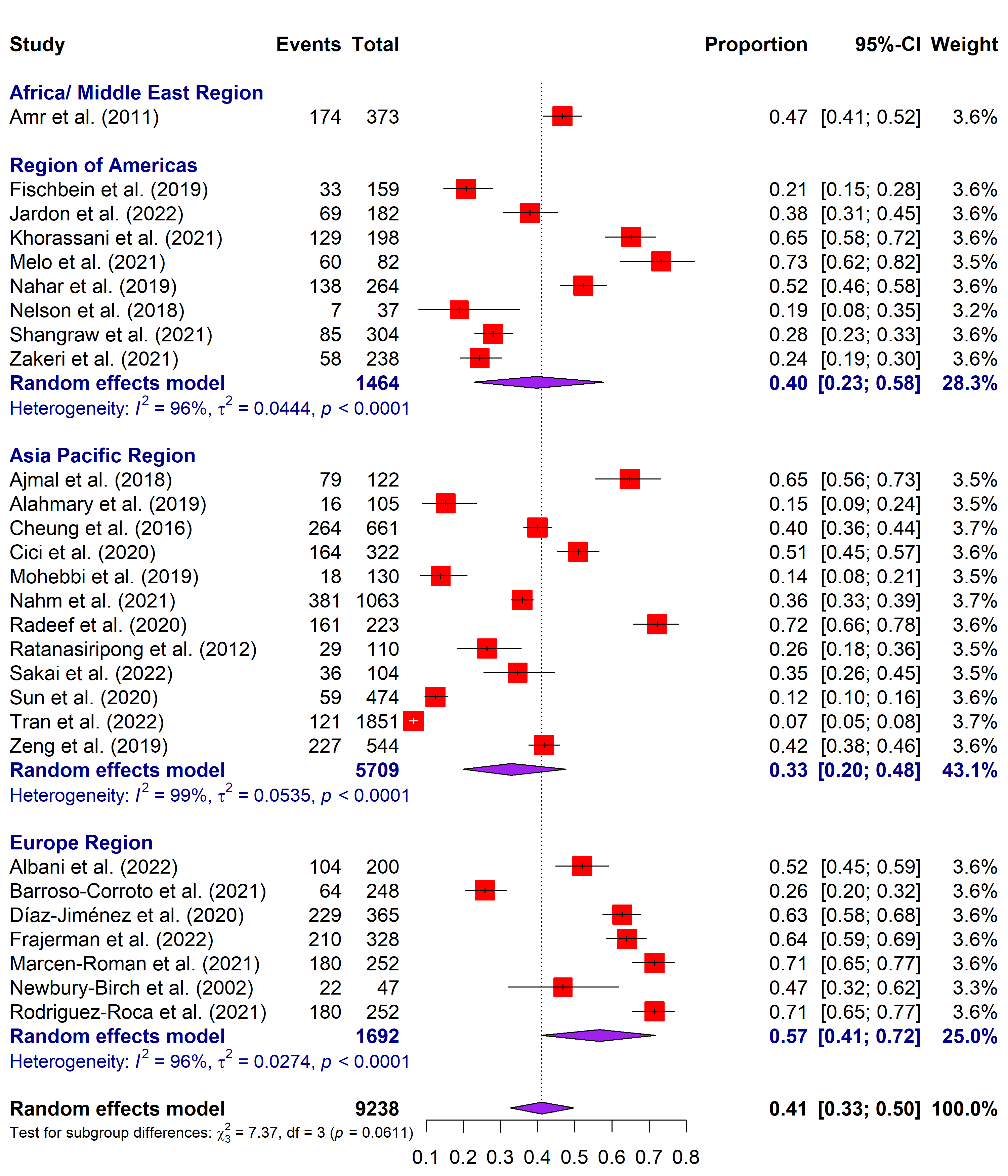
**

**Fig. S30** Subgroup analysis of global prevalence (95% confidence interval) of unspecific anxiety symptoms by geographical regions.

**
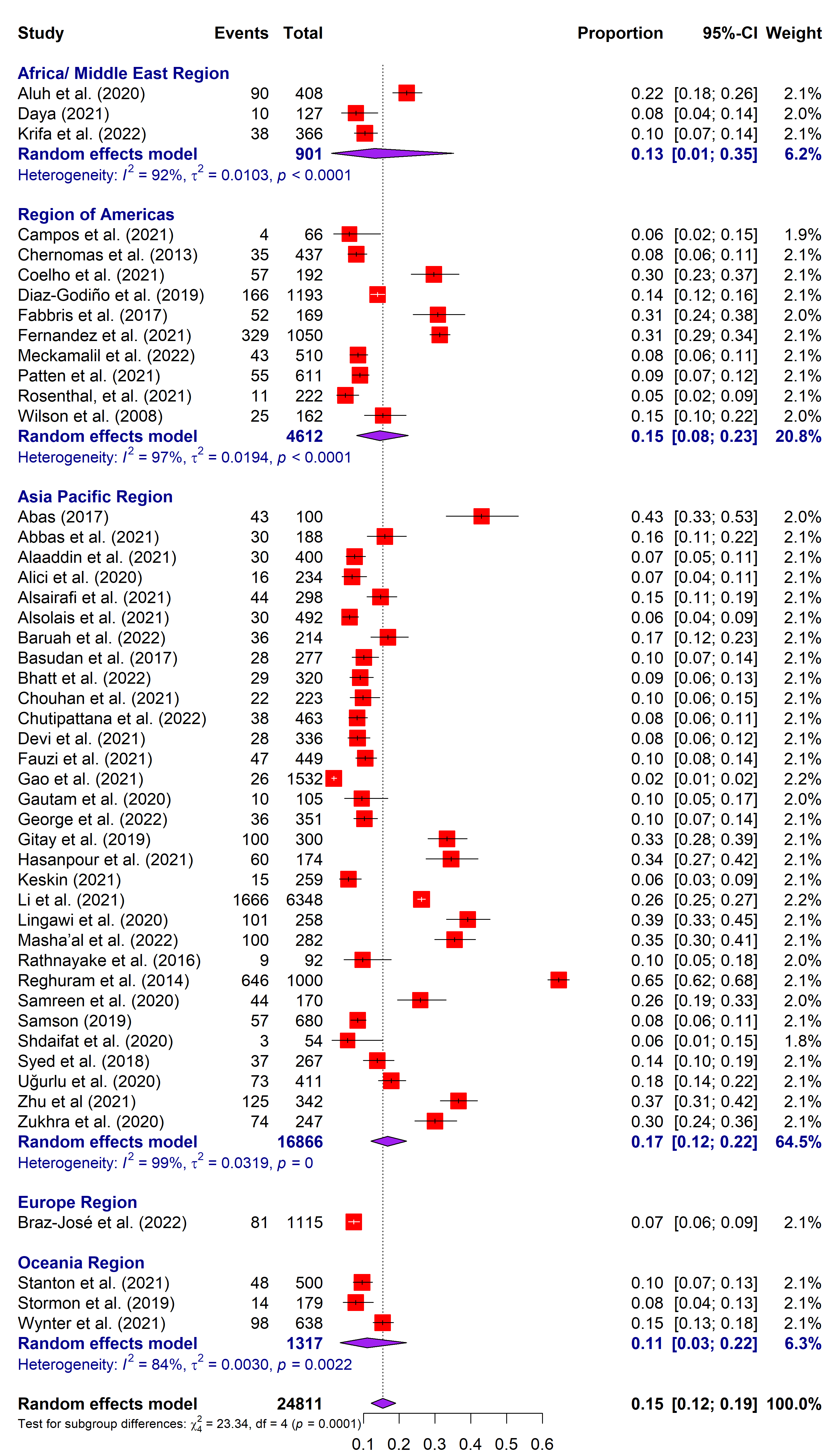
**

**Fig. S31** Subgroup analysis of global prevalence (95% confidence interval) of mild anxiety symptoms by geographical regions.

**
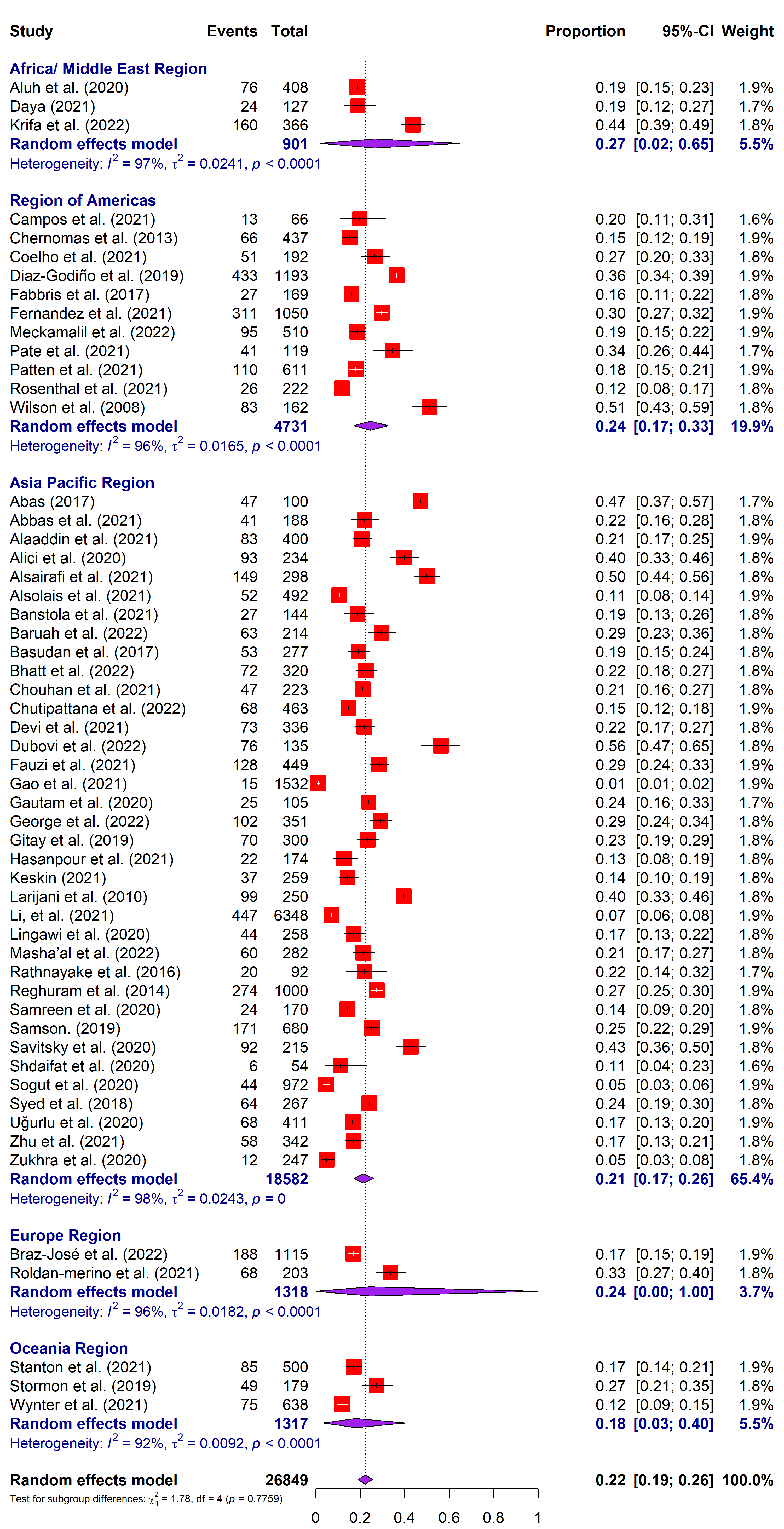
**

**Fig. S32** Subgroup analysis of global prevalence (95% confidence interval) of moderate anxiety symptoms by geographical regions.

**
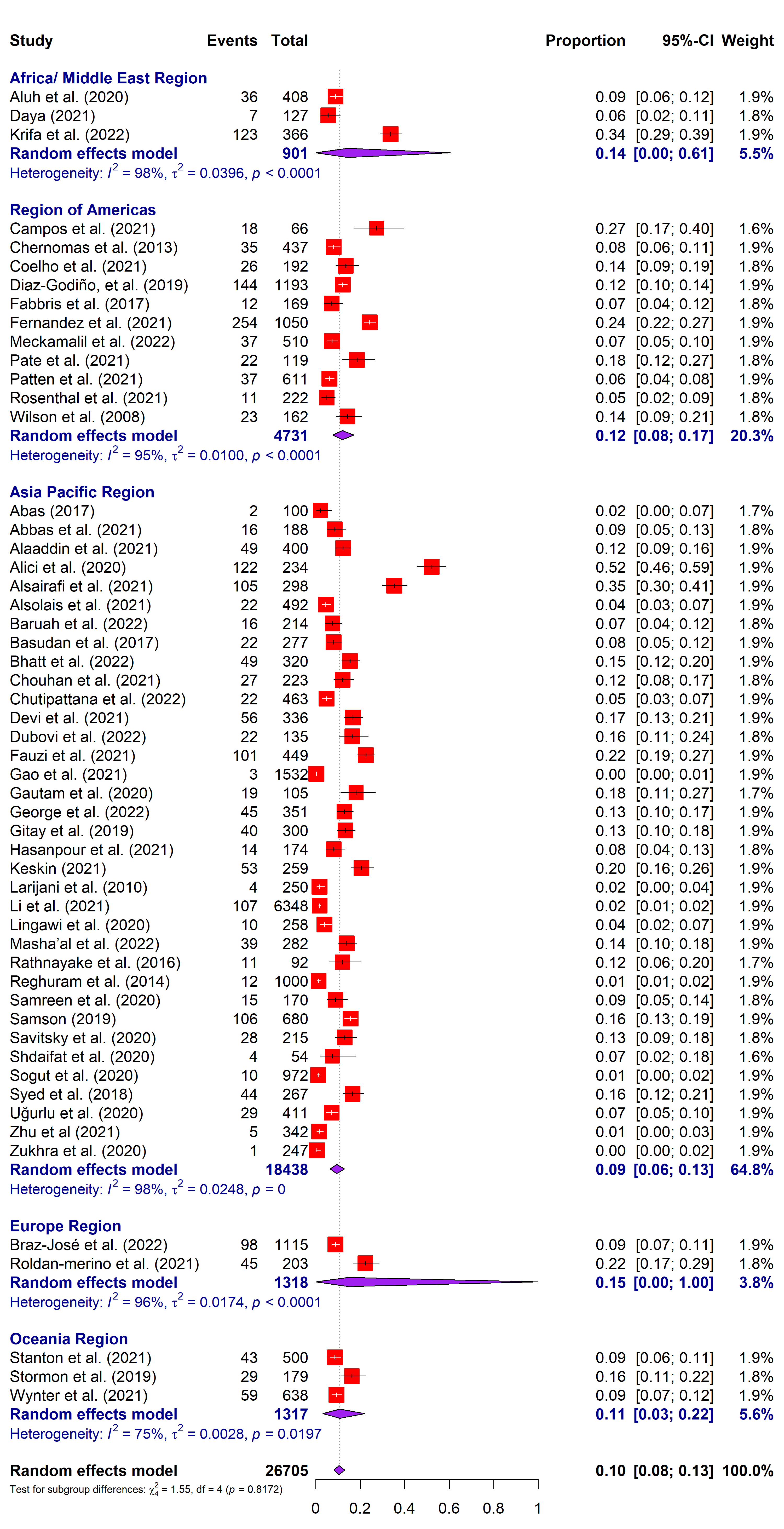
**

**Fig. S33** Subgroup analysis of global prevalence (95% confidence interval) of severe anxiety symptoms by geographical regions.

**
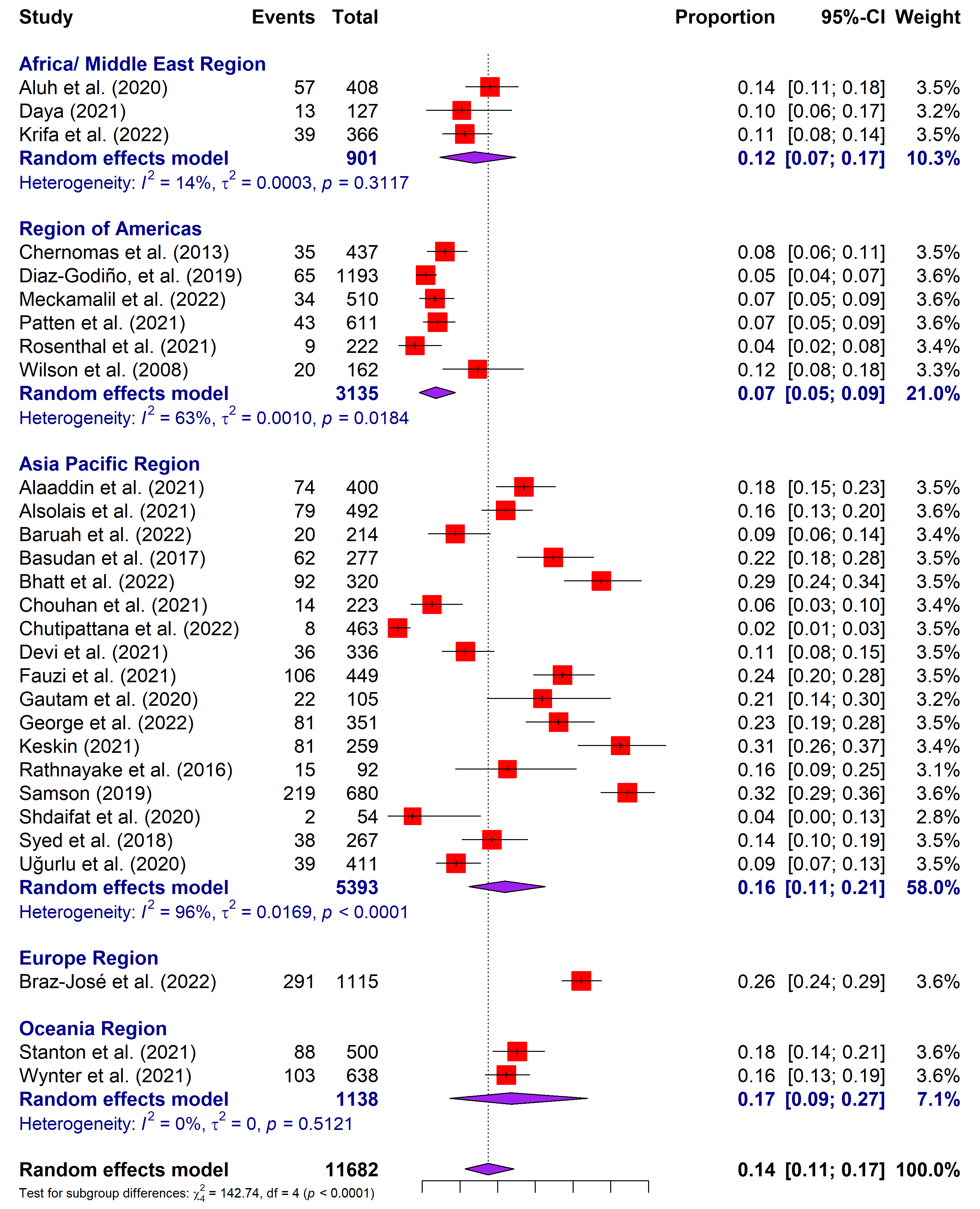
**

**Fig. S34** Subgroup analysis of global prevalence (95% confidence interval) of extremely severe anxiety symptoms by geographical regions.

**
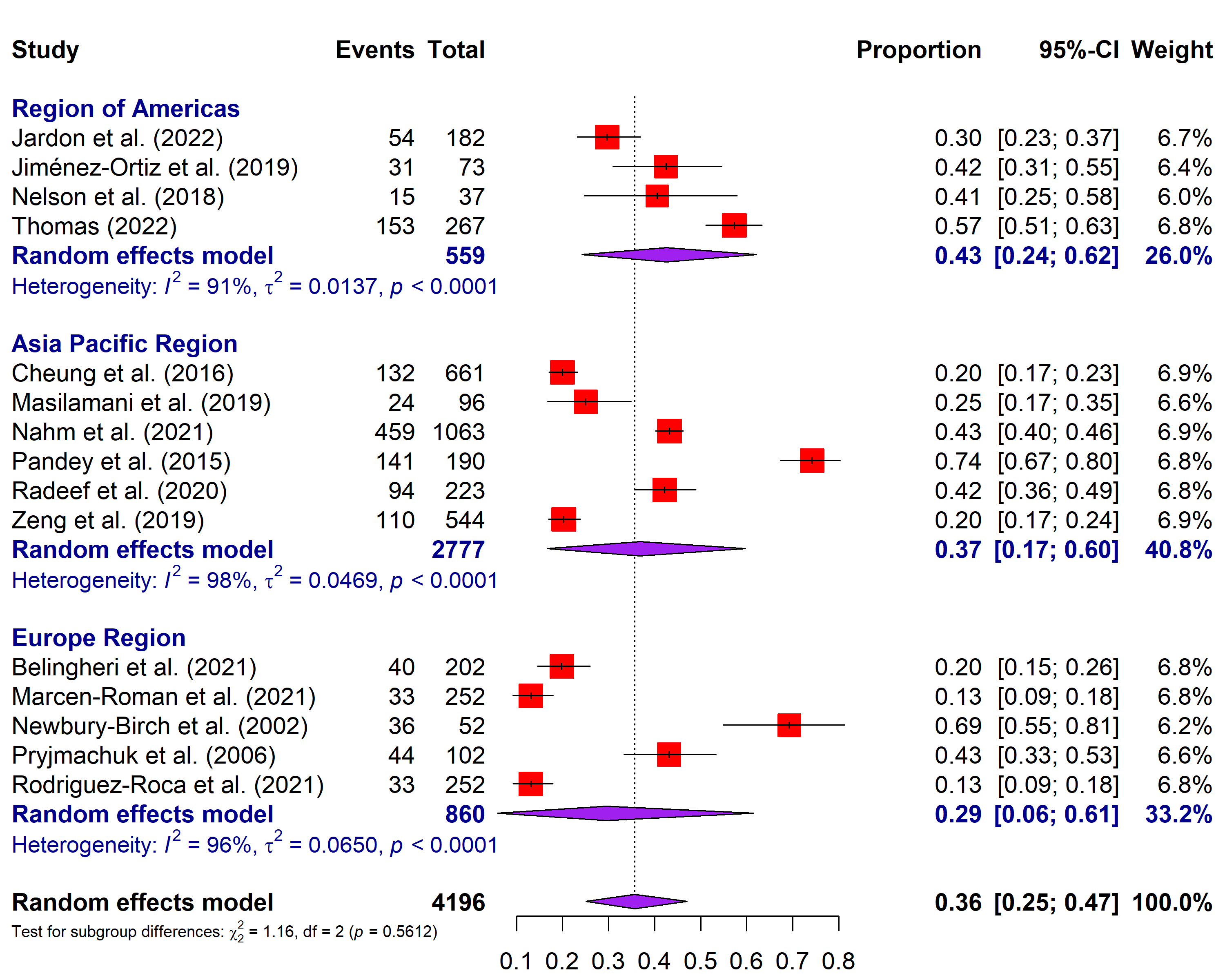
**

**Fig. S35** Subgroup analysis of global prevalence (95% confidence interval) of unspecified stress symptoms by geographical regions.

**
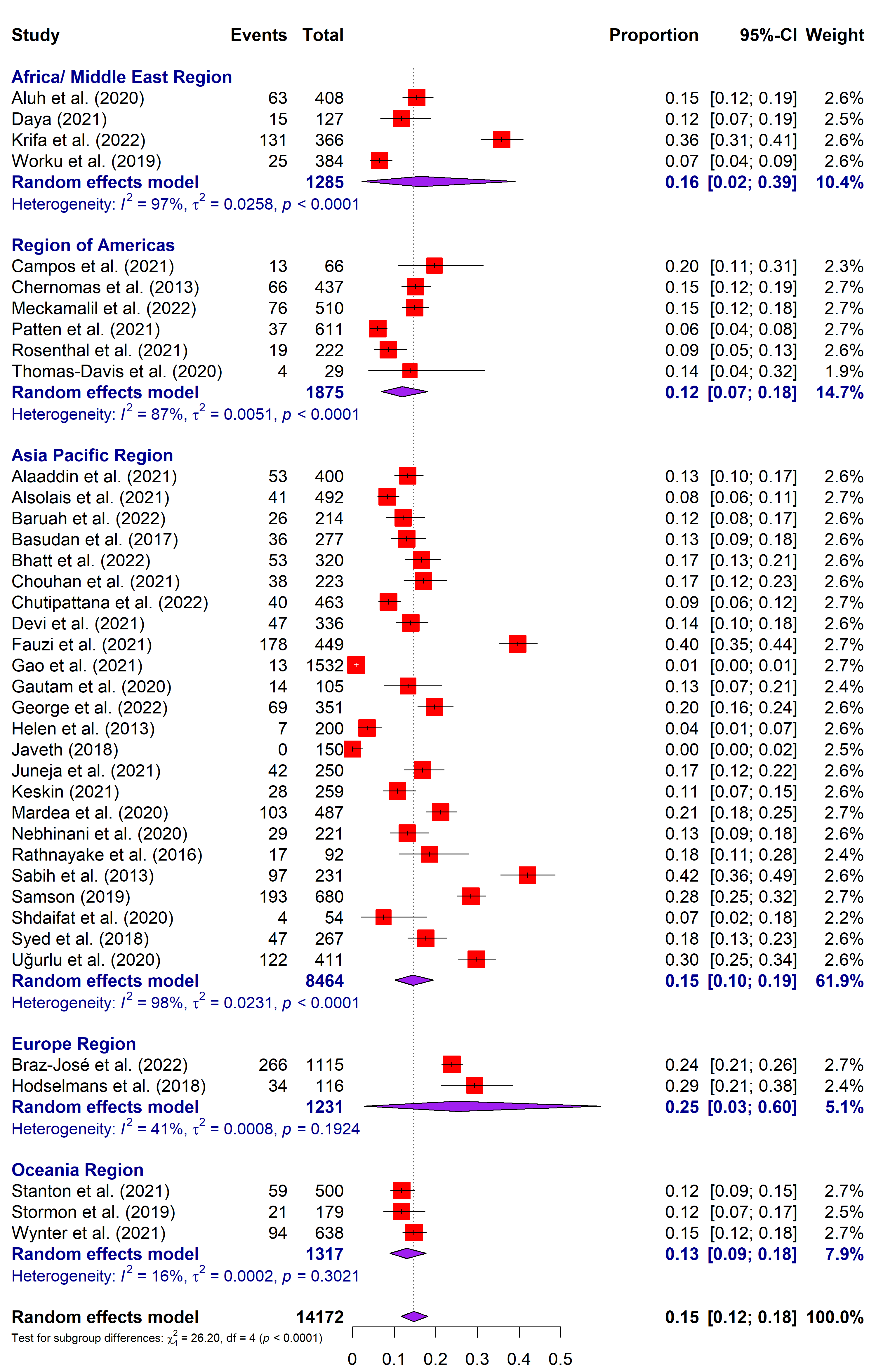
**

**Fig. S36** Subgroup analysis of global prevalence (95% confidence interval) of mild stress symptoms by geographical regions.

**
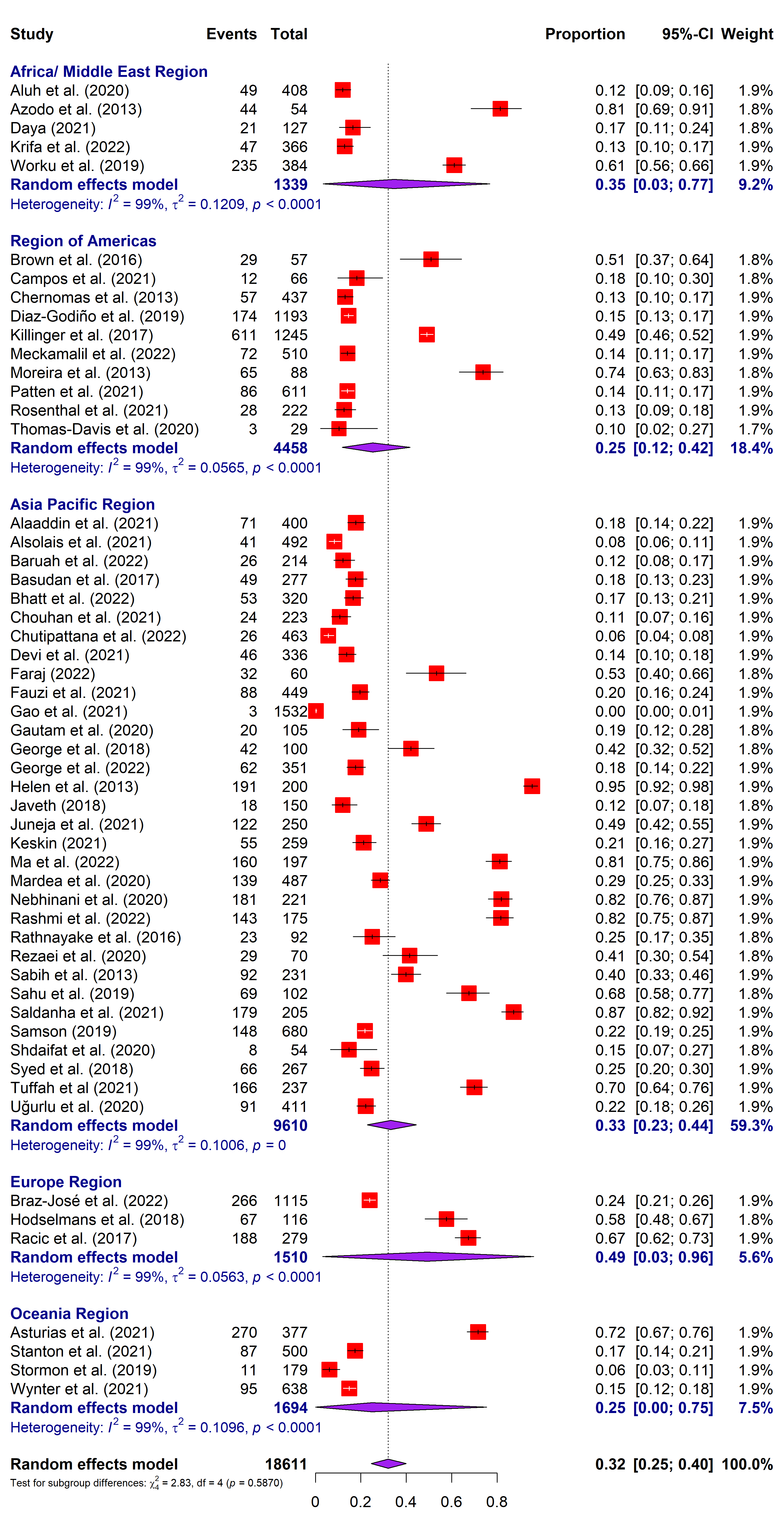
**

**Fig. S37** Subgroup analysis of global prevalence (95% confidence interval) of moderate stress symptoms by geographical regions.

**
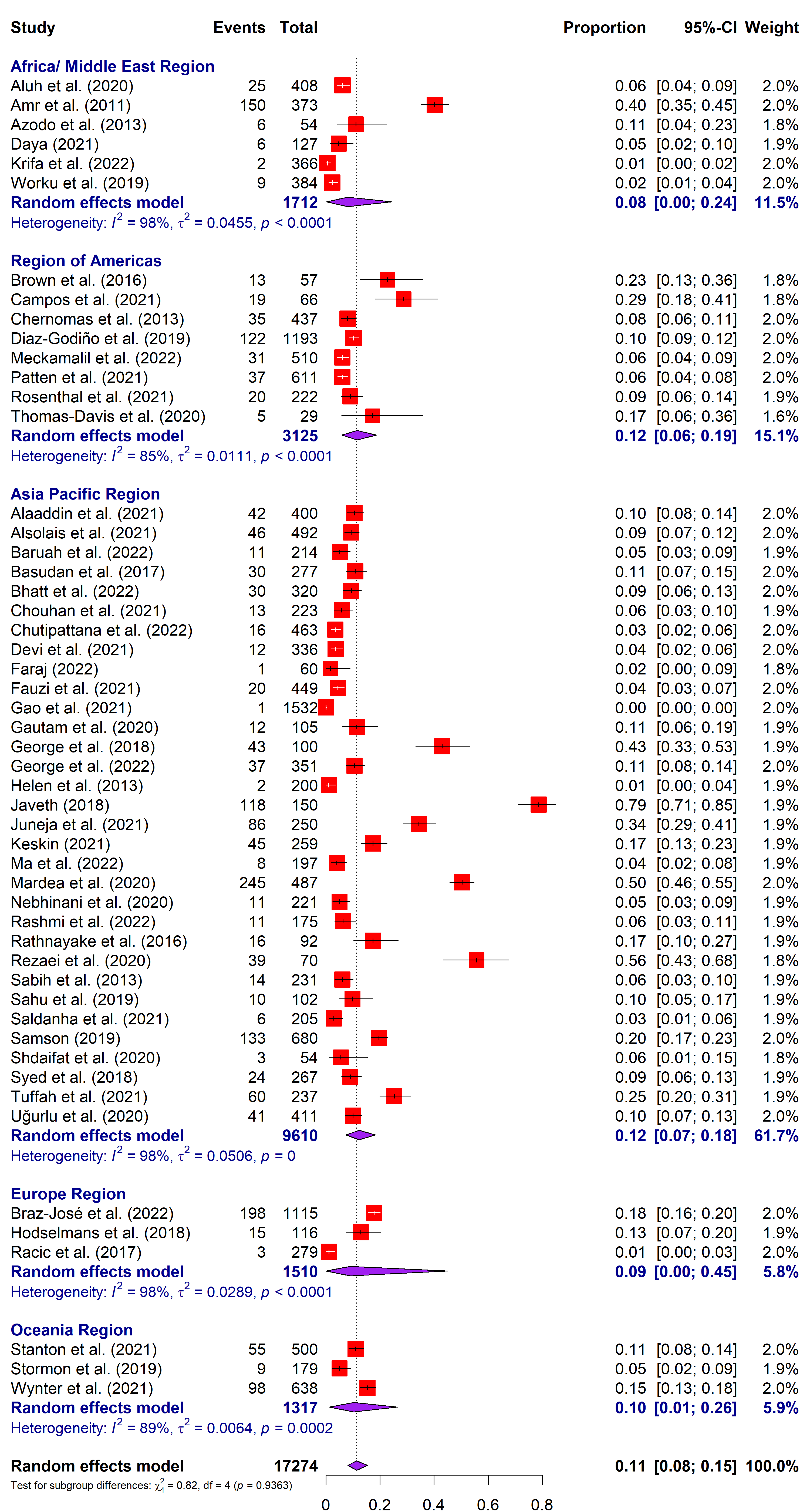
**

**Fig. S38** Subgroup analysis of global prevalence (95% confidence interval) of severe stress symptoms by geographical regions.

**
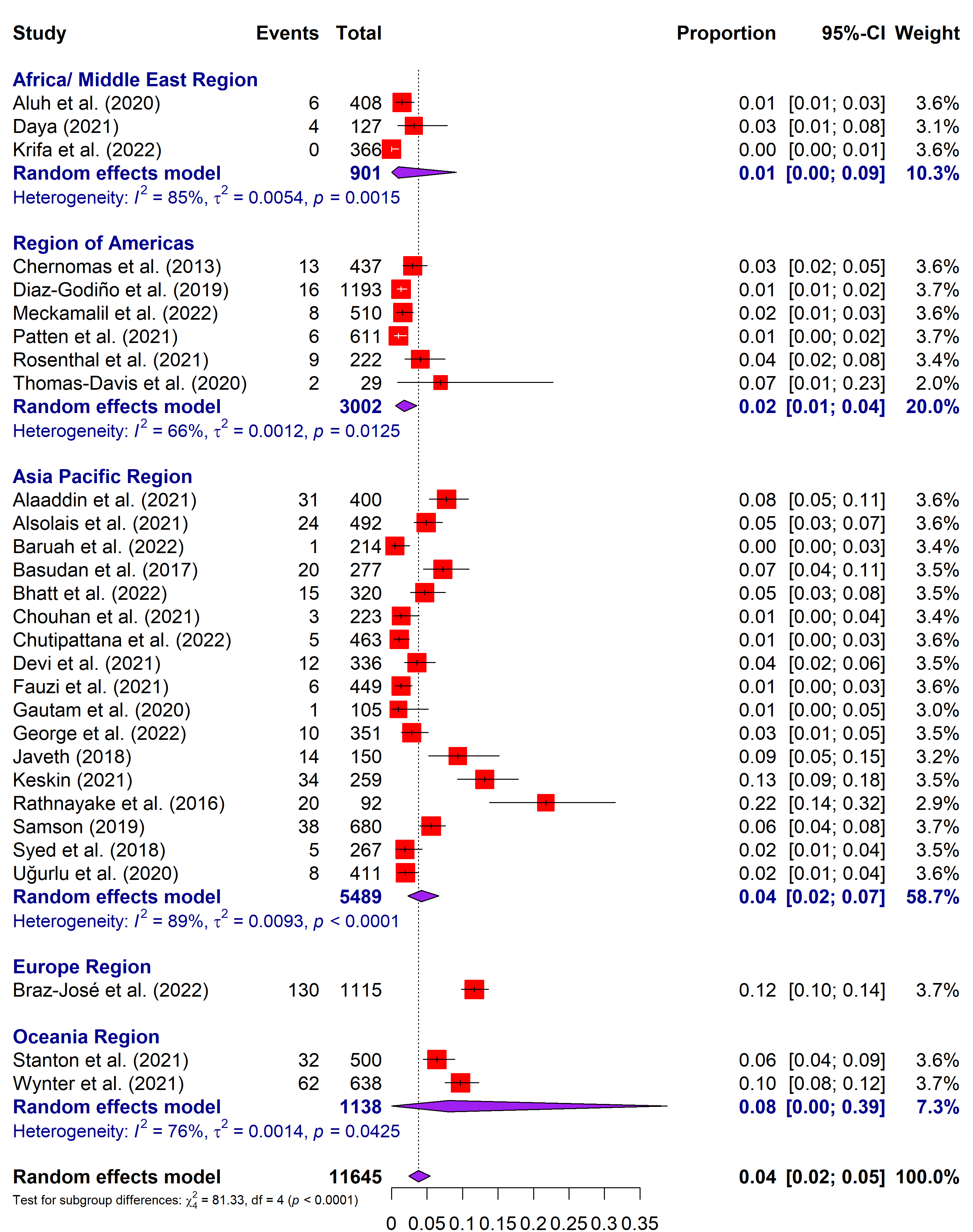
**

**Fig. S39** Subgroup analysis of global prevalence (95% confidence interval) of extremely severe stress symptoms by geographical regions.

**
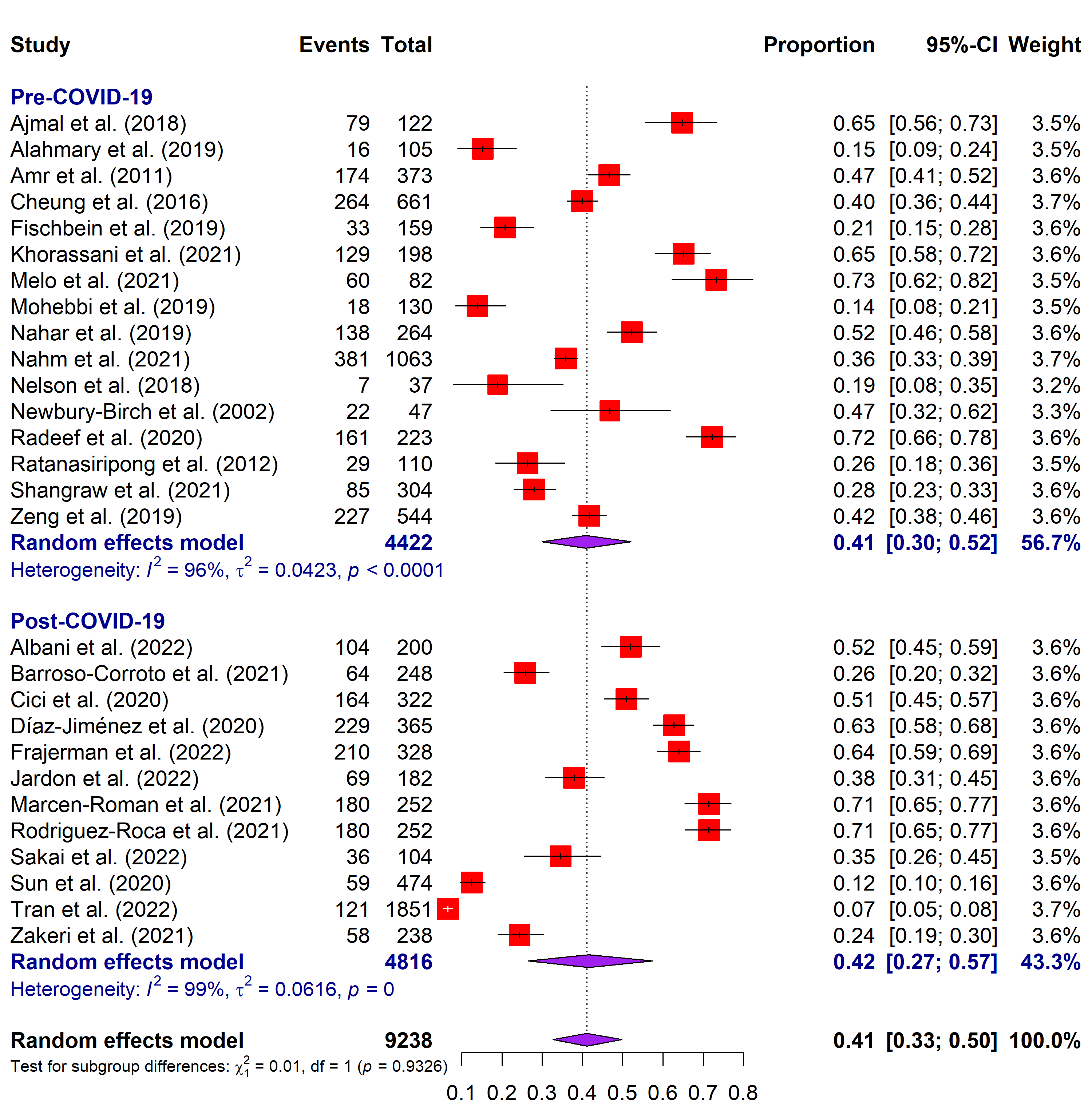
**

**Fig. S40** Subgroup analysis of global prevalence (95% confidence interval) of unspecific anxiety symptoms by data collection period.

**
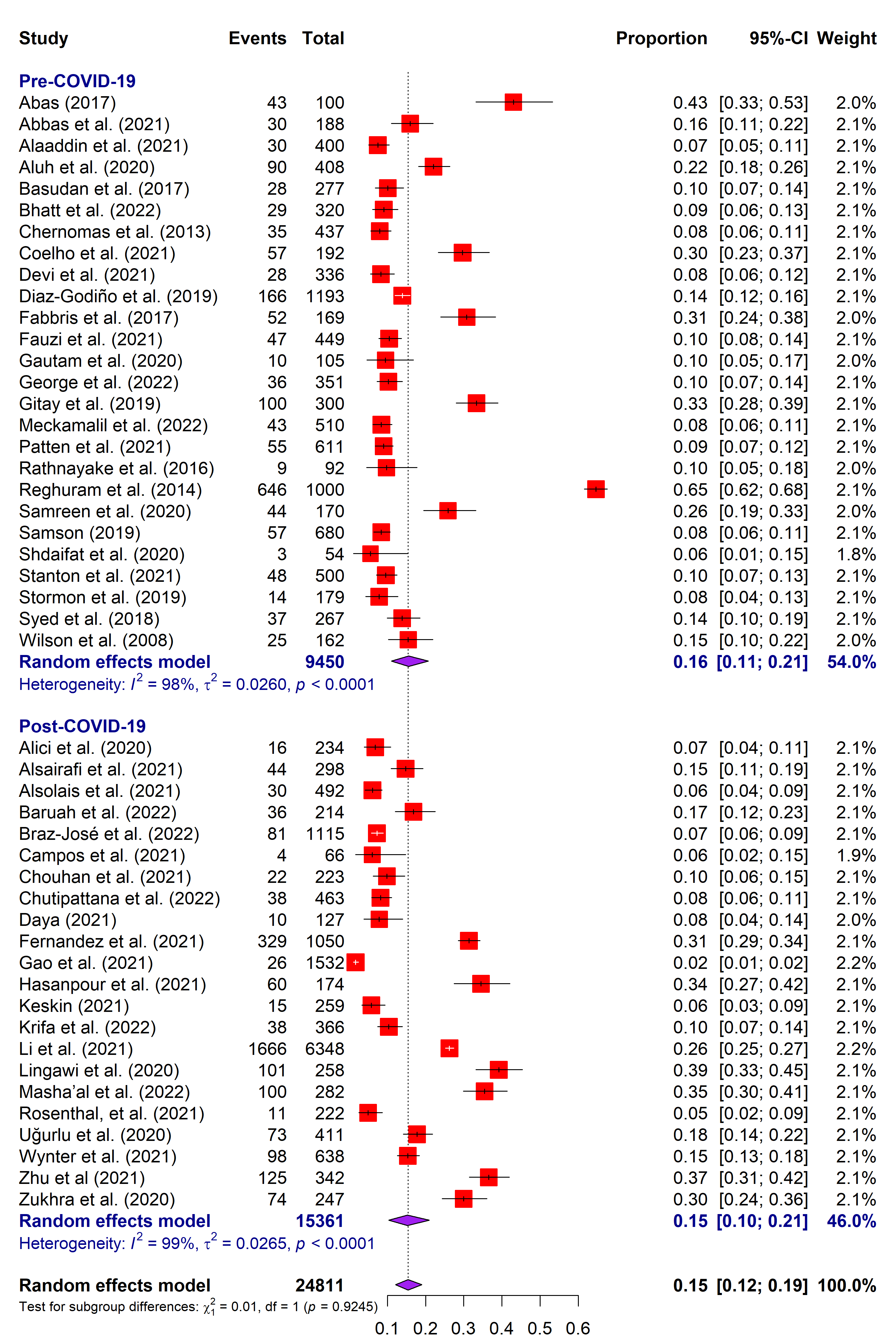
**

**Fig. S41** Subgroup analysis of global prevalence (95% confidence interval) of mild anxiety symptoms by data collection period.

**
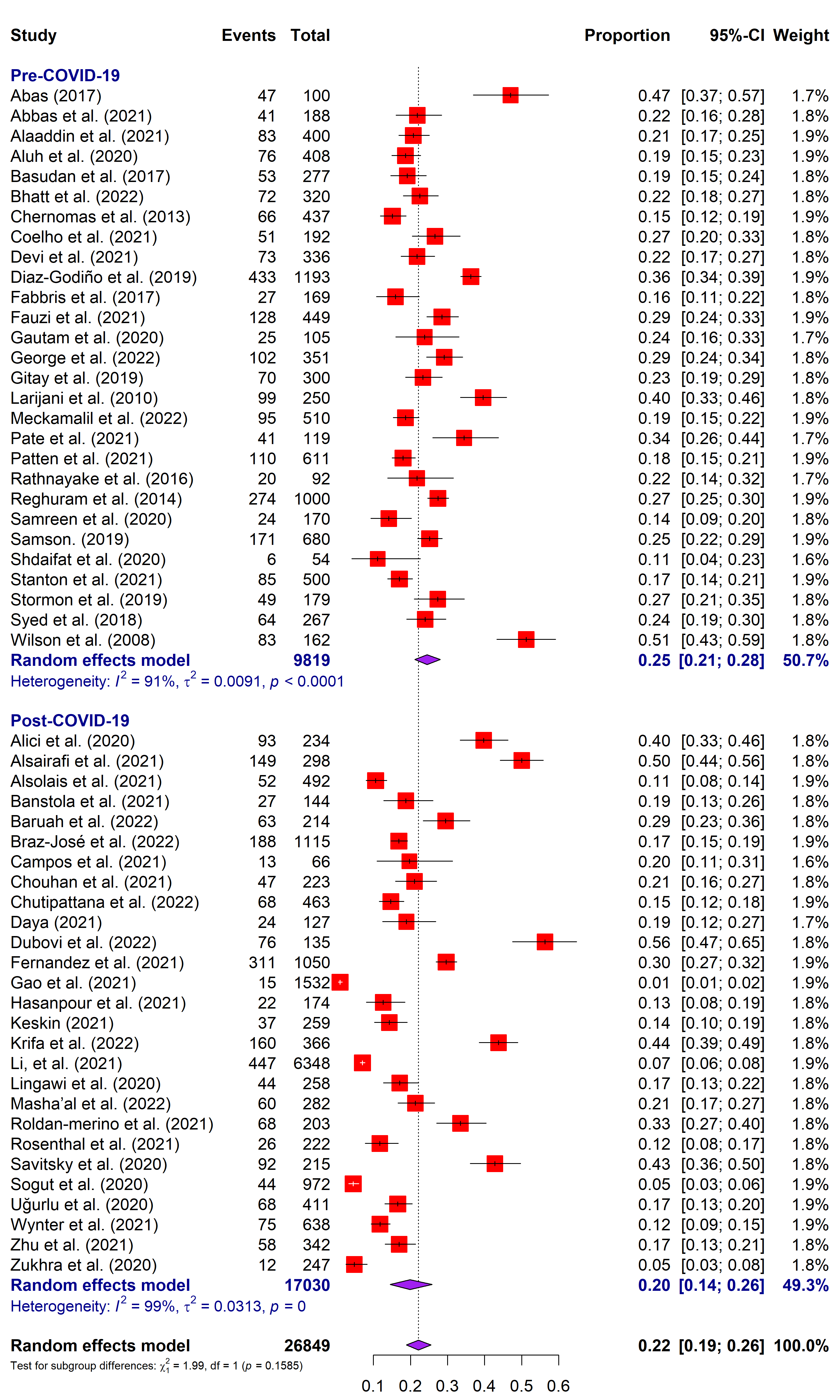
**

**Fig. S42** Subgroup analysis of global prevalence (95% confidence interval) of moderate anxiety symptoms by data collection period.

**
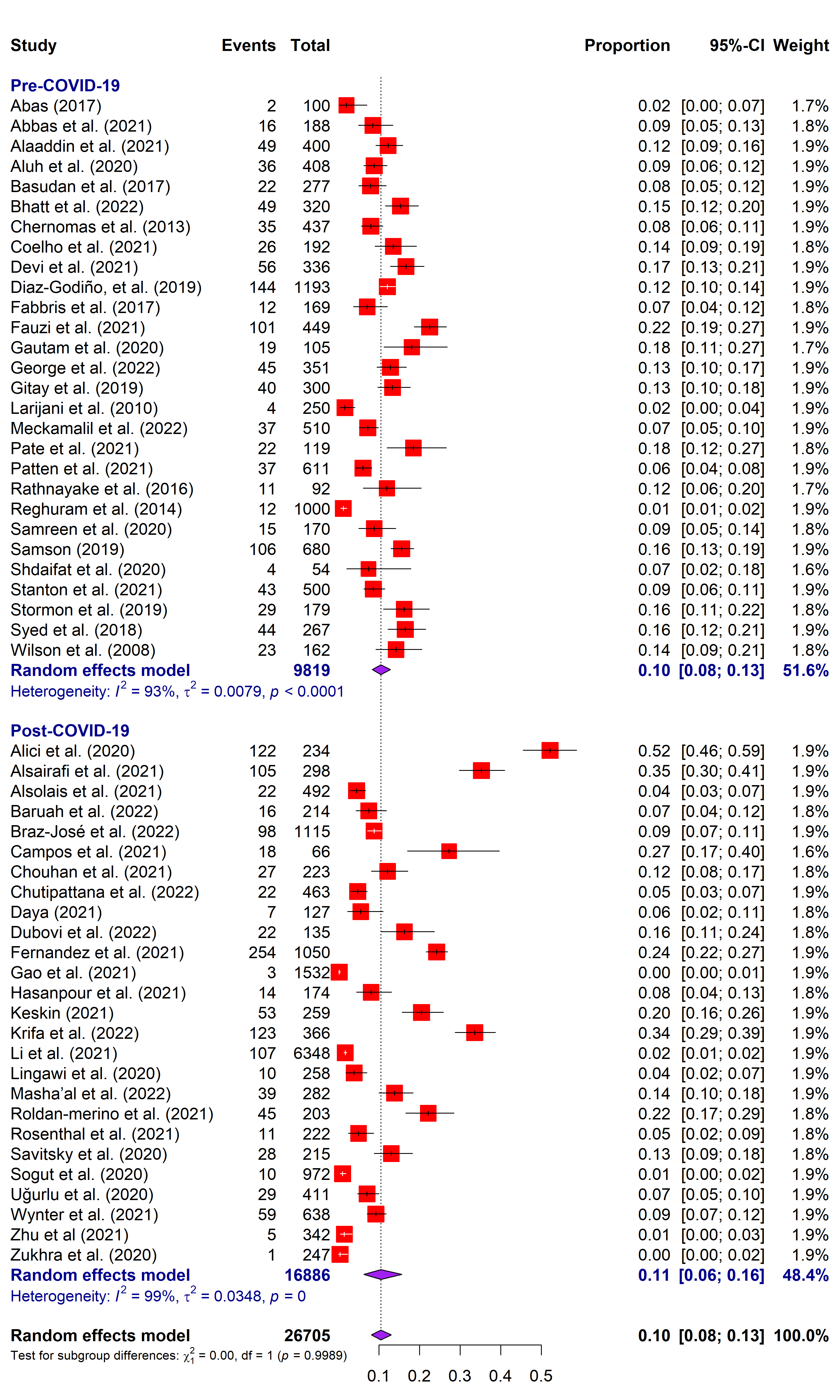
**

**Fig. S43** Subgroup analysis of global prevalence (95% confidence interval) of severe anxiety symptoms by data collection period.

**
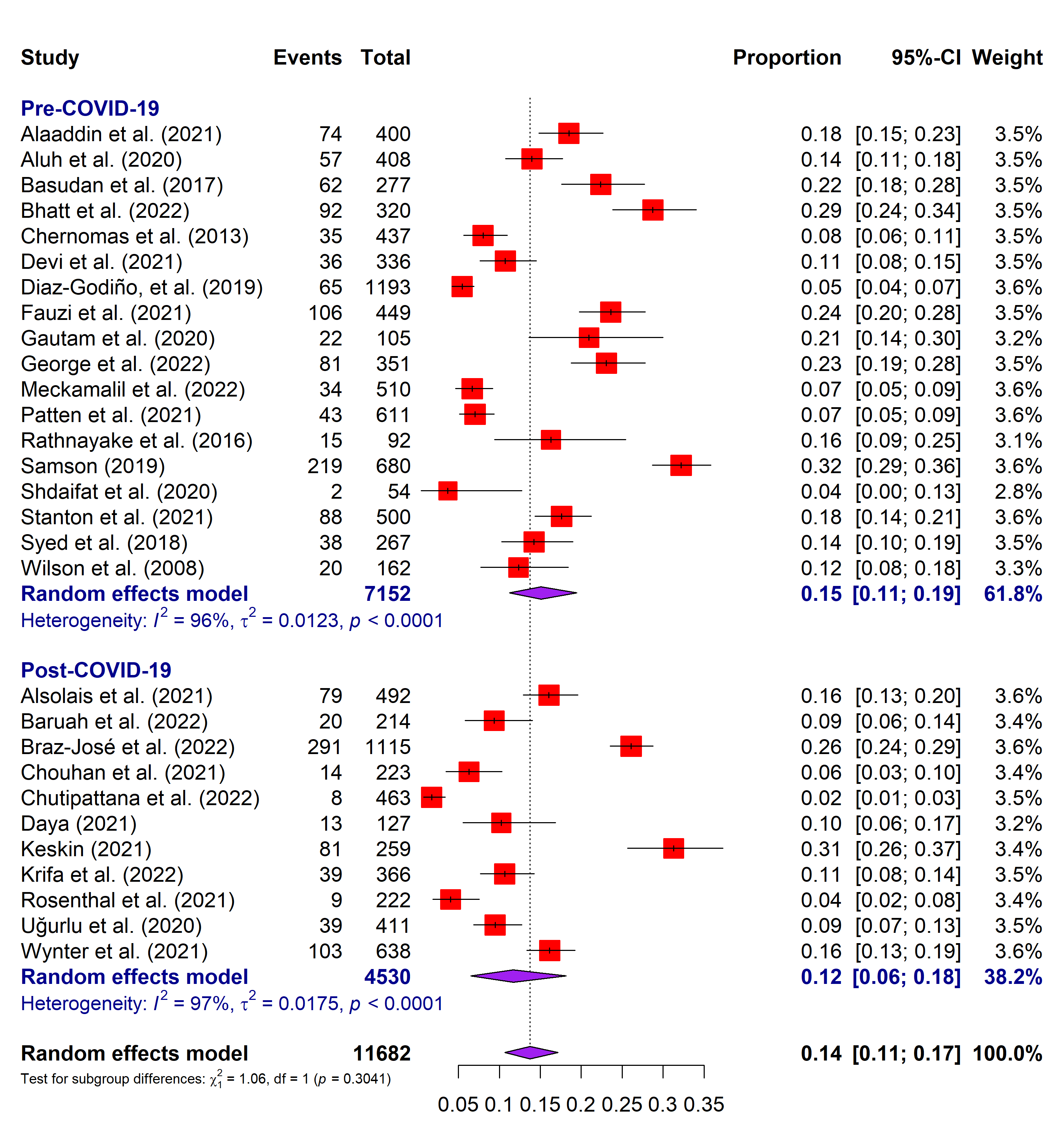
**

**Fig. S44** Subgroup analysis of global prevalence (95% confidence interval) of extremely severe anxiety symptoms by data collection period.

**
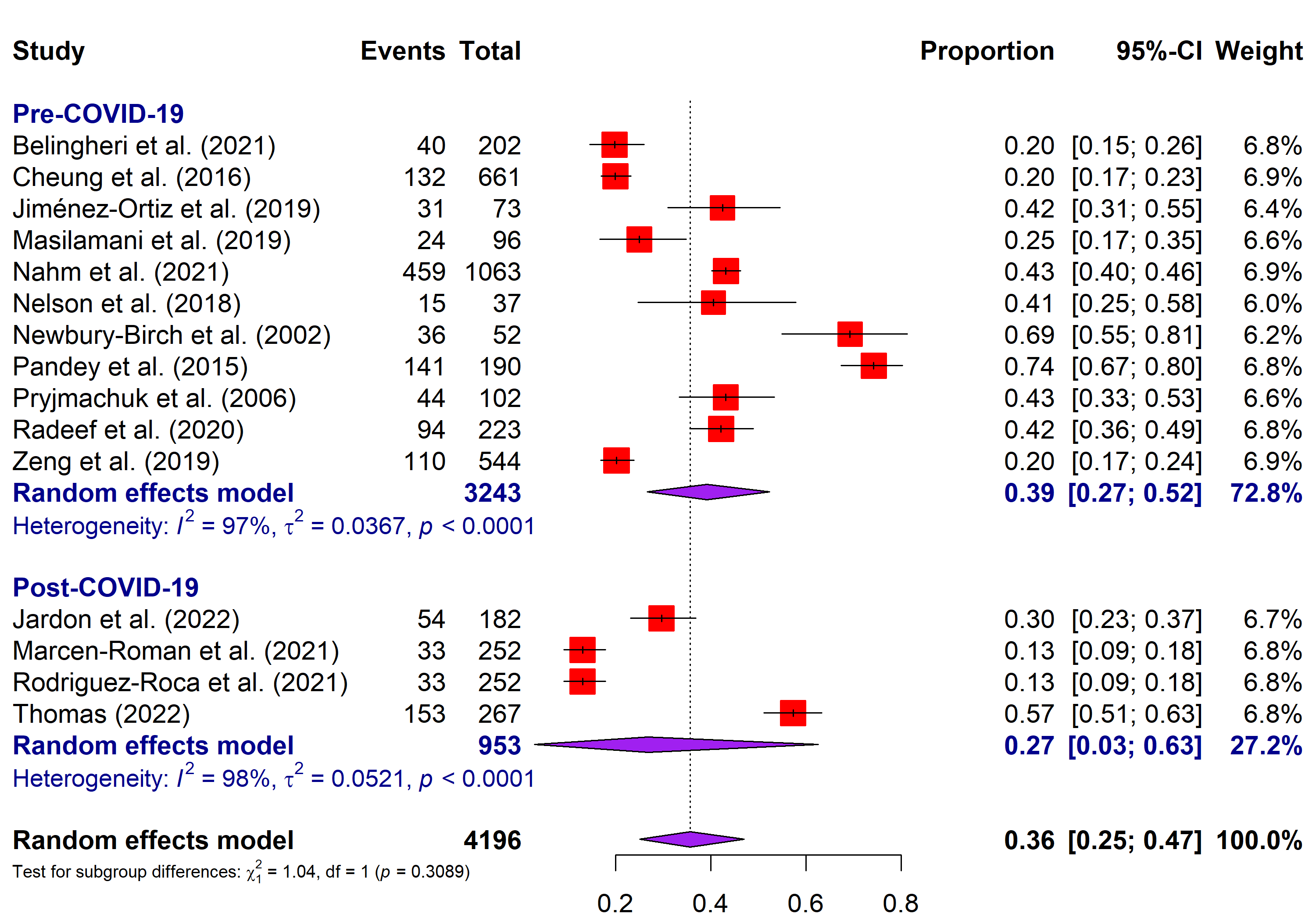
**

**Fig. S45** Subgroup analysis of global prevalence (95% confidence interval) of unspecified stress symptoms by data collection period.

**
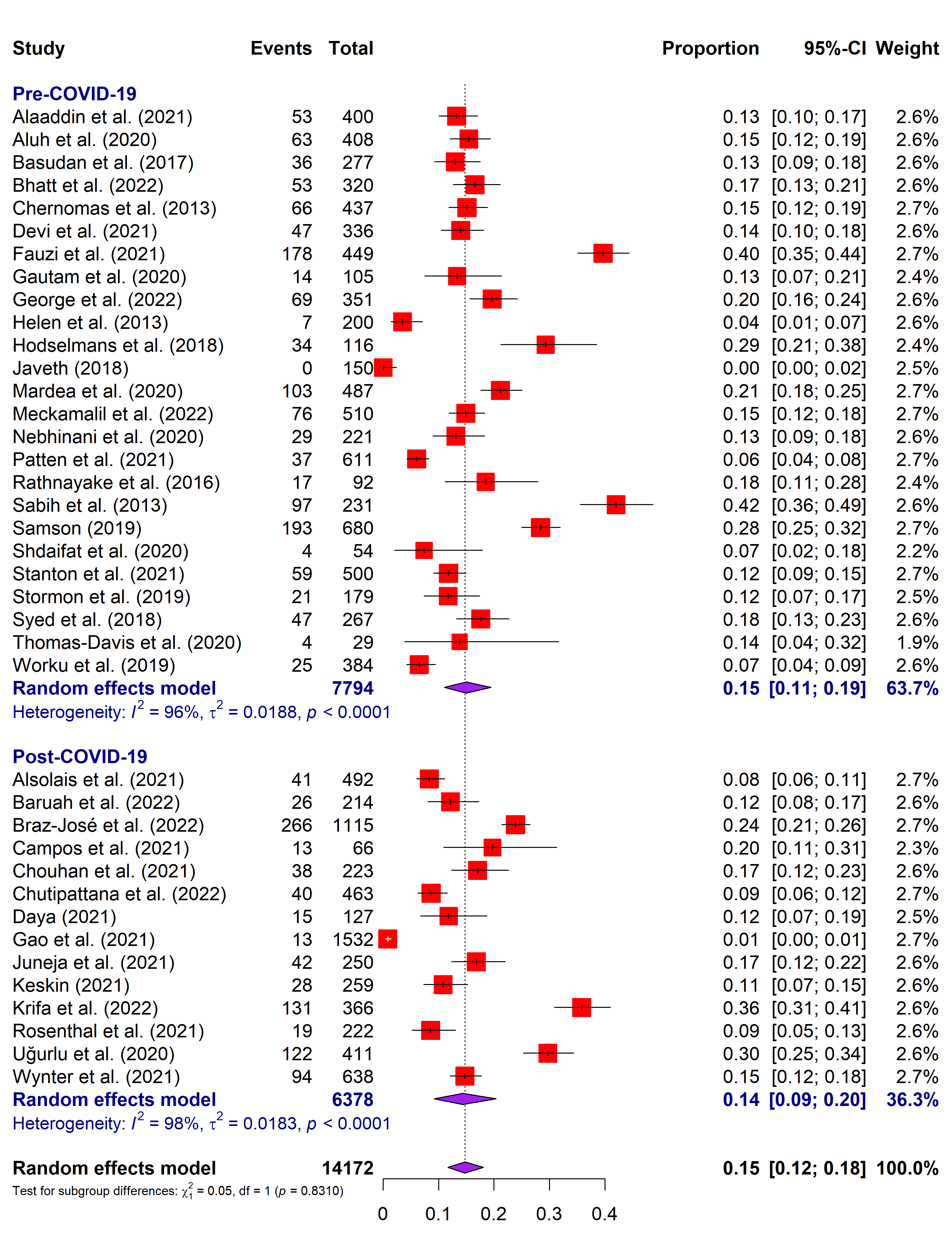
**

**Fig. S46** Subgroup analysis of global prevalence (95% confidence interval) of mild stress symptoms by data collection period.

**
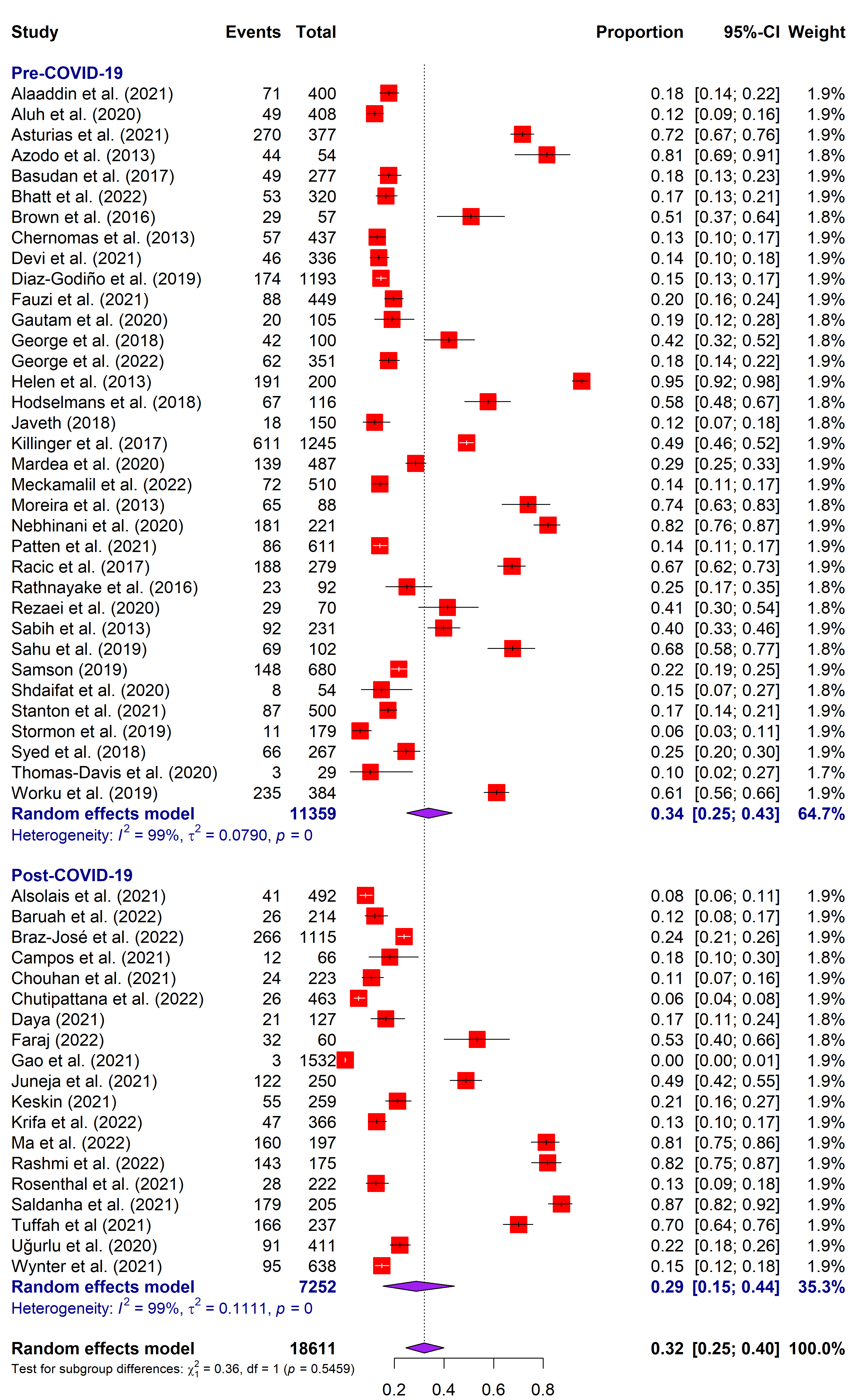
**

**Fig. S47** Subgroup analysis of global prevalence (95% confidence interval) of moderate stress symptoms by data collection period.

**
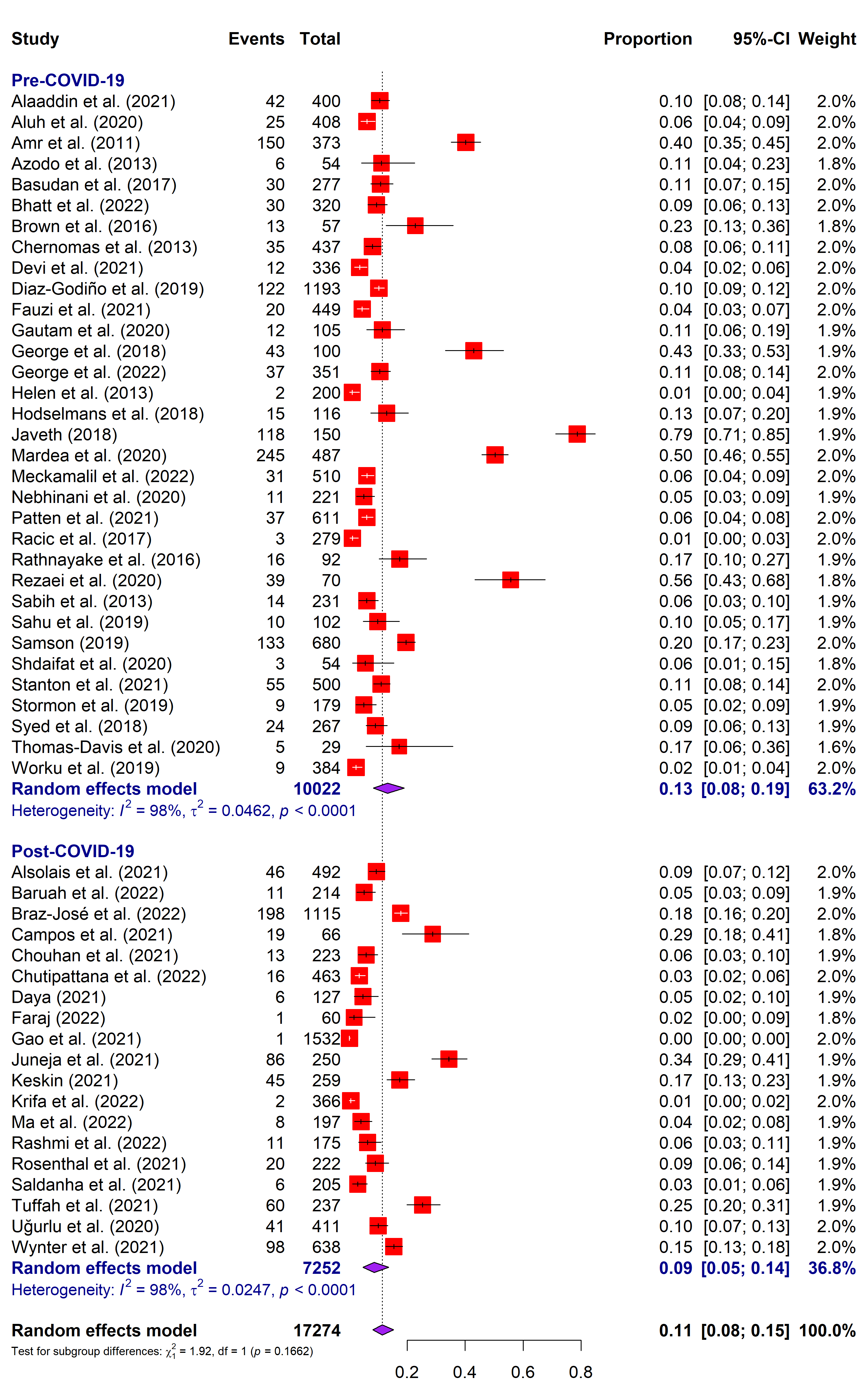
**

**Fig. S48** Subgroup analysis of global prevalence (95% confidence interval) of severe stress symptoms by data collection period.


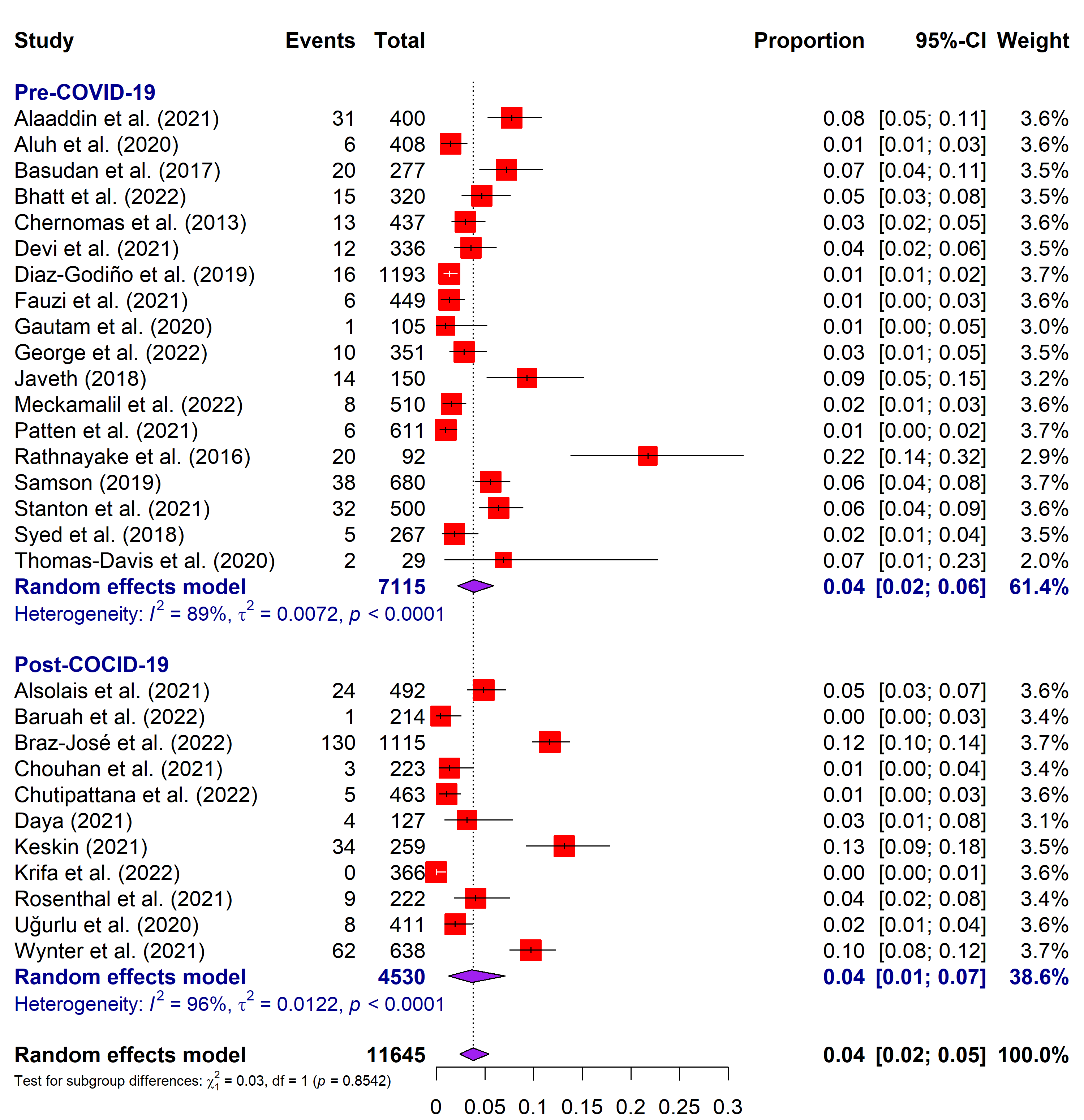


**Fig. S49** Subgroup analysis of global prevalence (95% confidence interval) of extremely severe stress symptoms by data collection period.

**
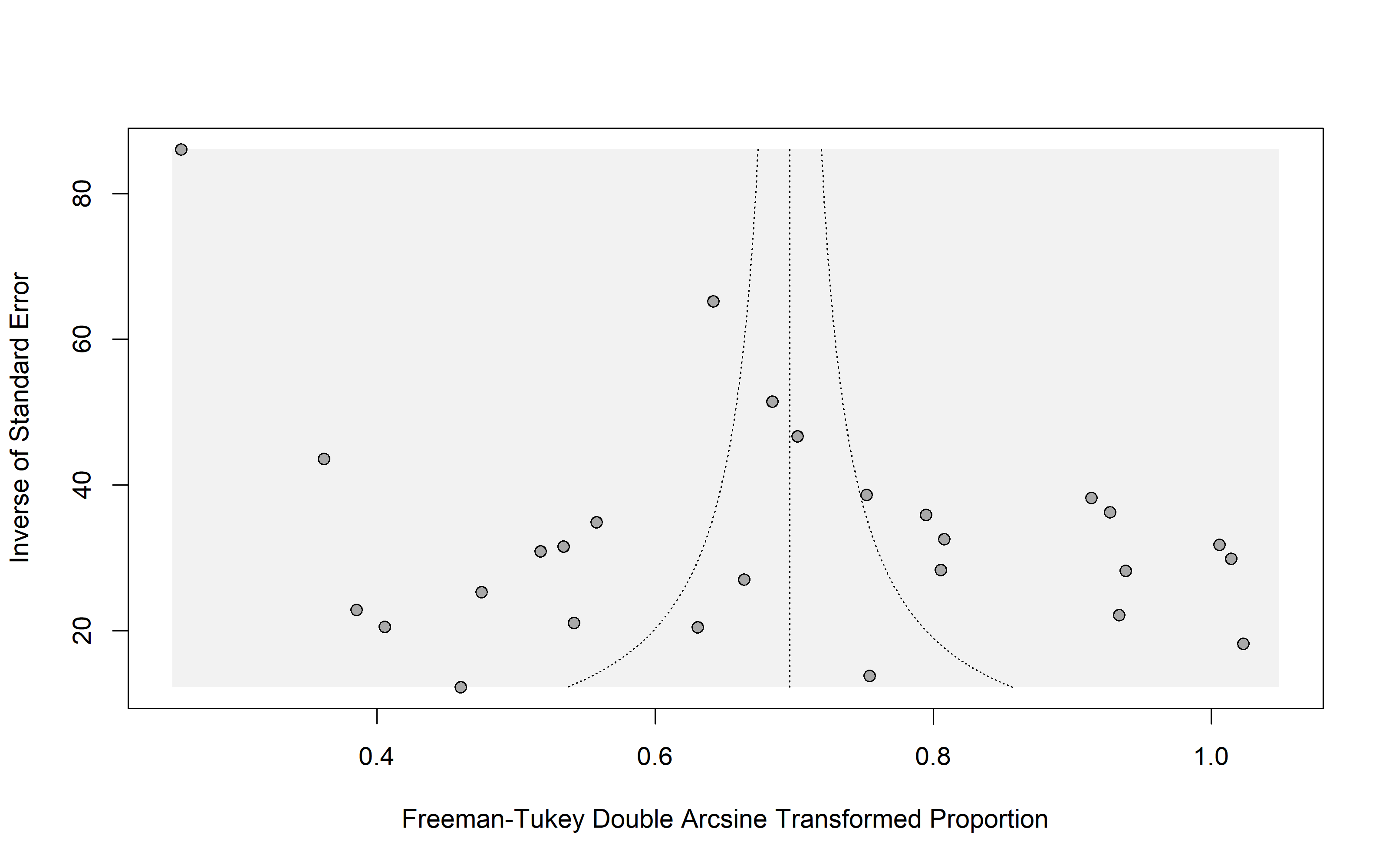
**

Linear regression test of funnel plot asymmetry

Test result: t = 2.75, df = 26, p-value = 0.0107

Sample estimates:

bias se.bias intercept se.intercept

10.1274 3.6852 0.3666 0.1014

Details:

- multiplicative residual heterogeneity variance (tau^2 = 66.0847)

- predictor: standard error

- weight: inverse variance

- reference: Egger et al. (1997), BMJ

**Fig. S50** Egger’s regression test and funnel plots of precision by the global prevalence of unspecified anxiety symptoms.

**
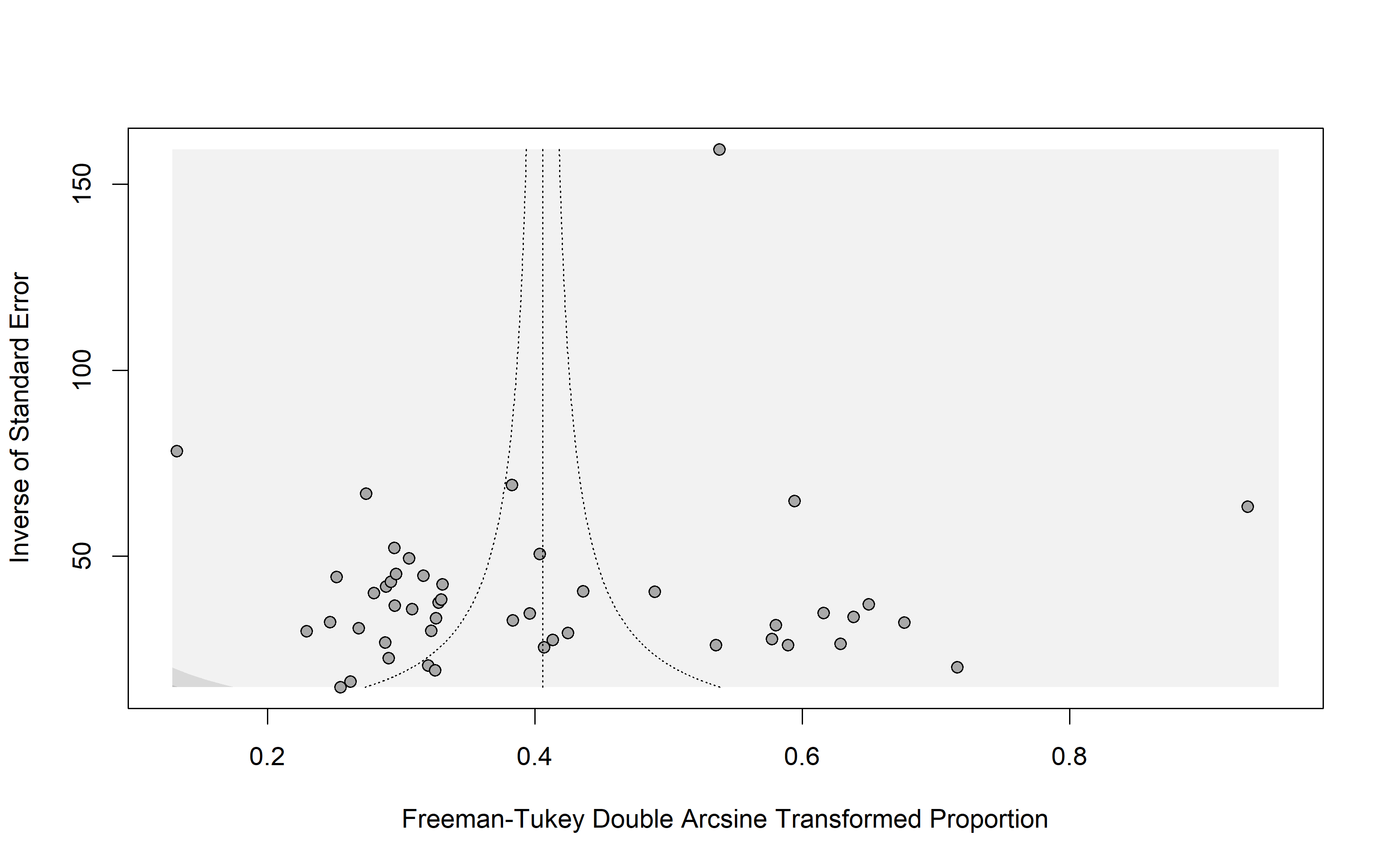
**

Linear regression test of funnel plot asymmetry

Test result: t = -1.44, df = 46, p-value = 0.1579

Sample estimates:

bias se.bias intercept se.intercept

-3.3627 2.3428 0.4962 0.0515

Details:

- multiplicative residual heterogeneity variance (tau^2 = 62.8134)

- predictor: standard error

- weight: inverse variance

- reference: Egger et al. (1997), BMJ

**Fig. S51** Egger’s regression test and funnel plots of precision by the global prevalence of mild anxiety symptoms.

**
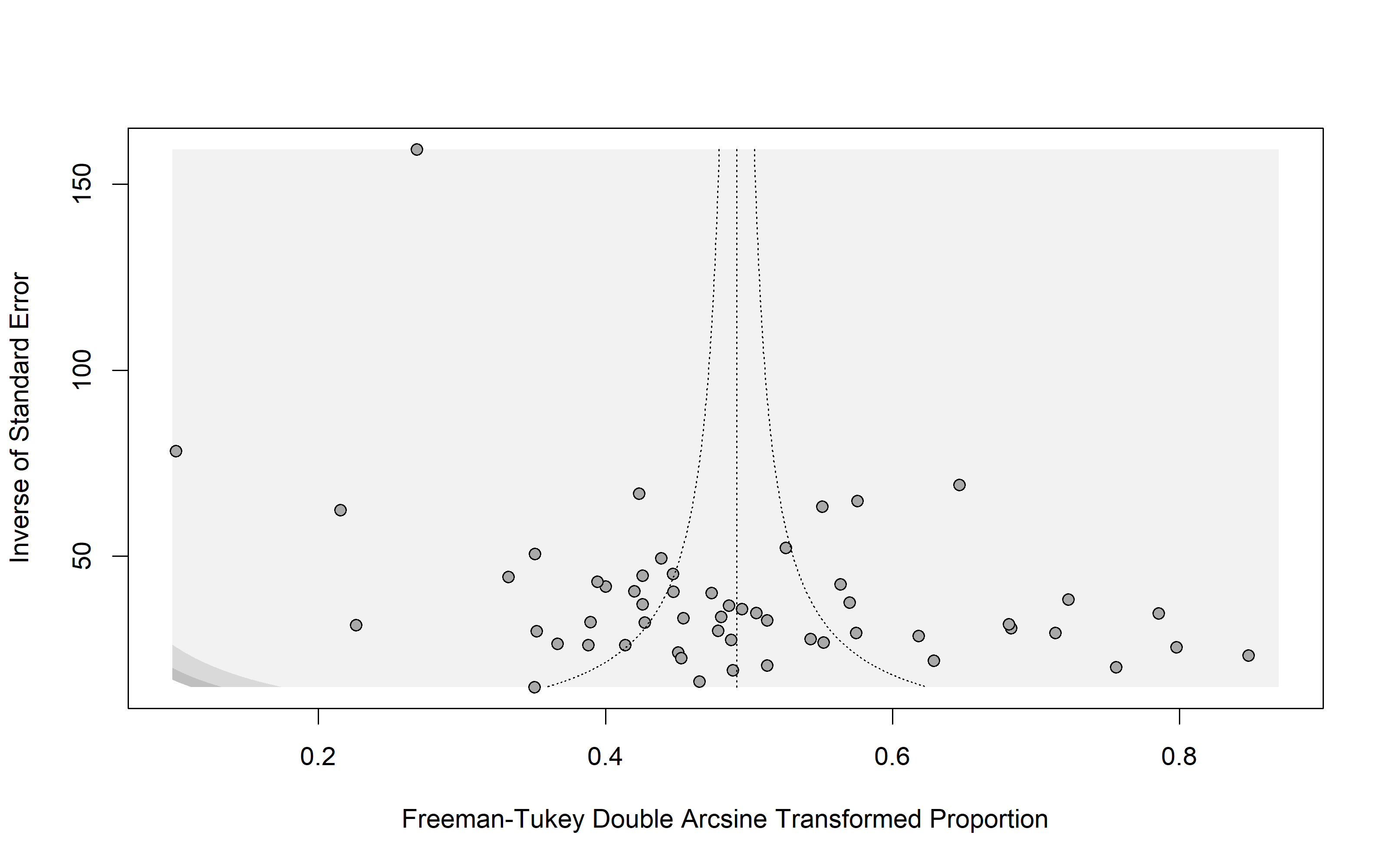
**

Linear regression test of funnel plot asymmetry

Test result: t = 4.78, df = 53, p-value < 0.0001

Sample estimates:

bias se.bias intercept se.intercept

8.1457 1.7053 0.2537 0.0386

Details:

- multiplicative residual heterogeneity variance (tau^2 = 37.5941)

- predictor: standard error

- weight: inverse variance

- reference: Egger et al. (1997), BMJ

**Fig. S52** Egger’s regression test and funnel plots of precision by the global prevalence of moderate anxiety symptoms.

**
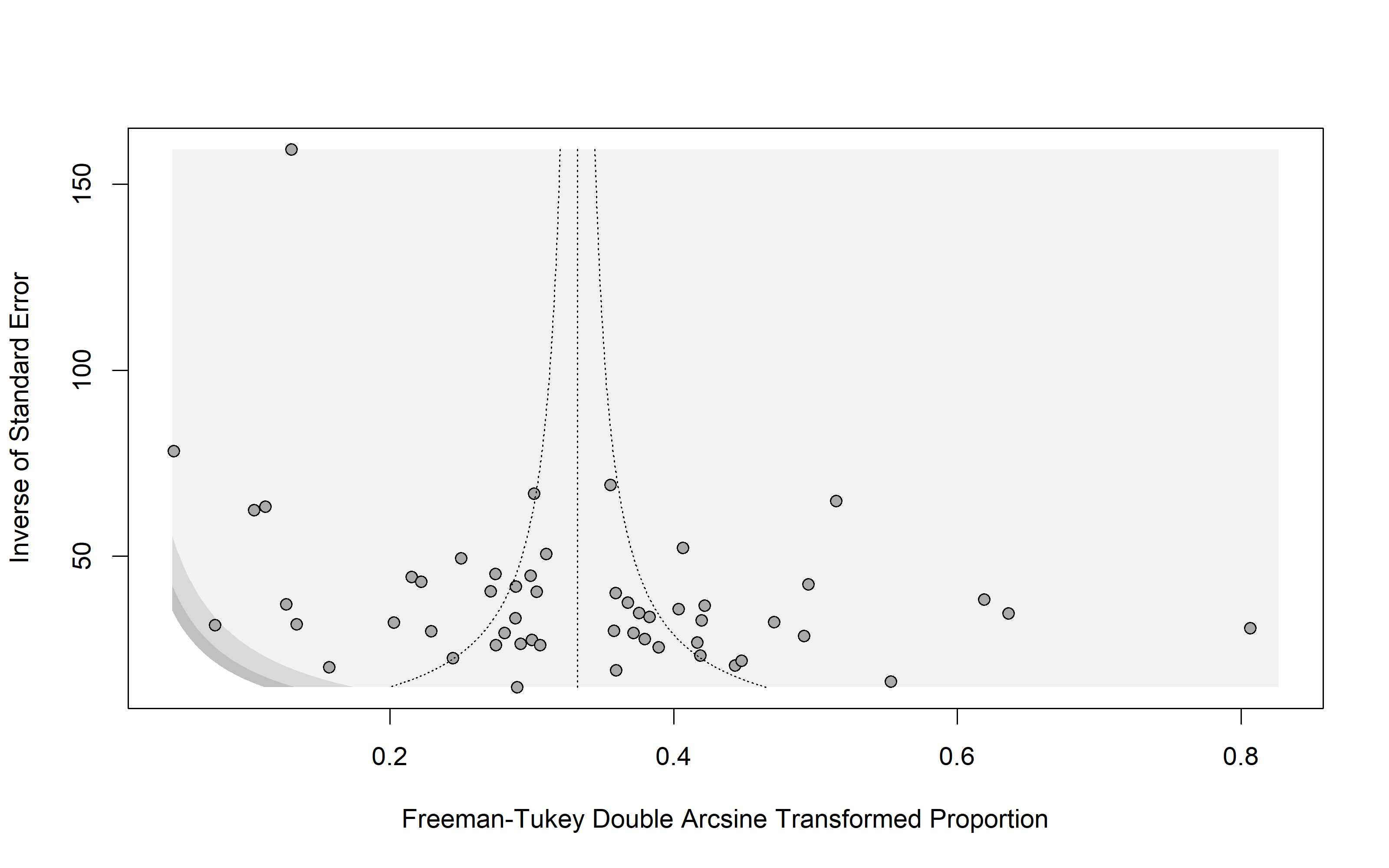
**

Linear regression test of funnel plot asymmetry

Test result: t = 4.55, df = 52, p-value < 0.0001

Sample estimates:

bias se.bias intercept se.intercept

7.4802 1.6435 0.1162 0.0369

Details:

- multiplicative residual heterogeneity variance (tau^2 = 34.1717)

- predictor: standard error

- weight: inverse variance

- reference: Egger et al. (1997), BMJ

**Fig. S53** Egger’s regression test and funnel plots of precision by the global prevalence of severe anxiety symptoms.

**
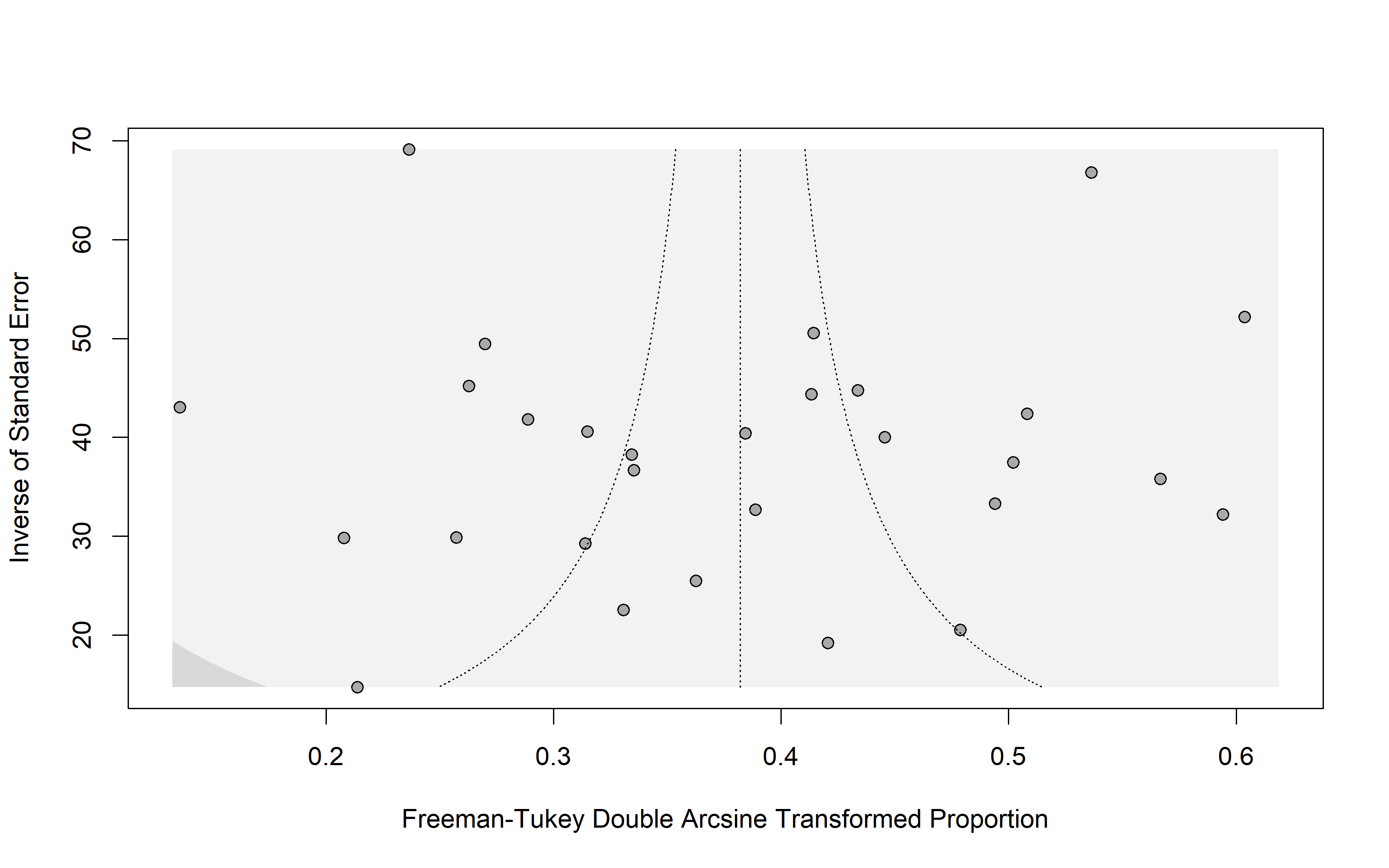
**

Linear regression test of funnel plot asymmetry

Test result: t = -0.22, df = 27, p-value = 0.8276

Sample estimates:

bias se.bias intercept se.intercept

-0.7110 3.2324 0.4033 0.0805

Details:

- multiplicative residual heterogeneity variance (tau^2 = 28.4132)

- predictor: standard error

- weight: inverse variance

- reference: Egger et al. (1997), BMJ

**Fig. S54** Egger’s regression test and funnel plots of precision by the global prevalence of extremely severe anxiety symptoms.

**
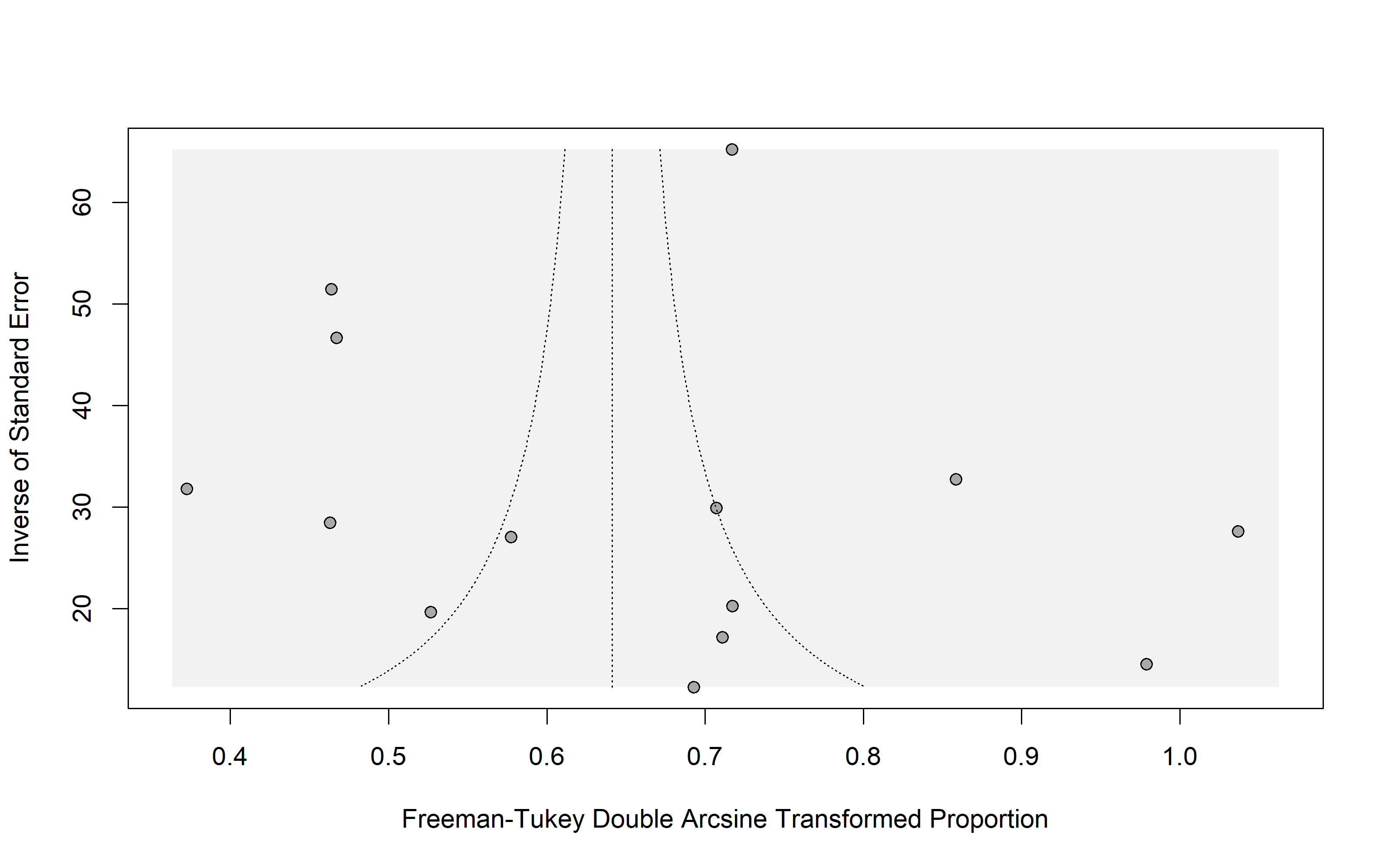
**

Linear regression test of funnel plot asymmetry

Test result: t = 0.54, df = 13, p-value = 0.5953

Sample estimates:

bias se.bias intercept se.intercept

2.1369 3.9240 0.5486 0.1172

Details:

- multiplicative residual heterogeneity variance (tau^2 = 40.2380)

- predictor: standard error

- weight: inverse variance

- reference: Egger et al. (1997), BMJ

**Fig. S55** Egger’s regression test and funnel plots of precision by the global prevalence of unspecified stress symptoms.


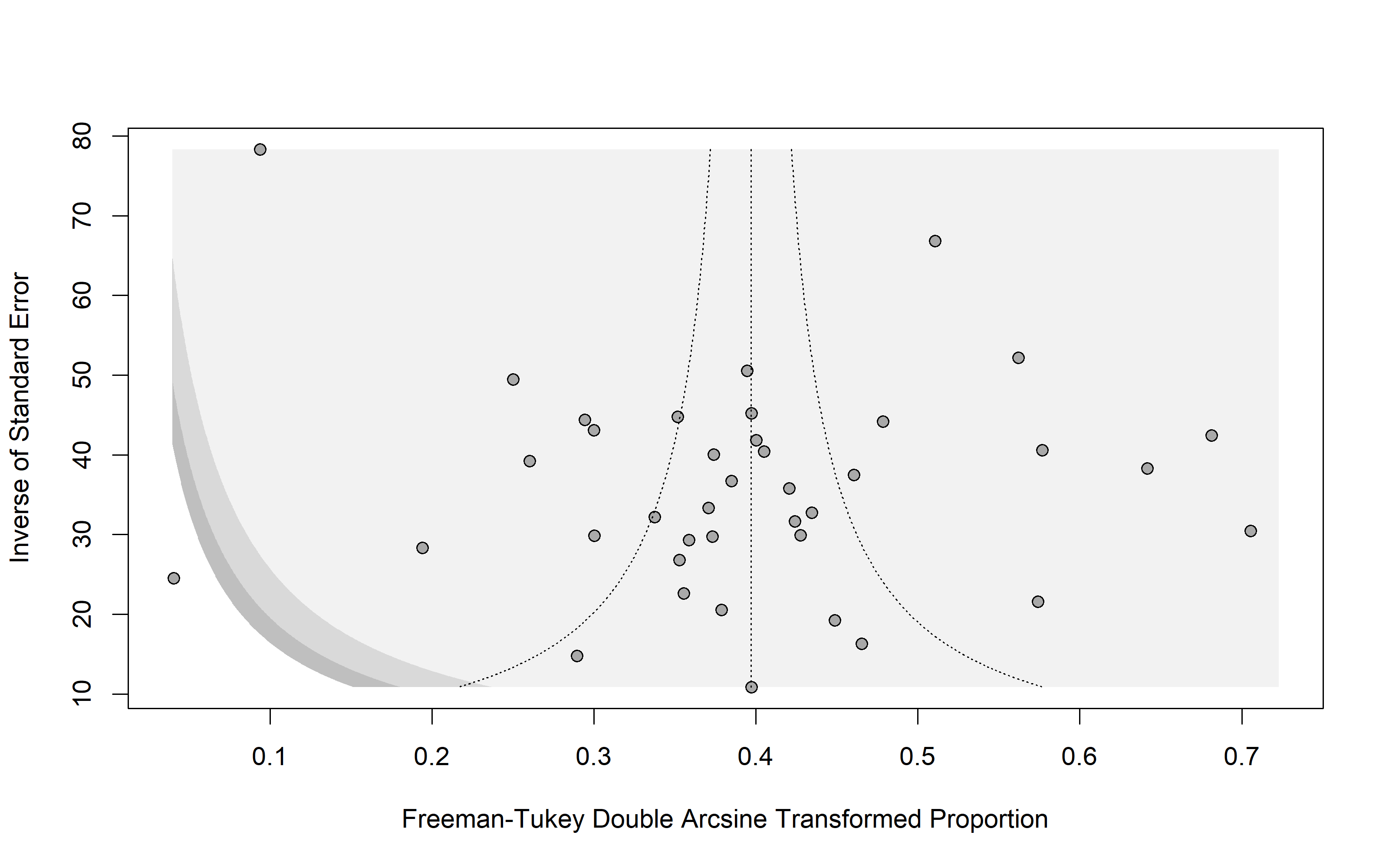


Linear regression test of funnel plot asymmetry

Test result: t = 1.08, df = 37, p-value = 0.2884

Sample estimates:

bias se.bias intercept se.intercept

2.9712 2.7583 0.3110 0.0723

Details:

- multiplicative residual heterogeneity variance (tau^2 = 35.4506)

- predictor: standard error

- weight: inverse variance

- reference: Egger et al. (1997), BMJ

**Fig. S56** Egger’s regression test and funnel plots of precision by the global prevalence of mild stress symptoms.

**
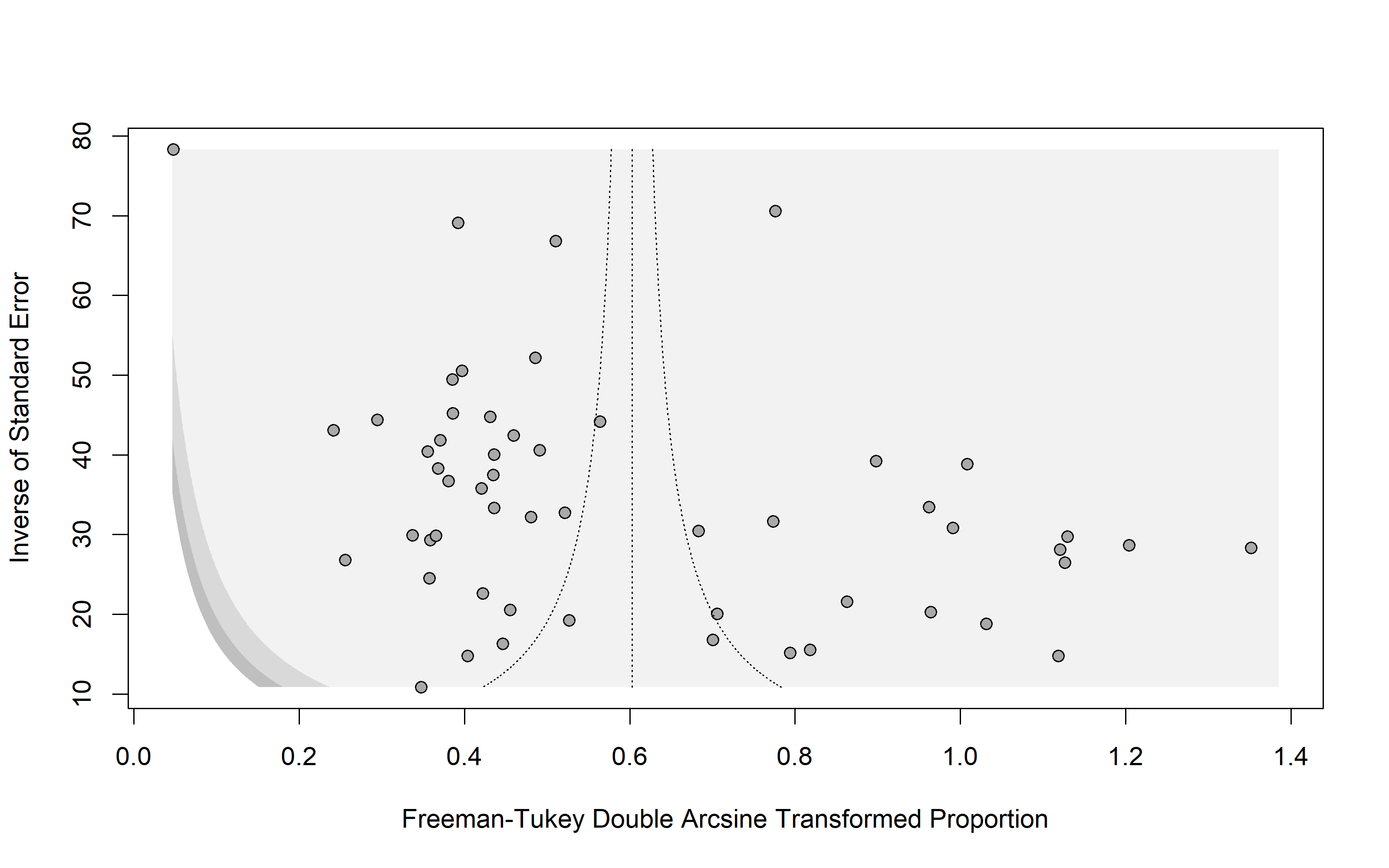
**

Linear regression test of funnel plot asymmetry

Test result: t = 3.05, df = 52, p-value = 0.0036

Sample estimates:

bias se.bias intercept se.intercept

10.2047 3.3503 0.2601 0.0902

Details:

- multiplicative residual heterogeneity variance (tau^2 = 94.3592)

- predictor: standard error

- weight: inverse variance

- reference: Egger et al. (1997), BMJ

**Fig. S57** Egger’s regression test and funnel plots of precision by the global prevalence of moderate stress symptoms.

**
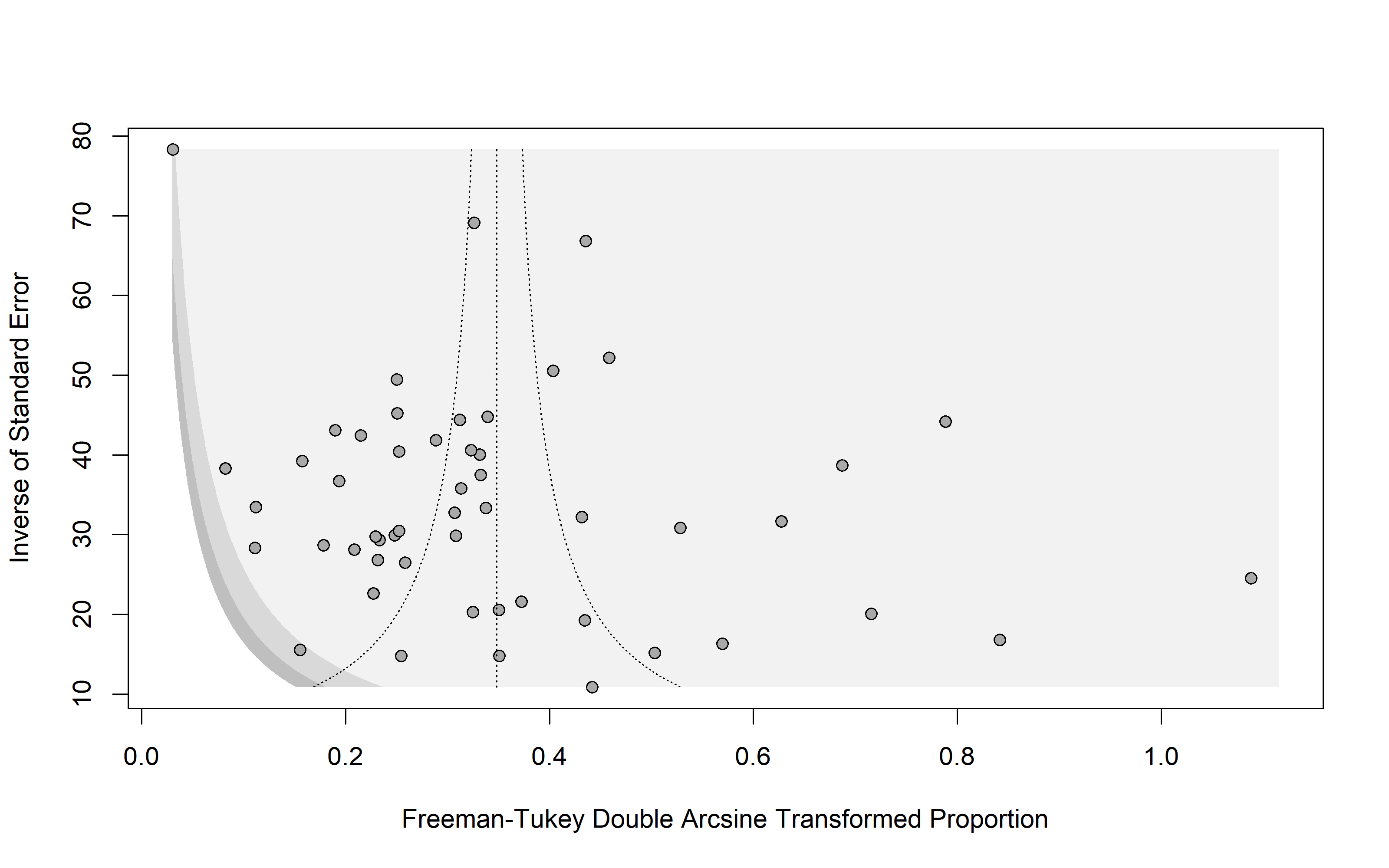
**

Linear regression test of funnel plot asymmetry

Test result: t = 1.56, df = 50, p-value = 0.1260

Sample estimates:

bias se.bias intercept se.intercept

3.7771 2.4275 0.2200 0.0665

Details:

- multiplicative residual heterogeneity variance (tau^2 = 44.4253)

- predictor: standard error

- weight: inverse variance

- reference: Egger et al. (1997), BMJ

**Fig. S58** Egger’s regression test and funnel plots of precision by the global prevalence of severe stress symptoms.

**
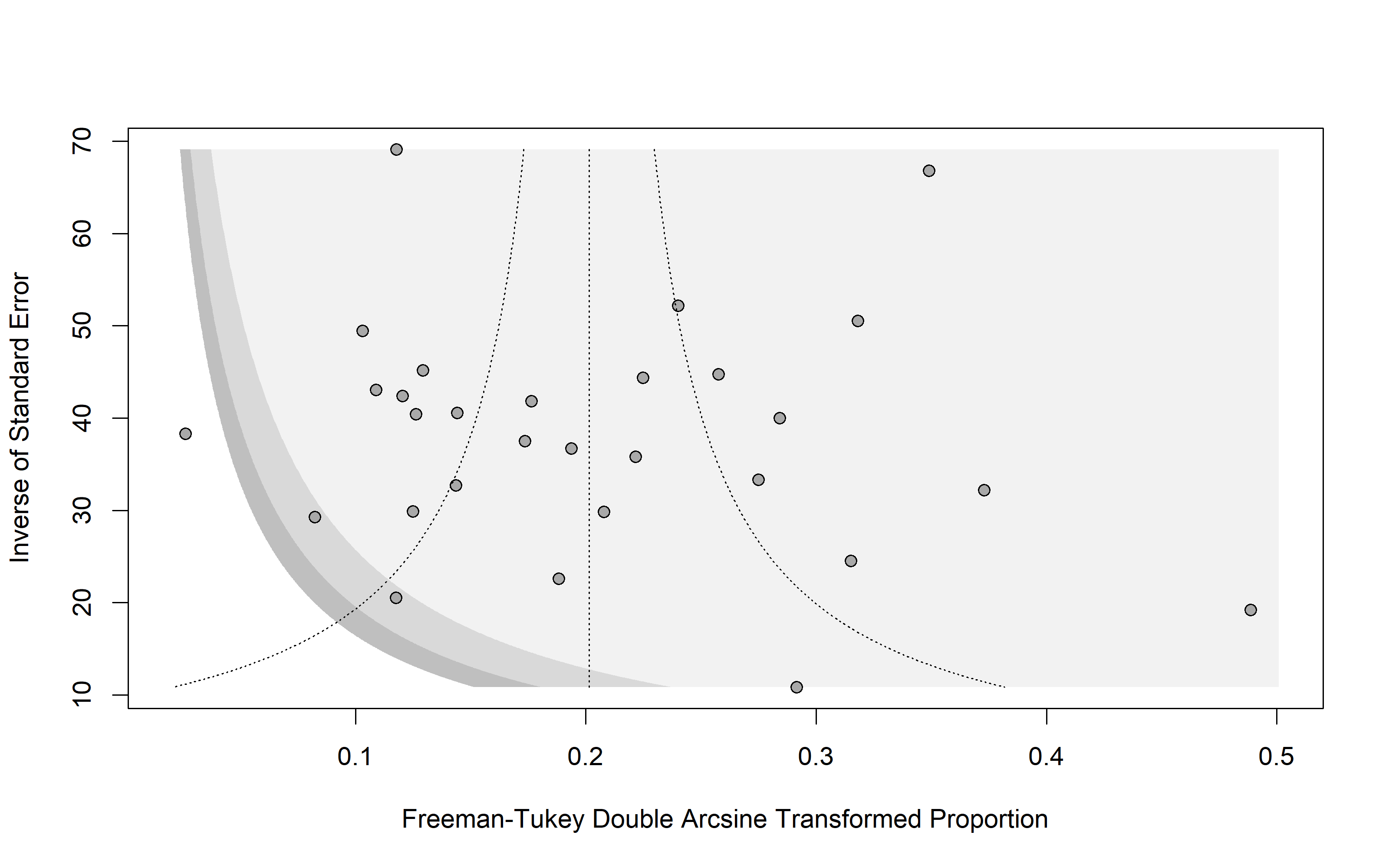
**

Linear regression test of funnel plot asymmetry

Test result: t = 0.02, df = 27, p-value = 0.9848

Sample estimates:

bias se.bias intercept se.intercept

0.0442 2.3060 0.1979 0.0575

Details:

- multiplicative residual heterogeneity variance (tau^2 = 15.2432)

- predictor: standard error

- weight: inverse variance

- reference: Egger et al. (1997), BMJ

**Fig. S59** Egger’s regression test and funnel plots of precision by the global prevalence of extremely severe stress symptoms.

**Table S5** GRADE assessment for overall quality

| № of studies | Certainty assessment | | | | | | № of individuals | Prevalence Rate, % (95% CI) | Certainty | Importance |
| --- | --- | --- | --- | --- | --- | --- | --- | --- | --- | --- |
|  | Study design | Risk of bias | Inconsistency | Indirectness | Imprecision | Other considerations |  |  |  |  |
| Unspecified Anxiety Symptoms | | | | | | | | | | |
| 28 | observational studies | not serious | very serious^a^ | not serious | serious^b^ | publication bias^c^ | 9,238 | 41% (33%, 50%) | ⨁◯◯◯ Very low | IMPORTANT |
| Mild Anxiety Symptoms | | | | | | | | | | |
| 48 | observational studies | not serious | very serious^a^ | not serious | not serious | No publication bias^d^ | 24,811 | 15% (12%, 19%) | ⨁◯◯◯ Very low | IMPORTANT |
| Moderate Anxiety Symptoms | | | | | | | | | | |
| 55 | observational studies | not serious | very serious^a^ | not serious | not serious | publication bias^c^ | 26,849 | 22% (19%, 26%) | ⨁◯◯◯ Very low | IMPORTANT |
| Severe Anxiety Symptoms | | | | | | | | | | |
| 54 | observational studies | not serious | very serious^a^ | not serious | not serious | publication bias^c^ | 26,705 | 10% (8%, 13%) | ⨁◯◯◯ Very low | IMPORTANT |
| Extremely Severe Anxiety Symptoms | | | | | | | | | | |
| 29 | observational studies | not serious | very serious^a^ | not serious | not serious | No publication bias^d^ | 1,1682 | 14% (11%, 17%) | ⨁◯◯◯ Very low | IMPORTANT |
| Unspecified Stress Symptoms | | | | | | | | | | |
| 15 | observational studies | not serious | very serious^a^ | not serious | serious^b^ | No publication bias^d^ | 4,196 | 36% (25%, 47%) | ⨁◯◯◯ Very low | IMPORTANT |
| Mild Stress Symptoms | | | | | | | | | | |
| 39 | observational studies | not serious | very serious^a^ | not serious | not serious | No publication bias^d^ | 14,672 | 15% (12%, 18%) | ⨁◯◯◯ Very low | IMPORTANT |
|  | | | | | | | | | | |
| Moderate Stress Symptoms | | | | | | | | | | |
| 54 | observational studies | not serious | very serious^a^ | not serious | not serious | publication bias^c^ | 19,111 | 32% (25%, 40%) | ⨁◯◯◯ Very low | IMPORTANT |
| Severe Stress Symptoms | | | | | | | | | | |
| 52 | observational studies | not serious | very serious^a^ | not serious | not serious | No publication bias^d^ | 17,774 | 11% (8%, 15%) | ⨁◯◯◯ Very low | IMPORTANT |
| Extremely Severe Stress Symptoms | | | | | | | | | | |
| 29 | observational studies | not serious | very serious^a^ | not serious | not serious | No publication bias^d^ | 11,645 | 4% (2%, 5%) | ⨁◯◯◯ Very low | IMPORTANT |

#### CI: Confidence Interval

#### Explanations:

a. High heterogeneity between studies (*I^2^*>50%)

b. Some studies had small sample sizes.

c. Egger's test result *p*<0.05, showing publication bias

d. Egger's test result *p*>0.05, showing no publication bias
